# Genetic architecture of adult-plant resistance to stripe rust in bread wheat (*Triticum aestivum* L.) association panel

**DOI:** 10.3389/fpls.2023.1256770

**Published:** 2023-12-07

**Authors:** Genet Atsbeha, Tilahun Mekonnen, Mulugeta Kebede, Teklehaimanot Haileselassie, Stephen B. Goodwin, Kassahun Tesfaye

**Affiliations:** ^1^ Department of Applied Biology, School of Applied Natural Science, Adama Science and Technology University, Adama, Ethiopia; ^2^ Institute of Biotechnology, Addis Ababa University, Addis Ababa, Ethiopia; ^3^ Department of Plant Biology and Biodiversity Management, Addis Ababa University, Addis Ababa, Ethiopia; ^4^ USDA-Agricultural Research Service, Department of Botany and Plant Pathology, Purdue University, West Lafayette, IN, United States; ^5^ Bio and Emerging Technology Institute. Affiliated with the Institute of Biotechnology, Addis Ababa, University, Addis Ababa, Ethiopia

**Keywords:** genome wide association study, linkage disequilibrium, marker assisted breeding, novel loci, *Puccinia striiformis*, quantitative trait loci, yellow rust

## Abstract

Stripe rust, caused by *Puccinia striiformis* f. sp. *tritici*, is a severe disease in wheat worldwide, including Ethiopia, causing up to 100% wheat yield loss in the worst season. The use of resistant cultivars is considered to be the most effective and durable management technique for controlling the disease. Therefore, the present study targeted the genetic architecture of adult plant resistance to yellow rust in 178 wheat association panels. The panel was phenotyped for yellow rust adult-plant resistance at three locations. Phonological, yield, yield-related, and agro-morphological traits were recorded. The association panel was fingerprinted using the genotyping-by-sequencing (GBS) platform, and a total of 6,788 polymorphic single nucleotide polymorphisms (SNPs) were used for genome-wide association analysis to identify effective yellow rust resistance genes. The marker-trait association analysis was conducted using the Genome Association and Prediction Integrated Tool (GAPIT). The broad-sense heritability for the considered traits ranged from 74.52% to 88.64%, implying the presence of promising yellow rust resistance alleles in the association panel that could be deployed to improve wheat resistance to the disease. The overall linkage disequilibrium (LD) declined within an average physical distance of 31.44 Mbp at r^2 ^= 0.2. Marker-trait association (MTA) analysis identified 148 loci significantly (*p* = 0.001) associated with yellow rust adult-plant resistance. Most of the detected resistance quantitative trait loci (QTLs) were located on the same chromosomes as previously reported QTLs for yellow rust resistance and mapped on chromosomes 1A, 1B, 1D, 2A, 2B, 2D, 3A, 3B, 3D, 4A, 4B, 4D, 5A, 5B, 6A, 6B, 7A, and 7D. However, 12 of the discovered MTAs were not previously documented in the wheat literature, suggesting that they could represent novel loci for stripe rust resistance. Zooming into the QTL regions in IWGSC RefSeq Annotation v1 identified crucial disease resistance-associated genes that are key in plants’ defense mechanisms against pathogen infections. The detected QTLs will be helpful for marker-assisted breeding of wheat to increase resistance to stripe rust. Generally, the present study identified putative QTLs for field resistance to yellow rust and some important agronomic traits. Most of the discovered QTLs have been reported previously, indicating the potential to improve wheat resistance to yellow rust by deploying the QTLs discovered by marker-assisted selection.

## 1 Introduction

Wheat (*Triticum aestivum* L.) is one of the major staple food crops in the world, providing 21% of the total energy and 20% of the protein demand for approximately 4.5 billion people globally (Ramadas et al., 2012). In 2021, approximately 221 million ha of the world was covered with wheat, with a total production and productivity of 771 million tonnes and 3.5 tonnes/ha, respectively (FAOSTAT, 2021). In Ethiopia, wheat is one of the strategic food security crops (Bezabeh et al., 2015), ranking fourth after teff (*Eragrostis tef*), maize (*Zea mays*), and sorghum (*Sorghum bicolor*) in area coverage and third after maize and teff in total production (CSA, 2019). In 2021, approximately 1.95 million ha of land was grown with wheat with a total national production and productivity of 5.2 million tons (FAOSTAT, 2021). Despite its significant contribution, the current national average wheat productivity of 2.67 t/ha is far below the global average of 3.5 t/ha. Fungal diseases such as stem rust, stripe rust or yellow rust, and Septoria tritici blotch (STB), which are caused by the rapidly evolving pathogens *Puccinia graminis* f. sp. *tritici*, *P. triticina*, and *Zymoseptoria tritici*, respectively, are the major bottlenecks for wheat production in Ethiopia. If not controlled, rust diseases have the potential to cause 50-100% wheat yield loss (Chen, 2005) which is estimated to be 5.5 billion USD globally per annum (Beddow et al., 2015).

Stripe rust, often known as yellow rust, is a devastating wheat disease that affects cooler (2-15°C) wheat-growing regions of the world (Roelfs et al., 1992). It is brought on by the biotrophic fungal pathogen *Puccinia striiformis* f.sp. *tritici*. Stripe rust disease continues to be a significant global limitation on wheat production. It impacts leaves, where the ensuing damage to photosynthetic tissues causes a reduction in the efficiency of light absorption and radiation usage (Bouvet et al., 2022). Furthermore, yellow rust limits yield by reducing the green leaf area, which supplies sugar to the developing seed. This is due to the fact that flag leaves and second leaves are the most important leaves for producing sugar for the developing grain (Murray et al., 2005). Since flag leaf alone accounts for more than 70% of grain filling, its infection with stripe rust results in significant yield loss (Marsalis and Goldberg, 2006). Moreover, yellow rust spores migrate quickly and may travel long distances to produce diverse populations, making it difficult to control the distribution of the disease (Vergara-Diaz et al., 2015).

Yellow rust is the most widespread disease in the highlands of Ethiopian as a result of which 36% to 100% wheat yield loss has been reported (Badebo and Bayu, 1992; Hailu and Fininsa, 2009; Ayele and Muche, 2019). In the highlands and mid-altitude regions of Ethiopia, yellow rust was to blame for the collapse of the dominant wheat varieties, including Laketch in 1977, Dashen (a well-liked high-yielding variety with the Yr9 gene) in 1988 and 1994, Wabe in 1998 (Badebo and Bayu, 1992), and Galema and Kubsa in 2010 (Tadesse et al. (2018). In 2010, stripe rust epidemics occurred in all major wheat-growing areas of Ethiopia, causing large yield losses (Solh et al., 2012; Dembel, 2014). Following the widespread 1950s stripe rust epidemic, several studies have been carried out related to its epidemiology and management. The most economically efficient strategy for controlling stripe rust is the use of resistant cultivars (Chen, 2005). Growing resistant cultivars to combat stripe rust disease is a dependable, efficient, and environmentally friendly strategy (Xu et al., 2013). Moreover, due to the ongoing development and selection of rust races, it is always preferable to look for additional sources of potent rust resistance genes. As a result, it requires the discovery and use of new resistance genes to defeat the dominant pathogenic races. As of now, 67 all-stage resistance (seedling and adult-plant resistance) genes and 80 race-specific/seedling yellow rust resistance genes (Yr) have been reported (Wang and Chen, 2017). According to Kankwatsa et al. (2017) and Yuan et al. (2018), adult plant resistance to pathogen mixtures is thought to be reliable and effective in field conditions. Since conventional breeding methods have been viewed as slow and ineffective despite their significant contributions to crop improvement (Liu et al., 2017), molecular markers are now often utilized in plant breeding to speed up crop improvement efforts. As a result, the effectiveness of breeding disease-resistant wheat can be increased by identifying molecular markers that are strongly connected to Yr genes, or quantitative trait loci (QTLs) (Miedaner and Korzun, 2012; Ayana et al., 2018).

A genome-wide association study (GWAS) is a powerful, fast, and economical approach to determining genomic regions associated with complex quantitative phenotypic variation (Yuan et al., 2018). GWAS enables us to dissect genes or QTLs underlying important traits using single nucleotide polymorphisms (SNPs) derived from whole-genome sequencing (Liu et al., 2017; Nadeem et al., 2021). Hence, its advancement is correlated with the development of high-throughput sequencing technology (Huang et al., 2019). Genotyping-by-sequencing (GBS) is a reasonably priced genetic screening method for discovering novel SNPs. GBS includes restriction digestion followed by high-throughput sequencing of a subset of a complex genome, which enables the generation of high-density SNPs at a lower cost (Bernardo et al., 2015). Moreover, GWAS has been used to dissect disease resistance’s genetic foundations in a range of plant species, including maize (Rashid et al., 2018), Arabidopsis (Rajarammohan et al., 2018), sorghum (Adeyanju et al., 2015), and soybean (Passianotto et al., 2017). For example, leaf rust, stem rust, stripe rust resistance, and septoria tritici blotch have all been studied in wheat using the GWAS technique (Kidane et al., 2017; Odilbekov et al., 2019; Mekonnen et al., 2021). According to Mengesha (2020), the application of the GWAS technique in breeding bread wheat resistant to yellow rust is largely missing in Ethiopia. Therefore, the present study was targeted at determining the genomic architecture of adult-plant resistance in 178 bread wheat germplasms to stripe rust in Ethiopia, which could be used in future marker-assisted breeding programs of wheat against yellow rust.

## 2 Methodology

### 2.1 Plant materials and evaluation of stripe rust resistance in adult plants

A total of 178 bread wheat germplasms (including 164 recombinant inbred lines received from the International Maize and Wheat Improvement Center (CIMMYT-Mexico) and 13 commercial cultivars cultivated in Ethiopia) were used in the current study (**Supplementary Table 1**). The germplasms from CIMMYT were comprised of six genotype lines from the National Variety Trial, five from the Adaptation Trial, 34 from the High Rain Wheat Screening Nursery, 49 from the International Bread Wheat Screening Nursery, 53 from the International Septoria Observation Nursery, 14 from the High Rain Wheat Yield Trial, and the remaining three genotypes were from PVT (preliminary variety trial). The bread wheat germplasms were evaluated across three locations (**Table 1**), namely, at Kulumsa Agricultural Research Centre (KARC) (8° 02’ N/39° 15 ‘ E), Meraro (substation of KARC) (07° 24’ 27 N/39° 14’ E), and Holeta Agricultural Research Centre (HARC) (9° 3’ N/38° 30’ E) in the 2020/21 main cropping season. The wheat genotype King-bird (G40) was used as the standard check in the field evaluation. The experiment was set up using an alpha-lattice design with two replications, six sub-blocks, and 30 entries per sub-block per replication, with two rows per entry (**Supplementary Table 2**). Each accession was sowed manually at 1m length, 0.20 m spacing between rows, and 0.4 m between entries. Spaces between blocks and replications were maintained at a 1.50 m distance. All trials were seeded with 150 kg of seeds per hectare, fertilized with 100 and 75 kg of N and P2O5 each, and weeded three times by hand-picking. To provide enough disease pressure, the sensitive cultivar “Morocco” was sown as a diffuser row throughout the length of the blocks.

**Table 1:** Coefficient of infection values in wheat genotypes evaluated in three individual environments.

Trait	Environment	Mean	Range	SD	Pr > F
YRPH	E1 (Holeta)	2.54	5.9	7.31	0.0017^*^
	E2 (Kulumsa)	8.30	14.5	7.25	<.0001^***^
	E3(Meraro)	15.43	24	9.86	<.0001^***^
YRH	E1 (Holeta)	4.66	24.5	10.39	<.0001^***^
	E2 (Kulumsa)	22.69	33	10.94	<.0001^***^
	E3(Meraro)	36.06	71	13.8	<.0001^***^
YRF	E1 (Holeta)	7.59	25.6	13.7	0.0001^**^
	E2 (Kulumsa)	28.52	53	11.86	<.0001^***^
	E3(Meraro)	47.64	86	15.24	<.0001^***^
YRMM	E1 (Holeta)	9.64	25.5	15.44	<.0001 ^***^
	E2 (Kulumsa)	30.81	34	14.13	<.0001 ^***^
	E3(Meraro)	57.30	72.5	17.02	<.0001^***^
YRM	E1 (Holeta)	12.32	25	30.45	0.0049^*^
	E2 (Kulumsa)	19.51	22.5	7.22	<.0001^***^
	E3(Meraro)	60.24	67	17.2	<.0001^***^
	E1 (Holeta)	7.35	12.4	12.49	<.0001^***^

YRPH, coefficient of infection at pre-heading; YRH. coefficient of infection at heading; YRF, coefficient of infection at flowering; YRMM, coefficient of infection at mid-maturity; YRM, coefficient of infection at maturity; SD, standard deviation; ***very highly significant at p< 0.001, **highly significant at p< 0.001, *significant at p< 0.05.

The modified Cobb scale (Peterson et al., 1948) was used to measure the disease severity (DS), with values ranging from 0% to 90%. Moreover, the genotypes’ field response (FR) to stripe rust infection was scored following the procedure described by Roelfs et al. (1992) as Immune = no uredinia or other macroscopic sign of infection; R = resistant, small uredinia surrounded by necrosis; MR = moderately resistant, medium to large uredinia surrounded by necrosis; MRMS=moderately resistant to moderately susceptible; MS = moderately susceptible, medium to large uredinia surrounded by chlorosis; or S = susceptible, large uredinia without necrosis or chlorosis. For statistical analysis, each replicate’s genotype responses for immune, resistant, moderately resistant, moderately resistant to moderately susceptible, moderately susceptible, and susceptible were translated into the values 0, 0.2, 0.4, 0.6, 0.8, and 1, respectively (Elbasyoni et al., 2019). When the disease severity was greater than or equal to 50% and a susceptible reaction (S) was seen on the spreader rows, the plant resistance response was scored, and this resulted in a combined value of 50S (Alemu et al., 2021). In addition to the disease data, important agronomic traits such as days to 50% heading (HD), days to 50% flowering (FD), days to maturity (MD), thousand kernel weight (TKW), grain yield per plot (GYPP), plant height (PH), spike length (SL), flag leaf area (LA), number of spikelets per spike (NSs/S), number of kernels per spike (NK/S), number of kernels per spike (NK/S), and spike weight (SW) were recorded. Days to heading, flowering, and maturity were recorded for whole plots when 50% of the plants reached the corresponding Zadock’s growth stages. Plant height and spike length were measured at the physiological maturity stage in five randomly selected and tagged plants from the middle rows of each entry. Data on the number of spikelets per spike, the number of kernels per spikelet, and the total number of kernels per spike were determined from the five randomly tagged plants per entry per replication by collecting their spikes separately. These yield and yield-related data were taken from the two rows of each plot and converted to kilograms per hectare (kg ha^−1^) at 12.5% moisture content using plot size as a factor.

### 2.2 Data analysis

#### 2.2.1 Phenotype data analysis

The coefficient of infection (CI), which indicates the combined reaction of the genotypes for the disease, was created by multiplying the disease severity score (DS=0 - 90) by the genotype field response values (FR=0.0 - 1). An analysis of variance (ANOVA) for each and combined environments was carried out using SAS version 9.4 (SAS, 2013) based on a linear mixed model (LMM). In the analysis of variance for individual environments, genotype and the incomplete block were considered as fixed and random factors, respectively, to compute the ANOVA for each location. The observed phenotypic response of the i^th^ genotype in the j^th^ replication and the l^th^ sub-block was calculated for an individual environment using the following model:


$$y_{ijl}=µ+{\text{g}}_{i}+\gamma_{j}+{\text{bl}}_{\left(j\right)}+\;\epsilon_{\text{ij}}$$

Where, y_ijl_ = measured phenotype, g_i_ = fixed effect of the i^th^ genotype, μ = grand mean, γ_j_ = effect of the j^th^ replication, bl_(j)_ = random effect of the i^th^ block nested within the j^th^ replication, and ε_ijl_ = random error term.

Combined ANOVA was calculated by treating the genotype as a fixed variable and the incomplete block and location as random effects as LMM:


$${\text{Y}}_{jklm}=\mu +{\text{g}}_{m}+\gamma_{jk}+{\text{e}}_{j}+\;{\text{b}}_{jkl}+{\left(\text{ge}\right)}_{jm}+\epsilon_{\text{jklm}}$$

where Y_jklm_ = observed response of genotype m, replication k of block l at location j; b_jkl_ = random effect of block l nested with replication k in location j and is ~ NID(0, δ^2^_b_); g_m_ = fixed effect of genotype m; μ = grand mean; r_jk_ = effect of replication k in location j; e_j_ = random effect of location j and is ~ NID(0, δ^2^_e_); (ge)_jm_ = random effect of the interaction between genotype m and location j and is ~ NID(0, δ^2^_ge_); and ε_jklm_ = random residual effect and ~ NID(0,δ^2^_ε_). Variance components were computed.

The genotypes were classified into resistant, intermediate, and susceptible groups based on the average values of DS, FR, and CI for the pooled data from all environments at each growth stage. Bar plots showing DS, FR, and CI of the 178 genotypes were plotted using the ggplot2 (Wickham, 2016) and ggpubr (Kassambara, 2023) packages in R software (R Core Team, 2020). The yellow rust resistance traits’ broad sense heritability (H^2^) at the environmental level was calculated through an analysis of variance using the following formula:


$${\text{H}}^{2}\;=\frac{\;\delta^{2}\text{g }}{\delta^{2}\text{g }+\;\delta^{2}\epsilon /\text{r }}$$

Broad-sense heritability across environments was predicted by the formula:


$${\text{H}}^{2}\;=\frac{\delta {\;}^{2}\text{g }}{\delta^{2}\text{g }+\;\delta^{2}\text{ge}/\text{l }+\delta^{2}\epsilon /\text{lr }}$$

where, δ^2^g is the genotypic variance, δ^2^ge is the genotype-by-location interaction variance, δ^2^e is the location variance, and l and r represent the numbers of locations and replicates, respectively. The virility package in the R for Windows Version 4.2.3 program was used to analyze the correlation analysis between the various parameters (Raj et al., 2020).

#### 2.2.2 Genomic DNA extraction and genotyping by sequencing

For genomic DNA extraction, the study wheat plants were grown in greenhouse conditions at the National Agricultural Biotechnology Research Canter (NABRC), Holeta, 29 km west of Addis Ababa. Leaves of 2-week-old seedling leaves were collected into 96 well samples collecting plates and dried at 50°C overnight. The samples were sent to BecA-ILRI, Hub laboratory in Nairobi, Kenya for SNP genotyping using Diversity Arrays Technology sequencing (DArTseq™ technology) with the support of the Integrated Genotyping Service and Support (IGSS) program. Genomic DNA was extracted using a Nucleomag Plant Genomic DNA extraction kit (MACHEREY-NAGEL GmbH & Co. KG, Germany) according to the manufacturer’s procedure. DNA quality and quantity were assessed using a Nanodrop spectrophotometer and 1% agarose gel electrophoresis, respectively. SNP genotyping was done by utilizing GBS technology, using an Illumina HiSeq2500 following the procedure described by Elshire et al. (2011). Genome complexity reduction was carried out by digesting genomic DNA with ApeKI, a type II restriction endonuclease that recognizes a degenerate 5-bp sequence (GCWGC, where W is A or T). For genotyping, the library was constructed by ligating common and barcode adaptors to the sticky ends of each fragment. The adapter-ligated fragments were PCR amplified, and then sequenced by synthesis using single-read sequencing runs for 77 bases. Next-generation sequencing of the GBS library was carried out using an Illumina HiSeq2500 lane following the manufacturer’s protocol.

#### 2.2.3 Quality control and SNP calling

Based on alignment with the reference genome of Chinese Spring Wheat RefSeq v1.0 (IWGSC, 2018), the DArTSeq SNP markers were scored using the DArTsoft14 software implemented in the KDCompute plug-in system created by Diversity Arrays Technology (https://kdcompute.seqart.net/kdcompute/login). SilicoDArT and SNP markers, which were both scored in binary ways (1/0), reflect the existence or absence of marker data in the genomic representation of each sample (Akbari et al., 2006). Marker quality control was maintained by filtering or removing mono-morphic -markers, markers with lower call rates (>30% missing), and markers with minor allele frequencies (MAF<5%). Genotypes with >30% missing marker data were also removed from the analysis.

#### 2.2.4 Population structure analysis

Bayesian model-based clustering in STRUCTURE software version 2.3.4 was used to determine the population admixture pattern (Pritchard et al., 2000). The STRUCTURE program was run with the admixture model, correlated allele frequencies, a burn-in period of 10,000, and 50,000 Markov Chain Monte Carlo (MCMC) replications after burn-in for the hypothetical sub-population K from 1 to 10 with 10 iterations. The STRUCTURE HARVESTER version 0.6.92 was used to determine the optimum K value, according to Evanno et al. (2005). The clumpak beta version was used to determine the bar graph for the ideal K (Kopelman et al., 2015). Principal component analysis (PCA) was computed using Genome Association and Prediction Integrated Tools (GAPIT) software to determine the population spatial distribution and clustering.

#### 2.2.5 Genome-wide association study

GAPIT software was used to conduct a marker-trait association analysis (Lipka et al., 2012). GWAS was carried out for the coefficient of infection at five growth stages, including yellow rust resistance at pre-heading (YRPH), yellow rust resistance at heading (YRH), yellow rust resistance at flowering (YRF), yellow rust resistance at mid-maturity (YRMM), yellow rust resistance at maturity (YRM), and some significant agro-morphological traits such as days to 50% (FD), days to 90% maturity (MD), leaf area (LA), plant height (PH), number of kernels per spike (NKS), thousand-kernel weight (TKW), and grain yield (GY) in each individual environment and across all environments. The marker-trait association analysis involved a total of 6,788 robust SNPs with a call rate > 70% and MAF > 5%. Missing SNPs were imputed using optimal impute ver. 1.0.0 based on the KNN imputation method in the KDcompute_plugin system. The LD measure R^2^ ver.0.2.2 in KDcompute_plugin system was used to determine marker distribution on individual chromosomes.

TASSEL Ver. 5 (Bradbury et al., 2007) was used to calculate the pairwise LD measurements (r^2^ and *p-value*) between markers on each chromosome. A genome-wide scale LD decay scatter plot was produced by plotting r^2^ values against physical distance (bp) using GAPIT software. An r^2 ^= 0.2 was used as a cut-off to declare no LD between pairs of markers. The confidence range for establishing a discrete QTL for each chromosome was calculated using the physical distance at which the LD declined to the crucial r^2 ^= 0.2. If the distance between the significant SNP markers was smaller than the critical physical distance, they were assigned to the same QTL if they were on the same chromosome.

Bayesian information and Linkage-disequilibrium Iteratively Nested Keyway (BLINK) model, implemented in the GAPIT R package (Tang et al., 2016), was used to conduct GWAS. BLINK was selected because of its high computing efficiency and statistical power to control spurious associations due to population structure and kinship. BLINK presented the best Bayesian information criterion value for across and individual environments. Kinship (K) matrix was computed using Van-Raden (2008) method. The model’s fitness to control population structure and familial relatedness of the study samples was examined by observing using the quantile-quantile (QQ) plot created from -log10 *p-values*. Marker-trait associations (MTAs) were considered to be significant when they surpassed the significant threshold of the nominal p-values of 0.001 or -log10 (*p-values)* = 3. Manhattan plot and Q-Q were observed using the R package qqman (Turner, 2014) to identify significant MTAs. Candidate genes in the detected significant regions were annotated MTAs from the recently released IWGSC RefSeq Annotation v1 available at https://wheat-urgi.versailles.inrae.fr/SeqRepository/Annotations.

## 3 Results

### 3.1 Yellow rust infection and field response

**Figure 1:**
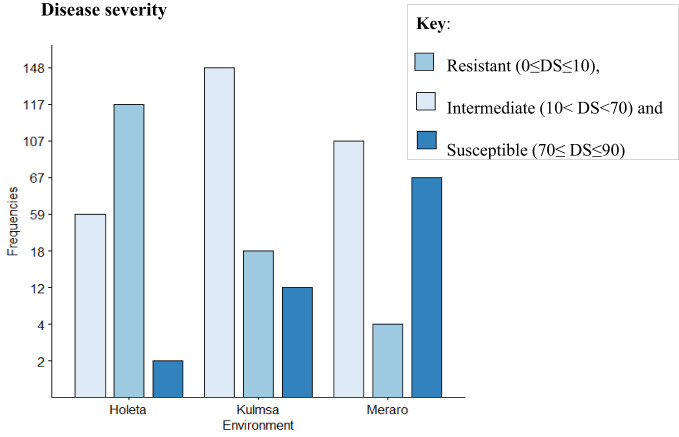
Yellow rust disease severity (DS) of 178 bread wheat genotypes at three locations. Clustering involved resistant (0≤DS ≤ 10), intermediate (10<DS<70), and susceptible (70≤DS ≤ 90).

**Figure 2:**
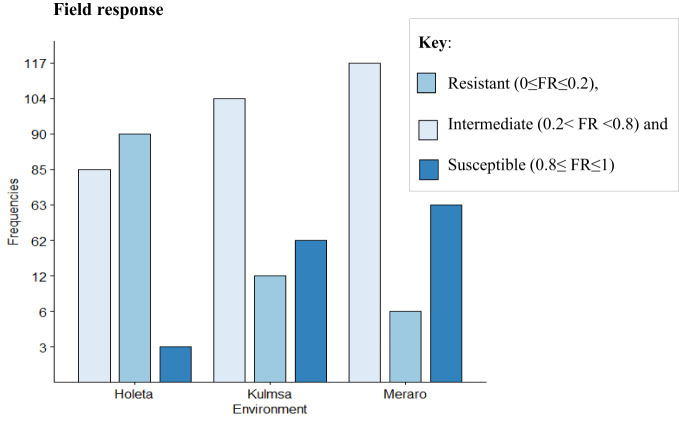
Field reaction response (FR) of 178 bread wheat germplasms to yellow rust: Resistant (0≤FR ≤ 0.2), intermediate (0.2<FR<0.8), and susceptible (0.8≤FR ≤ 1) groups.

**Figure 3:**
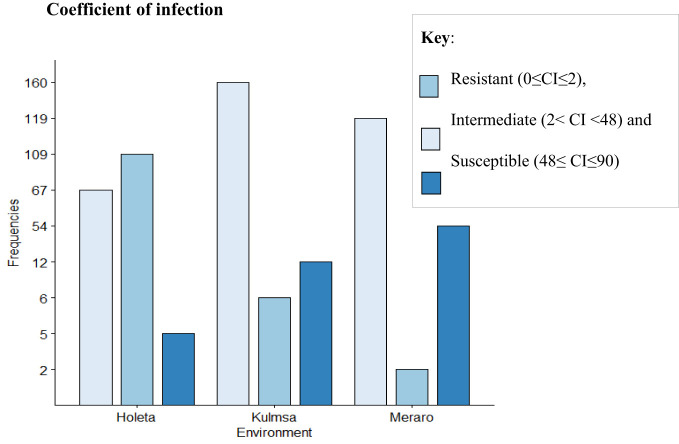
CI of yellow rust for 178 bread wheat genotypes obtained from field experiments in three environments. The genotypes were clustered as resistant (0≤CI ≤ 2), intermediate (2< CI<48), and susceptible (48≤ CI ≤ 90).

The panel was divided into resistant, intermediate, and susceptible groups based on DS, FR, and CI data that were pooled from all growth stages and environments (Liu et al., 2017). The panel showed variable reaction groups for DS, FR, and CI. For DS, 65.73% of the genotypes were resistant (0≤DS ≤ 10) at Hol (Holeta), 10.11% at Kul (Kulumsa), and 1.69% at Mer (Meraro) (**Figure 1**) with regard to FR, 96.6% of the genotypes joined the resistant group at Holeta, 50.56% at Kulumsa, and 6.74% at Meraro (**Figure 2**). Likewise, for CI, 60.11%, 3.37%, and 1.12% tested of the wheat genotypes were resistant at the Holetta, Kulumsa, and Meraro locations, respectively (**Figure 3**). Since CI is a combined representation of DS and FR (Liu et al., 2017), clustering based on this parameter resulted in 5 stable resistant genotypes (2.81%), 119 intermediate genotypes (66.85%), and 54 susceptible genotypes (32.02%) out of the 178 tested genotypes (**Figure 3**).

#### 3.1.2 Adult-plant resistance to yellow rust and broad-sense heritability

**Figure 4:**
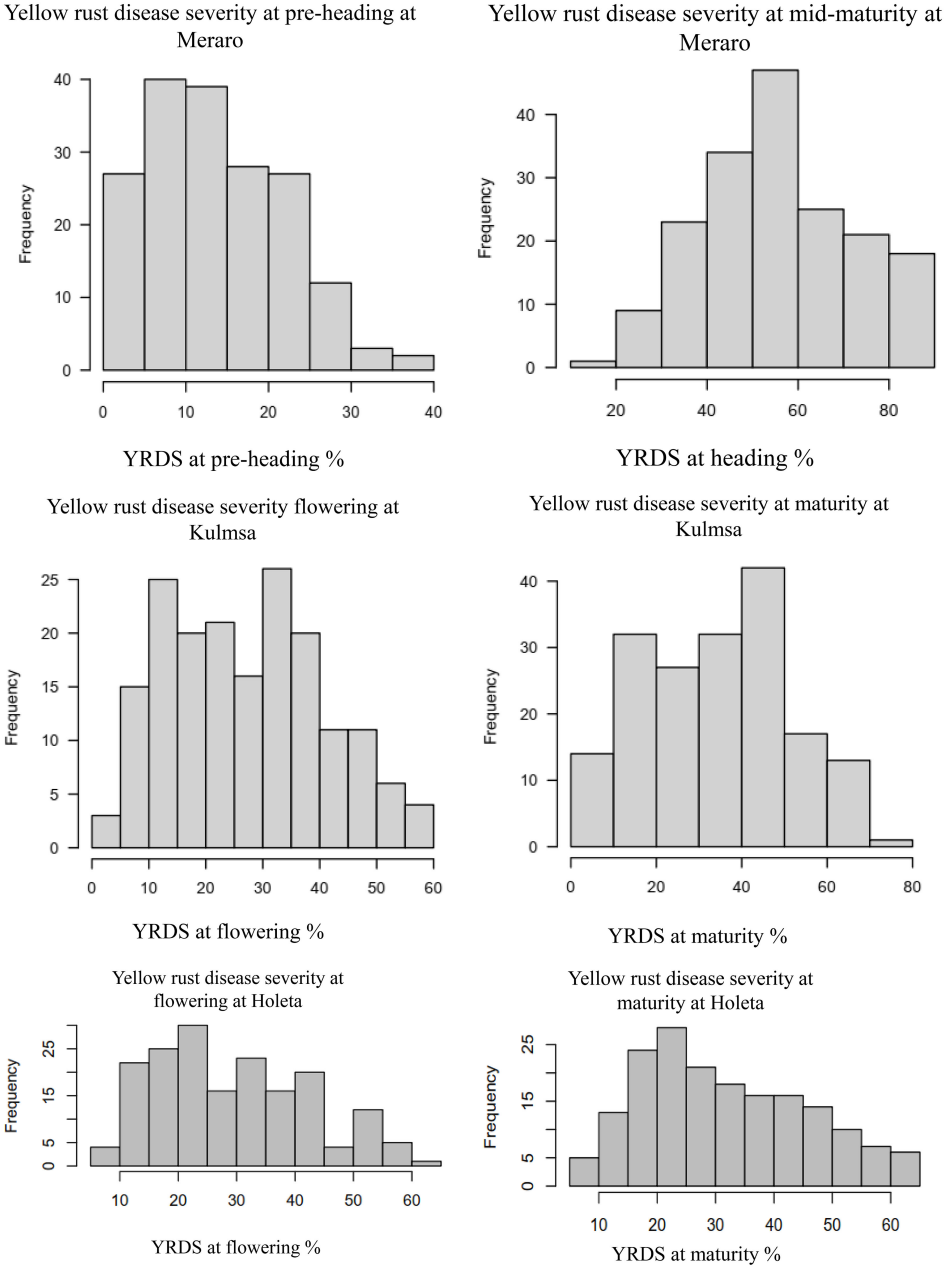
Frequency distribution of some yellow rust resistance traits for the combined data from three locations. The right and left ends of the bars indicate the uppermost and lowermost infection classes, respectively. The disease resistance at the pre-heading, heading, and mid-maturity stages and combined followed a virtually pseudo-normal distribution. YRPH, coefficient of infection at pre-heading; YRH, coefficient of infection at heading; YRF, coefficient of infection at flowering; YRMM, coefficient of infection at mid-maturity; YRM, coefficient of infection at maturity; and Kulmsa, Kulumsa.

Yellow rust coefficient of infection traits showed pseudo-normal distributions (**Figure 4**) in the investigated bread wheat genotype, indicating the quantitative nature of adult-plant yellow rust resistance (Kidane et al., 2017). The genotypes’ mean performance, standard deviations, range, and significance level for considered phenotypic traits were measured in each environment. The genotypes showed very highly significant differences (*p<* 0.001) was measured in each and across environments at all growth stages. The analysis revealed that the coefficient of infection in each environment showed an increased trend from pre-heading (2.54-15.43%) to maturity stages (12.32 - 60.24%). The highest (60.24%) mean severity values were registered at Meraro at the maturity stage, while the lowest infestation severity (2.54%) was recorded at the pre-heading stage at Holeta. The pooled data revealed that the coefficient of infection was the highest at Meraro (43.34%), followed by Kulumsa (21.97%), and the lowest at Holeta (7.35%) (**Table 1**).

For the coefficient of infection, the combined ANOVA revealed that the effects of genotype, location, and their two-way interactions (genotype x location) were very significant (*p<*0.0001) (**Table 2**). Moreover, blocking had a significant effect on genotype performance for yellow rust resistance. The ANOVA using pooled data revealed that genotypic variance (σ^2^g) and environmental variation (σ^2^e) were the major sources of the variation for the coefficient of infection variability among the tested wheat genotypes (**Table 3**). As per the scale of Robinson et al. (1949), yellow rust resistance showed moderate broad heritability (30% ≤H^2^ ≤ 60%) in all individual environments except for the pre-heading stage, where the broad sense heritability was lower (H^2 ^= 27.37%). Focusing the coefficient of infection on the narrow-sense heritability in individual environments revealed that yellow rust resistance is highly heritable (h^2 ^= 74.52-88.64%) (**Table 3**). The genetic and phenotypic coefficients of variation for variables related to the coefficient of infection ranged from 40.23% (YRMM) to 61.49% (YRPH) and 65.29% (YRMM) to 117.53% (YRPH), respectively (**Table 3**). At 5% selection intensity, the range of magnitude of the expected genetic gain as a percent of the mean varied from 48.76% (YRM) to 66.36% (YRPH), whereas the genetic advance for the coefficient of infection ranged from 5.81 (YRPH) to 16.66 (YRMM) (**Table 3**).

**Table 2:** Combined ANOVA for yellow rust coefficient of infection.

Source of variation	DF					
		YRPH	YRH	YRF	YRMM	YRM
Genotype	177	242.52^***^	707.82^***^	1051.76^***^	1288.05^***^	1325.01^***^
Replication	1	1322.25^***^	0.13 ^ns^	217.80^ns^	176.42^ns^	163.18^ns^
Incomplete block	5	177.54^***^	564.62^***^	647.87^***^	^652.17***^	704.71^*^
Location	2	15013.82^***^	89355.23^***^	144498.68^***^	205404.21^***^	240403.97^***^
Genotype^*^ location	358	85.28^*^	209.20^**^	274.82^**^	303.67^*^	541.58^**^

YRPH, coefficient of infection at pre-heading; YRH, coefficient of infection at heading; YRF, coefficient of infection at flowering; YRMM, coefficient of infection at mid-maturity; YRM, coefficient of infection at maturity; ***very highly significant at p< 0.001, **highly significant at p< 0.001, *significant at p< 0.05, ns, non-significant at p= 0.05 significance level.

**Table 3:** Variance components, heritability, and genetic advances of coefficient of infection traits of 178 wheat genotypes based on pooled data from three environments.

Trait	σ^2^g	σ^2^e	σ^2^ge	PCV	GCV	H^2^	h^2^	GA	GAM
YRPH	85.28^***^	68.59^***^	6.49^***^	117.53	61.49	27.37	81.47	5.81	66.36
YRH	209.20^***^	156.36^***^	13.44^***^	78.41	45.36	33.46	84.01	11.44	54.12
YRF	274.82^***^	213.39^***^	13.44^***^	70.18	42.34	36.40	86.35	14.71	52.71
YRMM	303.67^***^	257.31^***^	8.79^***^	65.27	40.23	37.98	88.64	16.66	51.15
YRM	541.58^***^	273.88^***^	8.89^***^	78.65	43.12	30.05	74.54	14.97	48.76
Average				79.73	45.93	34.15	83.83	12.85	54.83

YRPH, coefficient of infection at pre-heading; YRH, coefficient of infection at heading; YRF, coefficient of infection at flowering; YRMM, coefficient of infection at mid-maturity; YRM, coefficient of infection at maturity; σ^2^g, Genotypic variance estimate; σ^2^p, phenotype variance estimate, σ^2^e, residual variance estimate; GCV, Genotypic coefficient of variance; PCV, Phenotypic coefficient of variance; GA, Genetic advance; GAM, Genetic advance as percent mean (GAM); ***very highly significant at p< 0.001, *significant at p< 0.05.

By using a comparative resistance analysis with the standard check King-bird (G40) and the average performance of the released varieties, it was demonstrated that among the investigated materials, there were superior genotypes with yellow rust resistance. Among the 178 genotypes examined, 106 (60.57%) genotypes at pre-heading, 38 (19.66%) genotypes at heading, 120 (67.42%) at flowering, 123 (60.16%) at mid-maturity, and 132 (74.16%) at maturity stage exhibited quantitatively superior yellow rust resistance as compared to the standard check King-bird (**Table 4**; **Supplementary Table 4**). The top 5% best genotypes had 82.21%-100% greater resistance at pre-heading, 55.27%-73.16% at heading, 57.26%-80.70% at flowering, 61.11%-72.65% at mid-maturity, and 61.34%-75.46% greater resistance at maturity stage compared to the mean performance of the King-bird. Furthermore, the top 5% of the best germplasms had 80.53%-100% greater resistance at pre-heading, 67.95%-80.77% at heading, 69%-86% at flowering, 47.70%-63.22% at mid-maturity and 71.70%-82.04% at maturity stage, according to the average performance of the released varieties (**Table 4**).

**Table 4:** Comparison of top 5% of the selected genotypes for Yr resistance with KB, and mean performances of 13 released varieties.

Genotypes	Mean of selected genotypes at YRPH	Comparative advantage for Yr resistance (% over)		Genotypes	Mean of selected genotypes at YRH	Comparative advantage for Yr resistance (% over)		
		KB	MRV*			KB		MRV*
G28	0.00	100.00	100.00	G171	5.00	73.16		80.77
G32	0.00	100.00	100.00	G176	5.00	73.16		80.77
G74	0.00	100.00	100.00	G93	6.00	67.79		76.92
G90	0.00	100.00	100.00	G125	6.17	66.90		76.28
G34	1.00	89.66	88.32	G90	7.00	62.43		73.08
G133	1.33	86.21	84.42	G21	8.00	57.06		69.23
G3	1.67	82.76	80.53	G34	8.00	57.06		69.23
G38	1.67	82.76	80.53	G33	8.17	56.16		68.59
G39	1.67	82.76	80.53	G58	8.33	55.27		67.95
KB	9.67	–	-12.97	KB	18.63	–		28.35
MRV*	8.56	11.44	–	MRV*	26.00	-39.56		–
Genotypes	Mean of selected genotypes at YRF	Comparative advantage for Yr resistance (% over)		Genotypes	Mean of selected genotypes at YRMM	Comparative advantage for Yr resistance (% over)		
		KB	MRV*			KB	MRV*	
G88	4.67	80.70	86.00	G93	10.67	72.65	63.22	
G93	6.00	75.19	82.00	G176	11.00	71.79	62.07	
G125	6.67	72.43	80.00	G33	13.00	66.67	55.17	
G176	8.00	66.91	76.00	G125	13.33	65.81	54.02	
G73	9.67	60.02	71.00	G38	14.00	64.10	51.72	
G159	9.67	60.02	71.00	G79	14.17	63.68	51.15	
G58	10.00	58.64	70.00	G81	14.67	62.39	49.43	
G147	10.17	57.95	69.50	G150	14.67	62.39	49.43	
G59	10.33	57.26	69.00	G147	15.17	61.11	47.70	
KB	24.18	–	27.45	KB	39.00	–	-34.48	
MRV*	33.33	-37.84	–	MRV*	29.00	25.64	–	
Genotypes	Mean of selected genotypes At YRM	Comparative advantage for Yr resistance (% over)						
		KB	MRV*					
G93	11.00	75.46	82.04					
G176	13.00	71.00	78.78					
G38	14.00	68.77	77.14					
G147	15.17	66.17	75.24					
G150	15.67	65.05	74.42					
G125	15.83	64.68	74.15					
G33	16.33	63.57	73.33					
G79	17.00	62.08	72.24					
G55	17.33	61.34	71.70					
KB	44.83	–	26.80					
MRV*	61.25	-36.63	–					

G, genotypes; Yr, yellow rust; KB, King-Bird; MRV*, Mean of 13 selected released varieties. Negative values for comparative advantage indicate less yellow rust resistance of the genotype. YRPH, coefficient of infection at pre-heading; YRH, coefficient of infection at heading; YRF, coefficient of infection at flowering; YRMM, coefficient of infection at mid-maturity; YRM, coefficient of infection at maturity.

Pearson’s correlation analysis of important agronomic traits revealed a strong negative association of yellow rust resistance with the assessed traits, except for days of 50% flowering and maturity date, where the associations were non-significant. Spike length showed a non-significant, weak negative correlation with all yellow rust resistance traits except at the pre-heading stage, where the association was strongly negative. All the yellow rust resistance traits showed a strong negative correlation with leaf area, plant height, thousand kernel weight, number of kernels per spike, spike weight, and grain yield. The yellow rust resistance traits showed a significant weak to moderate negative correlation with spike length and plant height. (**Table 5**).

**Table 5:** Correlation analysis among disease severity, field response, coefficient of infection, and other agronomic traits at different plant stages. .

DS Traits	FD	MD	LA	PH	SL	TKW	NKS	SW	GY
YRPH	0.20 ^**^	-0.05^ns^	-0.55^**^	-0.51^***^	-0.21^***^	-0.51^***^	-0.72^***^	-0.88^***^	-0.66^***^
YRH	0.20^***^	^-0.06 ns^	^-0.48***^	-0.51^**^	-0.14 ^ns^	-0.48^***^	-0.63^***^	-0.78^***^	-0.60^***^
YRF	0.18^*^	0.004 ^ns^	-0.45^***^	-0.50^***^	-0.07 ^ns^	-0.51^***^	-0.63^***^	-0.88^***^	-0.66^***^
YRMM	0.17^*^	0.001^ns^	-0.49^***^	-0.51^***^	-0.06 ^ns^	-0.47^***^	-0.63^***^	-0.81^***^	-0.65^***^
YRM	0.13 ^ns^	-0.02 ^ns^	-0.50^***^	-0.52^***^	-0.55 ^ns^	-0.47^***^	-0.64^***^	-0.81^***^	0.65^**^
FR traits	FD	MD	LA	PH	SL	TKW	NKS	SW	GY
YRPH	0.27 ^**^	-0.01^ns^	-0.39^**^	-0.34^***^	-0.14^**^	-0.48^***^	-0.61^***^	-0.78^**^	-0.50^***^
YRH	0.54^**^	0.15^*^	-0.55^***^	-0.02^ns^	-0.29^ns^	-0.81^***^	-0.62^***^	-0.82^***^	-1.07^***^
YRF	0.10^ns^	0.02^ns^	-0.44^***^	-0.34^**^	-0.05^ns^	-0.51^**^	-0.54^***^	-0.62^***^	-0.85^**^
YRMM	0.29^**^	0.16^*^	-0.55^***^	-0.42^***^	-0.10^***^	-0.67^***^	-0.67^***^	-0.82^***^	-1.02^***^
YRM	0.12^ns^	-0.15	-0.37^***^	-0.60^**^	-0.08^ns^	-0.75^***^	-0.70^***^	-0.72^***^	-0.77^***^
CI Traits	FD	MD	LA	PH	SL	TKW	NKS	SW	GY
YRPH	0.26 ^***^	-0.02 ^ns^	-0.55^**^	-0.48^***^	-0.04 ^ns^	0.80^***^	-0.73^***^	-0.89^***^	-0.68^***^
YRH	0.29^***^	-0.04 ^ns^	-0.48^**^	-0.49^***^	-0.12 ^ns^	-0.49^***^	-0.62^***^	-0.79^***^	-0.62^***^
YRF	0.23^***^	-0.01 ^ns^	-0.50^**^	-0.48^**^	-0.09 ^ns^	-0.51^**^	-0.65^***^	-0.80^***^	-0.67^***^
YRMM	0.23^***^	-0.01 ^ns^	-0.54^**^	-0.50^***^	-0.10 ^ns^	-0.50^***^	-0.67^***^	-0.83^***^	-0.71^***^
YRM	0.22^***^	-0.00 ^ns^	-0.49^**^	-0.48^**^	-0.09 ^ns^	-0.52^***^	-0.67^***^	-0.83^***^	0.68^***^

YRPH, coefficient of infection at pre-heading; YRH, coefficient of infection at heading; YRF, coefficient of infection at flowering; YRMM, coefficient of infection at mid-maturity; YRM, coefficient of infection at maturity; FD, flowering date; MD, maturity date; LA, leaf area; TKA, thousand kernel weight; NKS, number of kernels per spike; SW, spike weight; GY, grain yield; ***, very highly significant at p< 0.001, **highly significant at p< 0.001, *significant at p< 0.05, ns, non-significant at p= 0.05 significance level.

### 3.2 SNP statistics

**Figure 5:**
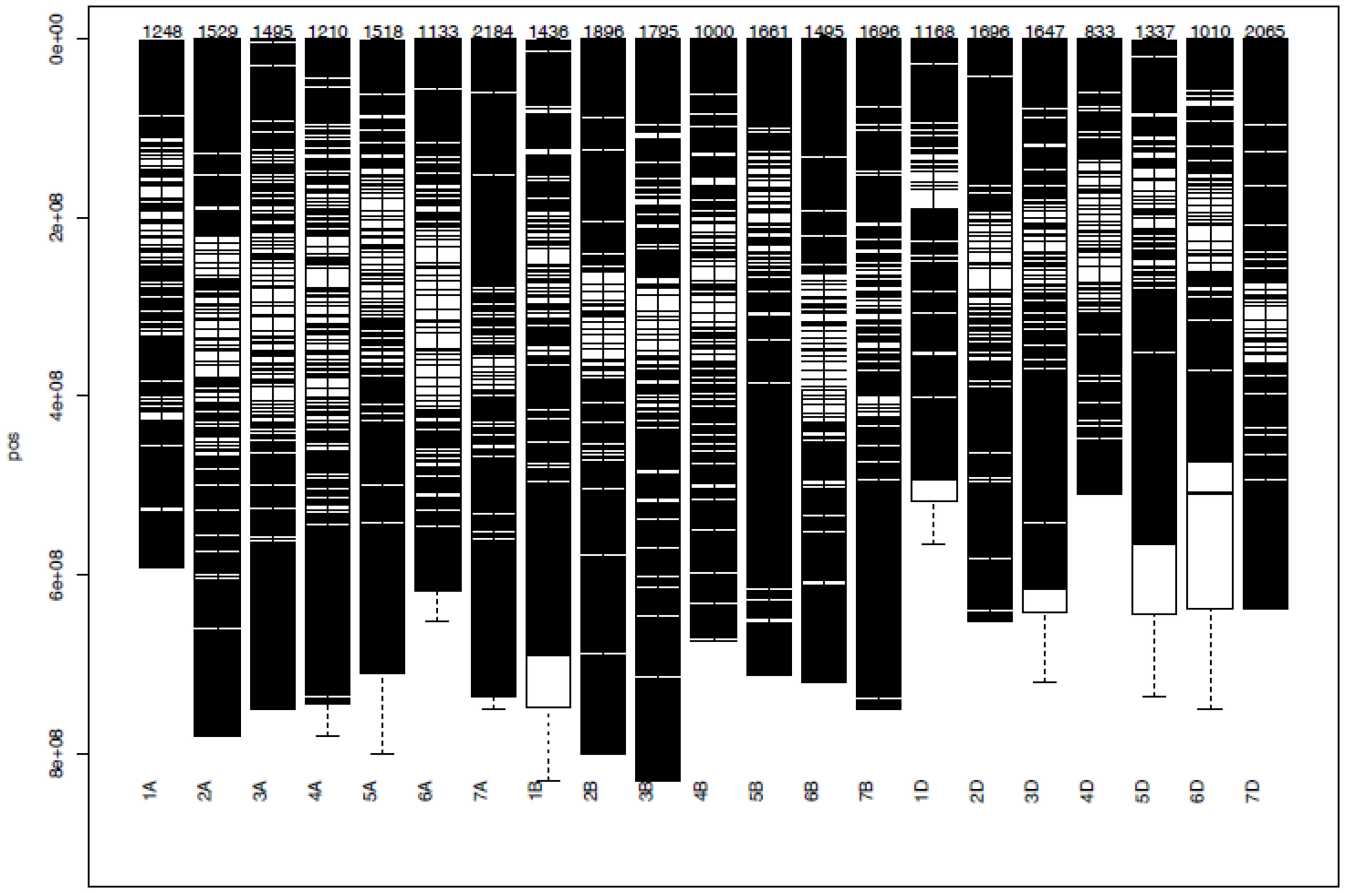
Distribution of DArTSeq SNPs on 21 bread wheat chromosomes.

DArTSeq genotyping of the 178 bread wheat germplasms resulted in a total of 35,672 SNPs (**Figure 5**). Among the three wheat sub-genomes (A, B, and D), 10,317, 10,979, and 9,756 SNPs were distributed, respectively (**Figure 5**). Among the 21 wheat chromosomes, chromosome 4D contained the lowest (833) number of SNPs, while the highest (2065) number of SNPs was found on chromosome 7D. After filtering with call rates > 70% and minor allele frequencies > 0.05, a total of 6,788 SNP markers were obtained that were distributed as 2,410 SNPs on the A sub-genome and 2,872 on the B sub-genome, and the filtered 6,788 SNPs were used in successive analyses.

### 3.3 Population structure analysis

**Figure 6:**
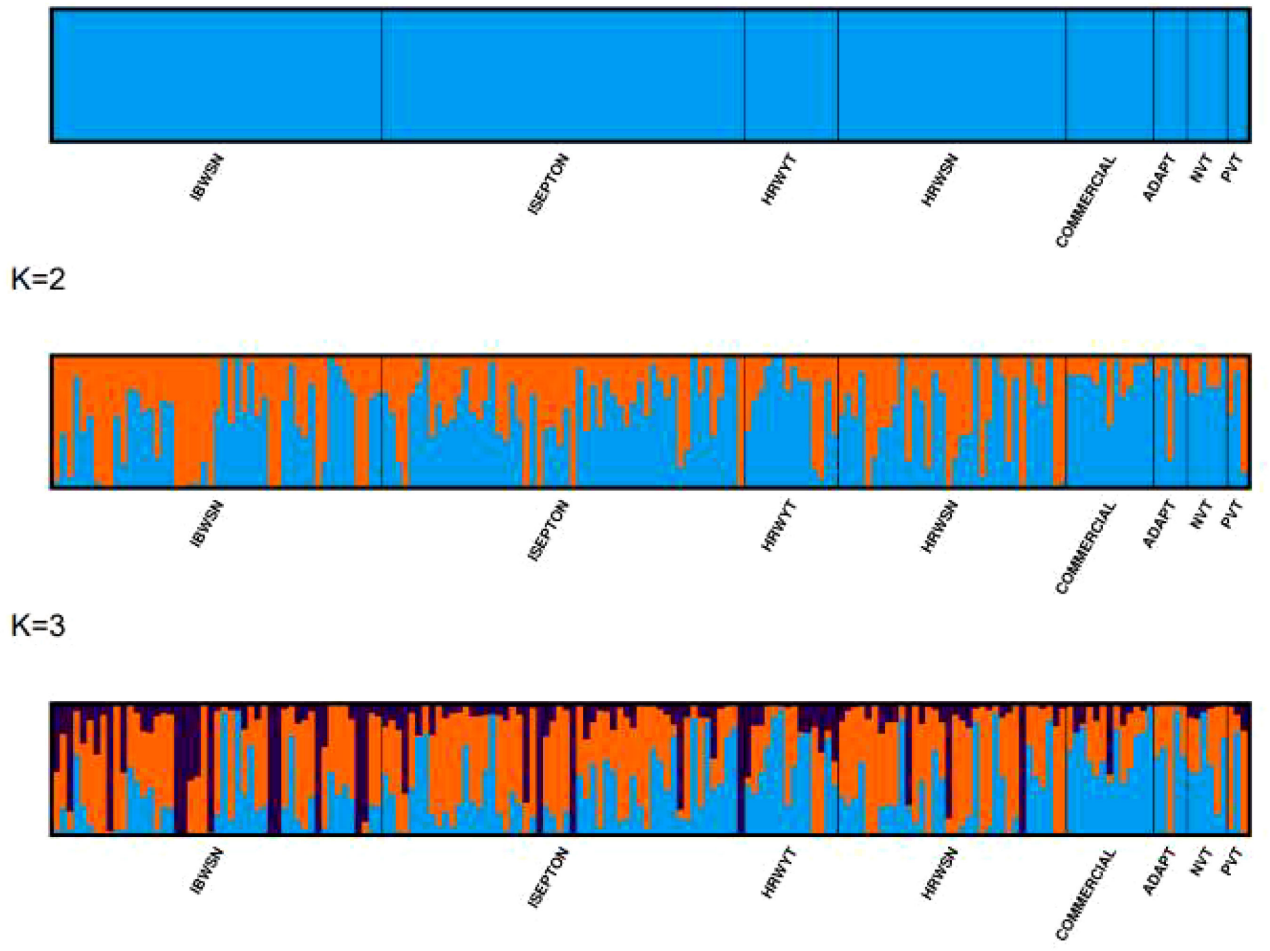
Population structures of 178 bread wheat genotypes representing eight populations. **(A)** Best delta K value estimated, and the pick at k = 3 indicates the number of sub-populations in the wheat panel. **(B)** Estimated population structure for K = 3 according to the breeding materials. The different colors (blue, orange, and black) represent genetic groups or sub-populations: the x-axis represents individual samples and the y-axis represents the proportion of ancestry to each cluster. Population abbreviations are IBWSN, International Bread Wheat Screening Nursery; ISEPTON, International Septoria Observation Nursery; HRWYT, High Rain Wheat Yield Trial; HRWSN, High Rain Wheat Screening Nursery; ADAPT, Adaptation trial; NVT, National Verification Trial; and PVT, Preliminary Verification trial.

**Figure 7:**
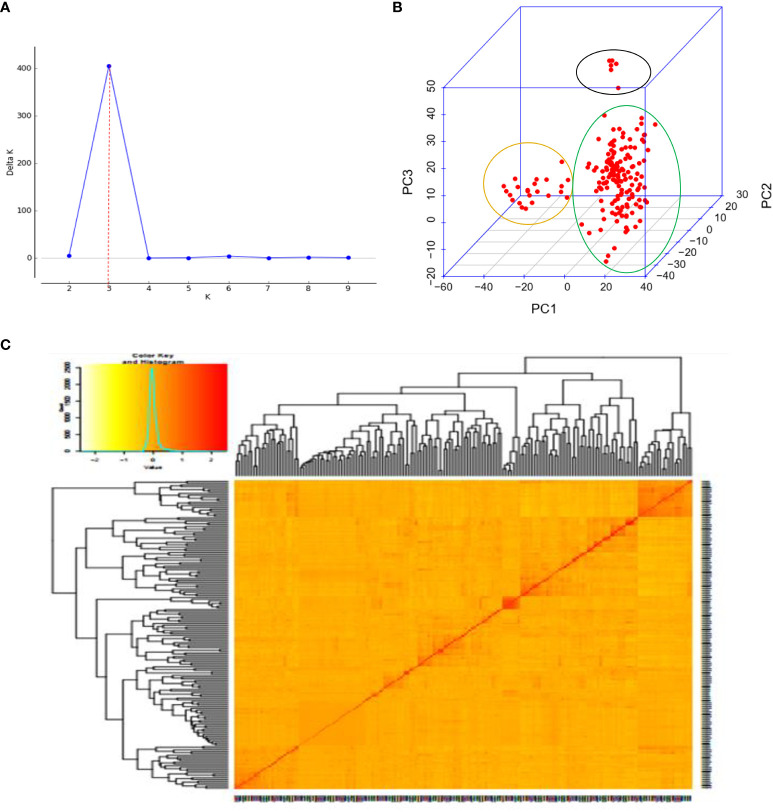
Principal component and familiar relatedness analysis of 178 wheat genotypes using 6,788 SNP markers. **(A)** Scatter plot and **(B)** 3D plots of the principal components. **(C)** Kinship displayed through a heat map and a tree out of the heat map. The kinship values showed a normal distribution (turquoise curve), and orange represents a weak correlation between pairs of individuals in the panel while red shows a high correlation. The resulting clustering tree is indicated outside of the matrix.

Three sub-populations were inferred based on the output of the STRUCTURE software (**Figure 6A**). The three clusters (**Figure 6B**) showed a significant degree of genetic mixing, indicating that the studied wheat populations are closely related. All of the individual genotypes shared genes inherited from all three subpopulations. The scatter plot (**Figure 7A**) and 3D plot of the first three principal components (**Figure 7B**) also confirmed the existence of three clusters in the association with greater admixture, where the first two PCs (PC1 and PC2) coordinates explained the majority of the variation in the association panel. The presence of cryptic familiar relatedness was also verified by kinship analysis (**Figure 7C**), demonstrating the significance of including both population structure (Q) and kinship (K) as covariates in marker-trait association analyses.

### 3.4 Linkage disequilibrium analysis

**Figure 8:**
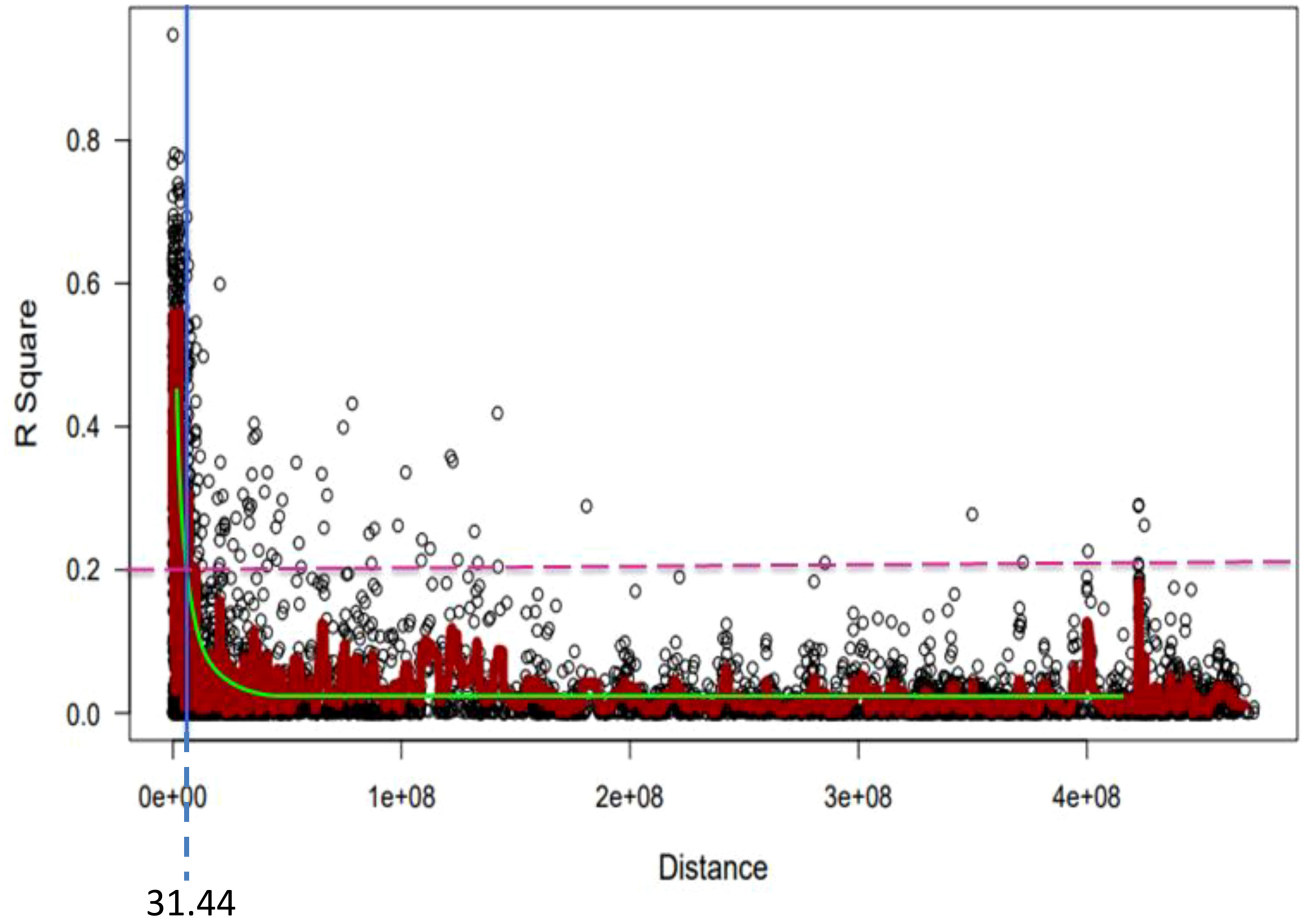
Genome-wide LD decay plot over physical distance based on 6,788 SNP markers. The yellow curve represents the model fits to LD decay. The horizontal magenta dash-line represents the arbitrary threshold for no LD (r^2 ^= 0.2). The vertical blue line indicates the intersection between the critical r^2^ value and the average map distance (31.44 bp) to determine QTL confidence intervals.

For the 6,788 SNP markers across 178 genotypes, LD was calculated. LDs’ alleles differ between chromosomes and within sub-genomes (**Table 6**). The scatter plot of the genome-wised pairwise LD decay plot is shown in **Figure 8**. Overall, 97,723 (27.61%) of the 338,125 marker pairings with average LD values of r^2 ^= 0.11 showed significant *p* ≤ 0.01 LD. The B sub-genome had the highest number of (143,600, or 42.47%) marker pairs, while the D sub-genome had the fewest (75,300, or 22.27%) marker pairs. Comparatively, SNPs on the B sub-genome showed the greatest LD with a mean value of r^2 ^= 0.1187. The LD between SNPs declined across all chromosomes at the LD cut-off r^2 ^= 0.2 within a physical distance of 31.44 Mbp. The marker pairs on chromosome 4D and 2D showed the poorest (r^2 ^= 0.03, and strongest r^2 ^= 0.21) (**Table 6**.

**Table 6:** Summary of LD analysis among marker pairs per chromosome and sub-genomes.

Chr	Total marker pairs	r^2^	Distance (Mbp)	Significant marker pairs (*P*<0.01)	Chr	Total marker pairs	r^2^	Distance (Mbp)	Significant marker pairs (*P*<0.01)
1A	12,475	0.11	58.05	5,032 (35.06)	5B	22,750	0.14	40.22	8,193 (36.01)
1B	19,900	0.10	44.93	5,820 (29.8)	5D	11,100	0.12	59.51	2,273 (20.48)
1D	10,250	0.13	72.36	1,783 (17.40)	6A	14,700	0.08	57.51	3,928 (26.72)
2A	21,200	0.15	50.03	7,342 (34.63)	6B	18,800	0.11	50.53	6,163 (32.78)
2B	28,100	0.11	36.88	9,511 (33.85)	6D	7,950	0.05	87.75	911 (11.46)
2D	14,550	0.21	57.45	4,771 (32.79)	7A	22,750	0.09	42.53	6,382 (28.05)
3A	17,450	0.09	57.38	4,259 (24.41)	7B	22,450	0.11	42.47	7,492 (33.37)
3B	22,000	0.12	49.85	6,795 (30.89)	7D	14,100	0.08	60.62	2,246 (15.93)
3D	14,650	0.12	56.41	3,940 (26.90)	A sub-genome	119,225	0.11	56.04	35,012 (29.44)
4A	13,050	0.11	75.78	3,830 (29.35)	B sub-genome	143,600	0.12	51.63	47,527 (33.24)
4B	9,600	0.14	96.54	3,463 (36.08)	D sub-genome	75,300	0.11	65.7	15,184 (20.16)
4D	2,700	0.03	218.8	1,71 (6.34)	Total	338,125	0.11	57.79	97,723 (27.61)
5A	17,600	0.09	52.97	4,897 (27.83)					

### 3.5 Genome-wide association study

Linkage-disequilibrium and Bayesian information were used in the MTA analysis. At a nominal *p-value* of 0.001, or –log10 (0.001) =3 the BLINK statistical model-based association analysis discovered 148 SNPs significantly associated with the coefficient of infection. **Supplementary Table 4** reports MTAs that exceeded the nominal *p-value* of 0.001 or -log10 (0.001) = 3 significance thresholds for yellow rust resistance at pre-heading, heading, flowering, mid-maturity, and maturity growth stages. In addition, allele identity, marker position, *p*-values, additive effects, and r^2^ of the identified MTAs were computed. Among 148 identified MTAs, 17 (11.49%) MTAs conferred yellow rust resistance at pre-heading, 24 (16.22%) at heading, 34 (24.32%) at flowering, 21 (18.24%) at mid-maturity, 25 (16.89%) at the level of maturity, and 28 (18.92%) MTAs for all stages were effective. The percentage of phenotypic variation explained by the markers varied significantly from 1.3% for yellow rust resistance measured at heading at Holeta to 14.14% for the resistance measured in the pre-heading stage at Meraro.

At the pre-heading stage, the proportion of phenotypic variation (r^2^) explained by the detected significant markers extended from 2.19% for the SNP 1214020|F|0-68:A>C-68:A>C on chromosomes 1D to 14.14% for the SNP 5969589|F|0-49:C>G-49:C>G on 1D. Likewise, the proportion of phenotypic variation (r^2^) explained by the significant SNP markers at the heading stage ranged from 1.3% for 3020463|F|0-7:A>G-7:A>G and 1000215|F|0-22:G>T-22:G>T A on chromosomes 1A to 11.09% for 4910323|F|0-27:C>A-27:C>A on 1D. Similarly, the r^2^ for MTAs for yellow rust resistance at flowering ranged from 9.74 - 14.05% for the allele 1062638|F|0-63:G>A-63:G>A and 1000215|F|0-22:G>T-22:G>T A on chromosomes 1A and 2A respectively. The r^2^ of MTAs for yellow rust resistance at mid-maturity ranged from 8.71 - 13.63%, for the SNP markers 1115117|F|0-21:T>C-21:T>C and 1094246|F|0-48:A>G-48:A>G on chromosomes 1A and 2B respectively. Moreover, the phenotypic variance explained by the MTAs at maturity for yellow rust resistance ranged from 7.1% for the SNP 1245237|F|0-24:G>A-24:G>A on chromosomes 2A to 14.05% for the SNP marker 2264044|F|0-68:T>C-68:T>C on chromosomes 3B. Furthermore, the proportion of yellow rust resistance explained by the significant alleles at the maturity stage ranged from 7.94% for the allele whereas the allele 4008773|F|0-17:G>A-17:G>A on chromosomes 2B to 13.63% for the SNP 1094246|F|0-48:A>G-48:A>G on chromosomes (**Supplementary Table 4**).

**Figure 9:**
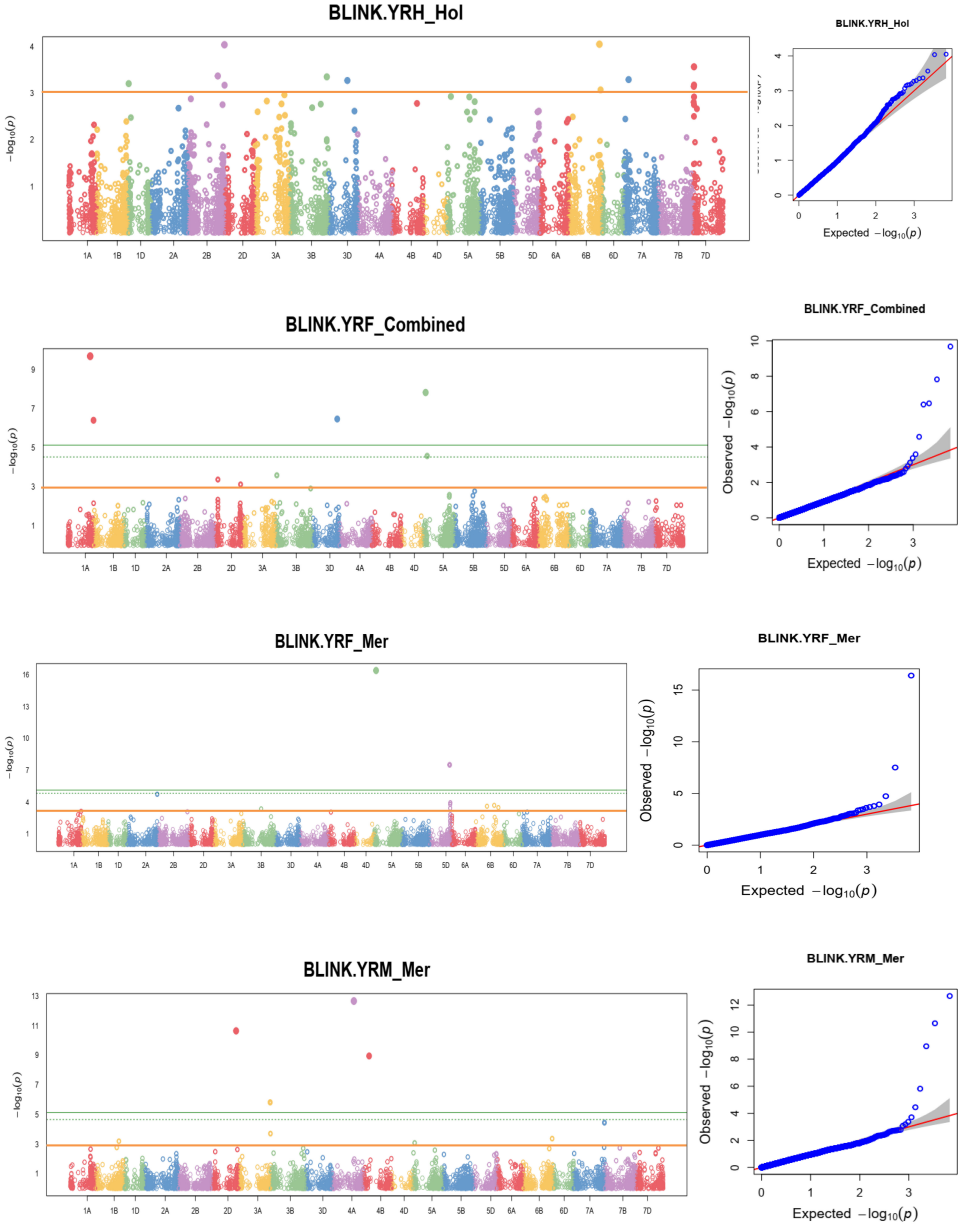
Some examples of Manhattan plots for the coefficient of infection and GWAS scans resulting in significant associations. Each dot represents an SNP. On the x-axis is the genomic position of the SNPs on the corresponding chromosomes indicated in different colors. On the y-axis is the *-log10* of the *p-value* depicting the significance of the association test. The horizontal orange line is the nominal *p-*value 0.001 significance threshold used in the association analysis for YRPH, coefficient of infection at pre-heading; YRH, coefficient of infection at heading; YRF, coefficient of infection at flowering; YRMM, coefficient of infection at mid-maturity; and YRM, coefficient of infection at maturity. The quantile-quantile (Q-Q) plots at the right side of the Manhattan plots indicate how well the used BLINK model accounted for population structure and kinship for each of the disease traits. In each plot, the observed *–log (P values)* from the fitted GWAS models (y-axis) are compared with their expected value (x-axis) under the null hypothesis of no association with the trait. Each blue dot represents a single nucleotide polymorphism; the red line is the model for no association.

A genome-wide scan for the coefficient of infection in individual environments identified significant markers associated with yellow rust resistance at different locations. For instance, MTA analysis at Holeta identified six MTAs significantly associated with yellow rust resistance at the pre-heading stage on chromosomes 1D, 2B, 3A, 5A, 3D, 4A, and 6B; 12 MTAs at the heading stage on chromosomes 1D, 2B, 3B, 6B, 7A, and 7D; seven significant MTAs conferring coefficient of infection at the flowering stage on chromosomes 2A, 2B, 3A, 5A, 7A, and 7D; nine MTAs at the mid-maturity stage on chromosomes 2B, 3A, 5A, 7A, and 7D; eight significant MTAs at the maturity stage on chromosomes 2B, 3A, and 6A; and seven MTAs for yellow rust resistance across all stages on chromosomes 2B, 3A, 5A, 7A, and 7D. The analysis revealed that MTAs on chromosome 2B conferred stable yellow rust resistance across all growth stages at Holeta. Likewise, a GWA scan for yellow rust resistance at Kulumsa identified 30 MTAs conferring resistance to the coefficient of infection at various growth stages: two MTAs at the pre-heading stage on chromosome 1B; seven MTAs at the heading stage on chromosomes 1D, 4D, 6B, and 7A; four MTAs at the flowering stage on chromosomes 1A, 1B, 1D and 7A; six MTAs at the mid-maturity stage on chromosomes 1A, 1B, 1D, 6B and 7A; five MTAs at the maturity stage on chromosomes 1D, 1B and 7A; and six MTAs for yellow rust resistance across all stages on chromosomes 1A, 1B, 1D, 6B and 7A. Moreover, a GWA scan for the yellow rust resistance analysis at Meraro discovered numerous (46) MTAs that confer resistance to yellow rust at various growth stages. The analysis identified four MTAs at the pre-heading stage on chromosomes 1A, 1D, 2B, and 5B; three MTAs at the heading stage on chromosomes 2B, 3A, and 7D; 15 MTAs at the flowering stage on chromosomes 1A, 2A, 2B, 3B, 4B, 5A, 5D, 6B, and 7A; five MTAs at the mid-maturity stage on chromosomes 2B, 3A, 4B and 6B; eight MTAs at the maturity stage on chromosomes 2D, 3A, 4A, 4B, 5A, 6B and 7A; and 11 MTAs for yellow rust resistance at all stages of growth on chromosomes 1A, 1B, 2D, 3A, 3B, 3D,4B, and 5A. The combined measure of yellow rust resistance across locations at the heading, mid-maturity, and maturity stages provided significant associations. It found 20 MTAs, including one at mid-maturity on chromosomes 1A, two at the maturity stage on chromosomes 1A and 1B, and two for yellow rust resistance across locations and all stages on chromosomes 3A and 3B (**Supplementary Table 4**; **Figure 9**; **Supplementary Figure 1**). Eight MTAs were found at the flowering stage on chromosomes 1A, 2D, 3D, 5A, and 7A; one MTA at the mid-maturity stage on chromosomes 1A; two MTAs at the maturity stage on chromosomes 1A and 1B; and two MTAs for yellow rust resistance across locations and all stages on chromosomes 3A and 3B. It was observed that MTAs on chromosome 7A were detected to contribute to stable yellow rust resistance across all environments. The allele contributes to yellow rust resistance at the flowering stage across all environments. Moreover, the allele on chromosome 2B is also found to confer yellow rust resistance at Holeta and Meraro.

**Figure 10:**
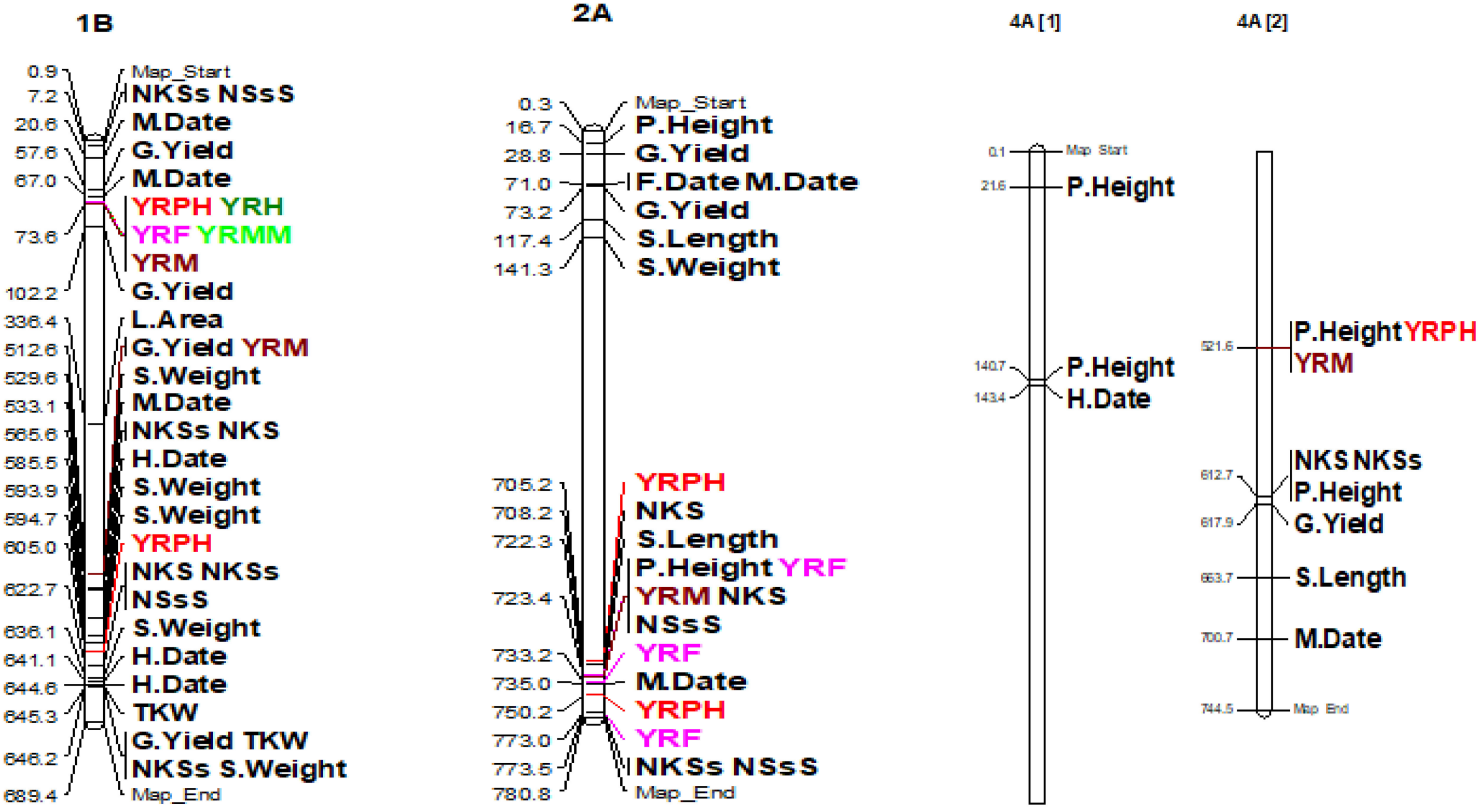
Some examples of genomic positions of detected putative QTLs effective for the coefficient of infection. Significant DArTSeq SNPs are presented according to their physical positions on chromosomes in million base pairs. The putative QTLs identified in this study for the MTAs are indicated on the right side of the bars. YRPH, coefficient of infection at pre-heading; YRH, coefficient of infection at heading; YRF, coefficient of infection at flowering; YRMM, coefficient of infection at mid-maturity; YRM, coefficient of infection at maturity; HD, days to heading; FD, days to flowering; DM, days to maturity; TKW, thousand kernel weight; LA, leaf area; PH, plant height; SL, spike length; NSs/S, number of spikelets per spike; NK/S, number of kernels per spike; NK/Ss, number of kernels per spikelets; SW, spike weight; and GYPP, grain yield per plot.

The coefficient of infection putative QTLs were discovered by combining the MTAs based on their physical distance in Mbp. MTAs on the same linkage group within the physical distance for LD decay specific to that chromosome were assigned to the same putative QTL (**Supplementary Table 5**; **Figure 10**; **Supplementary Figure 2**). Accordingly, the 148 MTA markers were assigned to 54 putative coefficients of infection QTLs based on our LD criteria. The association analysis for yellow rust resistance at pre-heading in individual environments found 12 putative QTLs that were on chromosomes 1A (qYrS.02), 1B (qYrS.06 and qYrS.08), 1D (qYrS.09, qYrS.10, and qYrS.11), 2A (qYrS.12), 2B (qYrS.17 and qYrS18). It was observed that some of the detected putative QTLs were effective for the coefficient of infection at several growth stages while others contributed to the coefficient of infection at a particular growth stage (eg., qYrS.56 contributed to yellow rust resistance at the maturity stage). For instance, qYrS.10 contributed to a stable coefficient of infection at the pre-heading, heading, mid-maturity, and maturity stages. Similarly, qYrS.28 was found to be effective for yellow rust resistance at the heading, flowering, mid-maturity, and maturity stages. qYrS.02 also contributed to the coefficient of infection at the pre-heading, mid-maturity, and maturity stages. qYrS.54 was effective for yellow rust resistance at the heading, flowering, and maturity stages. Likewise, qYrS.13 contributed to resistance in later stages such as the mid-maturity, and maturity stages. Putative QTLs that contributed to stable yellow rust resistance from the flowering to maturity stages include qYrS.20, qYrS.27, qYrS.28, and qYrS.43. **Supplementary Table 5** presents more information on the detected putative QTLs.

By annotating genes detected in the QTL regions using the recently released IWGSC RefSeq Annotation v2.1, the functional relationship between the identified QTLs and yellow rust resistance was further studied. Annotation identified several resistance-associated genes that are involved in the plant defense system (**Supplementary Table 7**). Some of the identified high-confidence candidate genes involved in defense response to fungus include *TraesCS1A02G325400* on chromosome 1A, *TraesCS1B02G067000* on 1B, *TraesCS1D02G018500* on 1D, *TraesCS2B02G548900* on 2B, *TraesCS2D02G497400* on 2D, *TraesCS3A02G395300*, on 3A, *TraesCS3B02G041900* on 3B, *TraesCS3D02G461200* on 3D, *TraesCS4A02G312500* on 4A, *TraesCS4B02G074400*, on 4B, *TraesCS4D02G286100* on 4D, *TraesCS5D02G483900* on 5D, TraesCS6A02G083200 on 6A, *TraesCS6B02G328600* on 6B, and *TraesCS7A02G028300* on 7A. Furthermore, regarding high-confidence candidate genes important for systemic acquired resistance (SAR), it has been revealed that wheat has a long-lasting, broad-spectrum resistance to pathogen infections. These include *TraesCS1A02G18340* on 1A, *TraesCS1B02G324300* and *TraesCS1B02G480300* on 1B, *TraesCS1D02G018500* on 1D, *TraesCS2A02G540000* on 2A, *TraesCS2B02G533800*, *TraesCS2B02G535000* and *TraesCS2B02G583600* on 2B, *TraesCS3B02G041900* on 3A, *TraesCS5D02G480600*, *TraesCS5D02G501100* and *TraesCS5D02G501800* on 5D, *TraesCS7A02G026100* and *TraesCS7A02G175200* on 7A, and *TraesCS7D02G016800* on 7D. Focusing on the significant QTL regions identified that high-confidence genes *TraesCS1B02G324300* on 1B, *TraesCS6B02G017900* on 6B, *TraesCS7A02G175200* on 7A and *TraesCS7D02G016800* on 7D regulate mitogen-activated protein kinase (MAPK) cascades, which are involved in signaling a variety of plant defense responses against pathogen infections (Meng and Zhang, 2013) (**Supplementary Table 6**).

### 3.6 MTAs for Agronomic Traits

**Figure 11:**
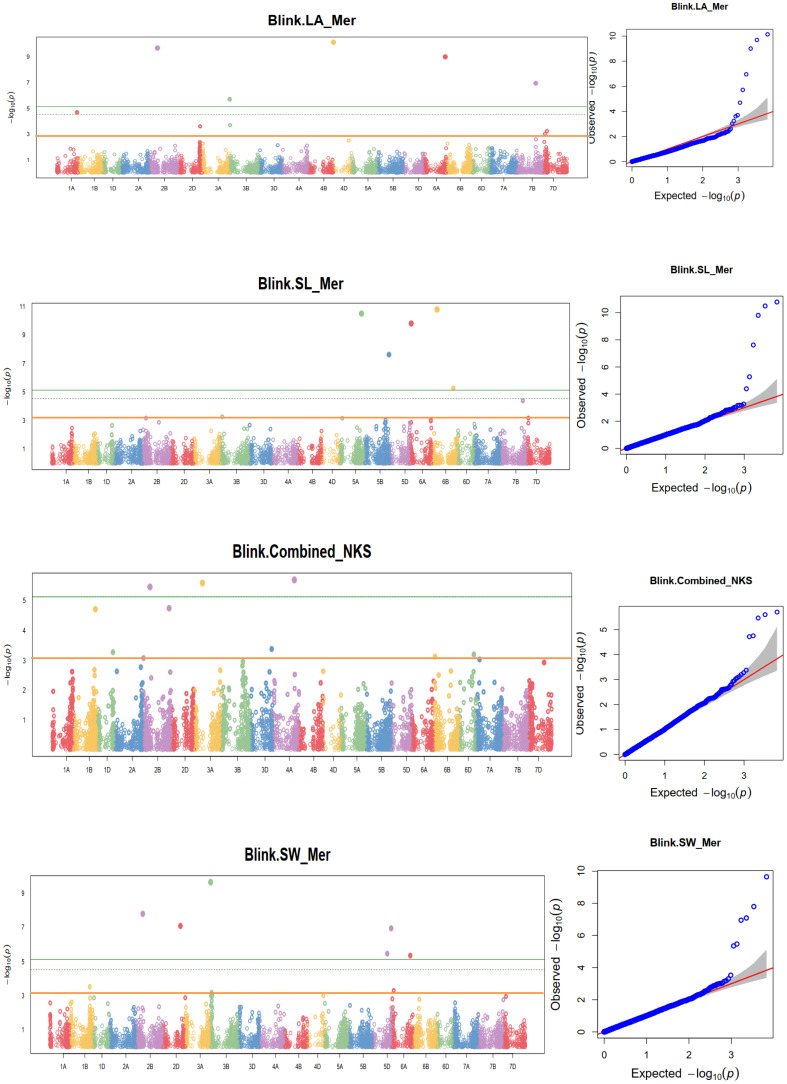
Some examples of Manhattan plots for agronomic and yield-related traits in each environment and combined data. HD, days to heading; FD, days to flowering; DM, days to maturity; TKW, thousand kernel weight; LA, leaf area; PH, plant height; SL, spike length; NSs/S, number of spikelets per spike; NK/S, number of kernels per spike; NK/Ss, number of kernels per spikelets; SW, spike weight; and GYPP, grain yield per plot. The quantile-quantile plots at the right side of the Manhattan plots indicate how well the GWAS model accounted for population structure and kinship for each of the disease traits. In each plot, the observed *–log (p-values)* from the fitted GWAS models (y-axis) are compared with their expected value (x-axis) under the null hypothesis of no association with the trait. Each blue dot represents a single nucleotide polymorphism; the orange line is the model for no association.

The association analysis for some agronomic traits, including FD, LA, PH, SL, NKS, and SW, resulted in significant MTAs at the nominal *p-value*. For instance, a GWA scan for days to 50% flowering for pooled dates resulted in seven MTAs on chromosomes 2B, 2D, 3A, 4B, 6B, 7A, and 7D. However, dissecting the traits in individual environments detected considerable MTAs at the nominal significance threshold. The same trait provided six MTAs that were produced by days to flowering on chromosomes 2D, 3A, 4B, 5A, 6B, and 6D. The identified SNPs explained 11.66% (1123012|F|0-16:G>C-16:G>C on 6B) to 14.70% (1090345|F|0-8:T>A-8:T>A on 6B) of the total variations in days to flowering. Likewise, the association analysis for leaf area identified six MTAs on chromosomes 1A, 2B, 3B, 4D, 6A, and 7B at Holeta; seven MTAs at Kulumsa on chromosomes 1B, 3A, 5D, 6A, 6B, and 7A; and eight MTAs at Meraro on chromosomes 1A, 2B, 2D, 3B, 4D, 6A, 7B, and 7D. The identified SNPs explained 10.04-17.29% of the total variation in leaf area for the SNP markers 1119123|F|0-11:C>G-11:C>G on chromosome 1D and 1265768|F|0-44:T>C-44:T>C on chromosome 4D, respectively. The association analysis for plant height data collected at Meraro identified five MTAs pointing to chromosomes 1D, 2B, 4A, 5D, and 7B. The identified SNPs explained 6.61% (3025953|F|0-25:G>A-25:G>A on 4A) to 12.30% (SNP 1099369|F|0-26:C>T-26:C>Ton 5D) of the total phenotypic variance for plant height (**Supplementary Table 7**; **Figure 11**; **Supplementary Figure 3**).

The association analysis for pooled spike length identified five MTAs on chromosomes 1A, 2A, 5B, 6B, and 7A. Analysis of the pooled data for days to maturity identified significant SNPs on chromosomes 1B, 2A, 2B, 3A, 5B, and 5D. The association analysis for days to maturity in individual environments identified 9 MTAs at Holeta on chromosomes 1A, 1B, 3B, 5A, and 5B; nine MTAs on chromosomes 1B, 1D, 3A, 3B, 4B, 5B, and 7A for data collected at Kulumsa; and eight MTAs on chromosomes 1B, 2A, 2B, 4A, 5D, 6A and 6B for days of maturity collected at Meraro (**Supplementary Table 5**). The identified SNPs explained 5.54% to 8.04% of the total variations in days to maturity for the SNPs 1104531|F|0-39:T>A-39:T>A on 2B and 4911245|F|0-46:A>G-46:A>G on 6B, respectively. Association analysis for the pooled number of kernels per spike identified eight MTAs on chromosomes 1B, 1D, 2B, 3A, 3D, 4A, 6B, and 6D. Analyses of the same trait at Meraro provided five MTAs pointing to chromosomes 1D, 2B, 4A, 5D, and 7B. The identified SNPs explained 6.62% of 3025953|F|0-25:G>A-25:G>A on 4A to 13.64% (for 992022|F|0-9:G>A-9:G>A) of the total variations of days to maturity (**Supplementary Table 7**; **Figure 11**; **Supplementary Figure 3**). GWAS for cumulative number of kernels per spikelets identified seven MTAs pointing to the chromosomes 1B, 3B, 4A, 6D, 7A, 7B, and 7D. The same trait analysis at each individual environment revealed 20 MTAs: six at Holeta on chromosomes 1B, 2B, 3B, 7A, 7B, and 7D; seven at Kulumsa on chromosomes 1B, 2A, 3D, 5A, 5D, 6A, and 7B; and seven at Meraro on chromosomes 1B, 2B, 2D, 3A, 4A, 7A, and 7D. The obtained SNPs explained 6.75-19.15% of the total phenotypic variations in the number of kernels per spikelets by the SNPs 1081747|F|0-27:T>C-27:T>C on 1B respectively.

The association analysis for spike weight found four MTAs on chromosomes 2D, 3A, 3B, and 5B at Kulumsa, and seven MTAs were found on chromosomes 1B, 2B, 2D, 3B, 4D, 5D, and 6A at Meraro. Similar to this, the association analysis for kernels per spike at Meraro identified five MTAs pointing to chromosomes 1D, 2B, 4A, 5D, and 7B. The identified SNPs explained 7.50-11.06% of the total variations in kernels per spike for the SNPs 1000905|F|0-46: A>G-46:A>G on 3B and 1126316|F|0-23:A>G-23:A>G on 2B, respectively (**Supplementary Table 7**; **Figure 11**; **Supplementary Figure 3**). The study showed that certain QTLs discovered for yellow rust resistance overlapped with QTLs for agronomic traits. For instance, the qYrS.12 gene, which was found to be associated with yellow rust resistance at the flowering stage, is co-mapped with the putative QTL found for plant height on chromosome 2A. Similarly, the putative QTL for grain yield discovered on 1B is also co-mapped with the yellow rust resistance qYrS.07 identified at the maturity stage (**Figure 10**).

## 4 Discussion

### 4.1 Phenotypic variability in resistance to *Puccinia striiformis* f. sp *tritici*

The average disease severity and field response of 178 bread wheat genotypes to *P. striiformis* f. sp. *tritici* at Holeta, Kulumsa, and Meraro were recorded in the field. The mean DS, FR, and CI of the tested wheat genotypes at Holeta, Kulumsa, and Meraro were 8.35, 26.92, and 45.08 for DS; 0.22, 0.73, and 8.30 for FR; and 27.55, 45.05, and 47.16 for CI, respectively. Meraro showed the highest DS, FR, and CI, confirming that it is a national hotspot for *P. striiformis* infestation. It is one of the national and international screening sites of international enter for maize and wheat improvement (CIMMYT) wheat germplasm for yellow rust resistance. The analysis revealed the existence of significant genetic variation among the wheat genotypes examined for a coefficient of infection, suggesting that yellow resistance could be improved through selection breeding. The coefficient of infection for resistance reaction detected in this research (69.66%) is comparable with the reaction response reported by Khalil et al. (2022) (77%) for 426 Indian bread wheat materials. All the considered traits showed moderate heritability (H^2 ^= 30 – 60%) except for the coefficient of infection at the pre-heading stage which showed low heritability (H^2 ^= 27%). Narrow-sense heritability, which estimates the proportion of phenotypic variations in yellow rust resistance in a wheat panel, is due to the additive genetic effect and was high (h^2 ^= 60%), indicating the presence of a strong genetic signal in the data and hence the promising possibility of improving yellow rust resistance by exploiting the resistance source through selection breeding. Comparable high heritability (h^2 ^= 88%) for yellow rust resistance traits was reported by (Zegeye et al., 2014). Similarly, Li et al. (2020) described high heritability (h^2 ^= 81%) for yellow rust resistance analysis in Chinese endemic wheat genotypes.

Correlation analyses using CI revealed that yellow rust resistance traits were significantly negatively associated with leaf area, plant height, thousand kernel weight, number of kernels per spike, spike weight, and grain yield, indicating that high yellow rust infestations have a considerable effect on reducing yield and yield-related traits. Moreover, plant height has a considerable escaping effect from yellow rust, as the infestation can initiate from the soil. Similarly, a significant negative association between yellow rust infestation and plant height, thousand kernel weight, grain yield, and number of kernels per spike was reported by Solomon (2022) and Saleem et al. (2022). Nevertheless, days to maturity and spike length showed a moderate negative correlation (r< -0.3) with all yellow rust severity, indicating that late maturing and long spiked genotypes have an escaping mechanism for yellow rust infection. The strong negative associations of yield-related, vegetative, and phenological traits with yellow rust resistance traits indicate that these traits should be considered in indirect selection for yellow rust disease resistance.

### 4.2 Population structure, relatedness, and LD

The presence of three sub-populations (K=3) with substantial mixing was supported by analysis of the population structure and principal components. Ovenden et al. (2017) showed similar indistinct population grouping, increased admixing, and poor population sub-structuring for 312 wheat genotypes using 5k SNP markers. The existence of cryptic familiar relatedness was also verified by kinship analysis, highlighting the significance of using both population structure (Q) and kinship (K) as covariates in marker-trait association analyses. The population stratification, relatedness, and marker effects have been effectively accounted for using the BLINK model employed in the association studies, reducing the confounding effects that could lead to false-positive MTAs. Visualizing the Q-Q plots helped to confirm effective control of the confounding factors.

According to the study, there is a relatively even distribution of markers among the bread wheat sub-genomes, with the A sub-genome contributing the most (10,317), the B sub-genome coming in second (10,979), and the D sub-genome harboring the least (9,756). Similar findings were published by Vikas et al. (2022), who found that 6,036 of the 18,932 mapped SNPs were mapped to the A sub-genome, 7,191 to the B sub-genome, and 5,705 to the D sub-genome. This indicates that the history of natural selection, gene conversion, mutation, and other mechanisms that result in gene-frequency evolution is reflected in each genomic region’s LD (Slatkin, 2008; Rahimi et al., 2019). The A and B sub-genomes had a disproportionately high number of SNPs, which was probably caused by the relatively recent joining of the D-genome to the hexaploid wheat genome. The LD varied amongst the three sub-genomes, and it was low for the A and B sub-genomes, perhaps as a result of their lengthy evolutionary histories in comparison to the D genome.

### 4.3 Marker-trait associations and identification of candidate genes

The discovery of significant marker-trait relationships at different growth stages revealed the presence of significant genotypic diversity among the investigated bread wheat genotypes. At a nominally significant threshold of *p-value* ≤ 0.001, GWAS analysis discovered 148 MTAs, pointing to 54 QTLs for the coefficient of infection and 179 MTAs for agronomic traits. (Zegeye et al., 2014) also found 38 SNPs in synthetic hexaploid wheat evaluated at Meraro and Arsi Robe. The large sample size and SNPs employed in the current investigation could be the main cause for the considerable variation in the number of significant markers. Among the 18 responsible reported by (Zegeye et al., 2014) for yellow rust resistance, four (1A, 1B, 2B, and 5A) shared the same physical position, which could be due to the difference in the number of used SNPs (2590), genotyping platform (iSelect array), and wheat populations.

In the current study, 48 of the identified yellow rust resistance SNPs were found to be location-specific (at Meraro), showing the presence of *P. striiformis* races at the test site that was distinct from other test sites, along with race-specific resistance genes. The present study confirmed that chromosome 2B contains more yellow rust resistance loci (24 MTAs). This finding is consistent with previous reports by Chen et al. (2012) and Mallard et al. (2005), who reported approximately 112 P*. striiformis* resistance genes on wheat chromosome 2B, for instance: *Yr9*, *Yr10*, *Yr15*, *Yr24/Yr26/YrCh42* (Cheng et al., 2014), *Yr29/Lr46* (Lan et al., 2014), *YrExp1* (McIntosh et al., 2020), and *YrH52* (Klymiuk et al., 2020). Similarly, the present study identified that chromosome 2B harbored many yellow rust resistance putative QTLs, and this finding was in line with the reports of Feng et al. (2018) and Khalil et al. (2022), who described several yellow rust resistance QTLs on the same chromosome, including Yr64, Yr65, YrTr1, YrAlp, YrH122, YrL693, YrC142, YrMY41, QYr.cau-1BS, QYrco.wpg-1BS.1, and QYrco.wpg-1BS.2. Interestingly, markers found in the current study are located at 74.20-793 Mbp.

A total of 54 QTLs were observed across all environments. Of the 54 QTLs, 17 putative QTLs were detected at Holeta, 3 at Kulumsa, 23 at Meraro, and 11 QTLs were observed in all environments (**Table 7**). Moreover, 22 of the 54 QTLs were observed across different growth stages, including 10 QTLs at two different growth stages, seven QTLs in three growth stages, one QTL in four growth stages, and two QTLs in all growth stages (**Table 8**). The failure to generally not repeat QTL effects across all environments could be due to site-specific QTL effects and different disease pressures. The prolonged and intense rains that characterize Meraro led to the largest natural yellow rust infestations across all growth stages. Climate factors, including lingering crop moisture and protracted periods of heavy rain, encourage disease infection and dissemination across the crop canopy (Helen and Sarah, 2015).

**Table 7:** Stable putative QTLs across all locations identified through bread wheat chromosomes for the coefficient of infection.

No	QTL	Chr	Position (bp)	Phenotypes _Location
1	qYrS.01	1A	16417326	YRF_Combined
2	qYrS.02	1A	337947849	YRM_Combined, YRM_Kulumsa, YRMM_Combined, YRMM_Kulumsa, YRPH_Meraro
3	qYrS.03	1A	512496605	YRF_Combined,
4	qYrS.04	1A	588261371	YRF_Combined, YRF_Meraro
5	qYrS.06	1B	73576935	YRM_Combined, YRM_Kulumsa, YRMM_Holeta, YRMM_Kulumsa & YRPH_Meraro
6	qYrS.09	1D	7734813	YRH_Holeta & YRPH_Holeta
7	qYrS.10	1D	49605264-54911805	YRH_Kulumsa, YRM_Kulumsa, YRMM_Kulumsa, YRF_Kulumsa, YRH_Kulumsa & YRPH_Combined
8	qYrS.12	2A	733216778-750201766	YRF_Holeta & YRPH_Combined
9	qYrS.14	2B	237588008-243083729	YRM_Holeta, YRH_Combined & YRH_Meraro
10	qYrS.17	2B	647897009	YRH_Combined, YRH_Holeta, YRMM_Holeta, YRPH_Combined & YRPH_Holeta
11	qYrS.22	2D	74936166	YRF_Combined-
12	qYrS.24	2D	591603469	YRF_Combined
13	qYrS.25	3A	250818410	YRF_Holeta, YRGM_Combined, & YRM_Holeta
14	qYrS.29	3B	20999249	YRF_Combined
15	qYrS.35	3D	569281482	YRF_Combined
16	qYrS.36	4A	521603853	YRM_Meraro
17	qYrS.37	4A	603746204	YRPH_Holeta
18	qYrS.38	4B	15149905	YRMM_Meraro & YRMM_Meraro
19	qYrS.42	5A	36021495	YRM_Meraro, YRF_Combined, YRF_Meraro, & YRF_Combined
20	qYrS.53	6B	675702168	YRH_Kulumsa, YRMM_Kulumsa, YRM_Meraro, YRH_Holeta & YRPH_Holeta
21	qYrS.55	7A	96563306-128909455	YRF_Holeta, YRH_Holeta, YRMM_Holeta & YRF_Meraro

QTL, Quantitative trait locus; Chr, Chromosomes; Phenotype_Location, coefficient of infection measured at individual or across locations (Holetta, Kulumsa, and Meraro); YRPH, coefficient of infection at pre-heading; YRH, coefficient of infection at heading; YRF, coefficient of infection at flowering; YRMM, coefficient of infection at mid-maturity and YRM, coefficient of infection at maturity.

**Table 8:** Stable putative QTLs across locations identified through bread wheat chromosomes for yellow rust resistance.

No	QTLs	Chr	Position	Growth stage	Phenotypes _Location
1	qYrS.02	1A	337947849	3	YRM_Combined, YRM_Kulumsa, YRMM_Combined, YRMM_Kulumsa, YRPH_Meraro
2	qYrS.06	1B	73576935	3	YRM_Combined, YRM_Kulumsa, YRMM_Holeta, YRMM_Kulumsa & YRPH_Meraro
3	qYrS.08	1B	605007601-687536131	2	YRPH_Kulumsa & YRH_Kulumsa
4	qYrS.09	1D	7734813	2	YRH_Holeta & YRPH_Holeta
5 *	qYrS.10	1D	49605264-54911805	5	YRH_Kulumsa, YRM_Kulumsa, YRMM_Kulumsa, YRF_Kulumsa, YRH_Kulumsa & YRPH_Combined
6	qYrS.12	2A	733216778-750201766	2	YRF_Holeta & YRPH_Combined
7	qYrS.13	2B	74203726	2	YRM_Holeta & YRMM_Holeta
8	qYrS.14	2B	237588008-243083729	2	YRM_Holeta, YRH_Combined & YRH_Meraro
9	qYrS.17	2B	647897009	3	YRH_Combined, YRH_Holeta, YRMM_Holeta, YRPH_Combined & YRPH_Holeta
10	qYrS.20	2B	759523837-768053989	3	YRM_Holeta, YRMM_Holeta, YRM_Kulumsa, YRM_Holeta, YRMM_Holeta & YRF_Meraro
11	qYrS.25	3A	250818410	2	YRF_Holeta & YRM_Holeta
12	qYrS.27	3A	639713075	3	YRF_Holeta, YRM_Holeta & YRMM_Holeta
13	qYrS.28	3A	733636208-739951852	3	YRH_Meraro, YRM_Meraro, YRMM_Meraro & YRM_Meraro
14	qYrS.34	3D	339476105	2	YRH_Kulumsa & YRPH_Holeta
15	qYrS.42	5A	36021495	2	YRM_Meraro, YRF_Combined, YRF_Meraro, & YRF_Combined
16	qYrS.43	5A	429474426	2	YRF_Holeta, & YRMM_Holeta
17 *	qYrS.53	6B	675702168	5	YRH_Kulumsa, YRMM_Kulumsa, YRM_Meraro, YRF_Holeta & YRPH_Holeta
18	qYrS.54	7A	12800907-50346076	5	YRF_Kulumsa, YRH_Kulumsa, YRM_Kulumsa, YRMM_Kulumsa & YRF_Meraro
19	qYrS.55	7A	96563306-128909455	4	YRF_Holeta, YRH_Holeta, YRMM_Holeta & YRF_Meraro
20	qYrS.56	7D	9295305-14621383	3	YRH_Holeta, YRH_Holeta, YRH_Holeta, YRF_Holeta, & YRMM_Holeta

QTL, Quantitative trait locus; Chr, Chromosomes; Phenotype_Location, coefficient of infection measured at individual or across locations (Holetta, Kulumsa, and Meraro); YRPH, coefficient of infection at pre-heading; YRH, coefficient of infection at heading; YRF, coefficient of infection at flowering; YRMM, coefficient of infection at mid-maturity and YRM, coefficient of infection at maturity; *=QTLs stable across all environments and all growth stages.

Even though it is challenging to compare the positions of QTLs from different studies because of the different mapping methodologies, marker systems, and mapping populations employed, some QTLs found in this study coincided with the mapping positions of previously reported yellow rust resistant genes in the literature. Similar to the current study, several earlier studies reported yellow rust-resistant QTLs on 1A (Fu et al., 2019; Tehseen et al., 2020; Yao et al., 2021; Baranwal et al., 2022; Bouvet et al., 2022), on 1B (Losert et al., 2017; Alemu et al., 2020; Baranwal et al., 2022), on 1D (Long et al., 2019; Jia et al., 2020; Zhang et al., 2021; Baranwal et al., 2022; Kumar et al., 2023) on 2A (Tehseen et al., 2020; Zhang et al., 2021; Zhang et al., 2021; Baranwal et al., 2022), on 2B (Li et al., 2020), on 2D (Mu et al., 2020; Baranwal et al., 2022), on 3A (Tehseen et al., 2022), on 3B (Zegeye et al., 2014; Kumar et al., 2020), on 3D (Bouvet et al., 2022), on 4A (Mu et al., 2020), on 4B (Alemu et al., 2020), on 4D (Rollar et al., 2021), on 5A (Zhang et al., 2021), on 5B (Lu et al., 2014), on 5D (Zhang et al., 2021), on 6A (Baranwal et al., 2022), on 6B (Zhang et al., 2021) on 7A (Yel et al., 2019; Alemu et al., 2020; Tehseen et al., 2020; Baranwal et al., 2022), and on 7D (Long et al,. 2019; Rollar et al., 2021).

Out of the 54 putative QTLs detected in the current study, 12 QTLs (qYrS.11 on chromosome 1D; qYrS.16, qYrS.17, and qYrS.18 on chromosome 1B; qYrS.26 on chromosome 3A; qYrS.31 and qYrS.32 on chromosome 3B; qYrS.40 on chromosome 4B; qYrS.45 and qYrS.46 on chromosome 5D; qYrS.50 on chromosome 6B; and qYrS.58 on chromosome 7D) were not reported in previous literature on wheat and hence could potentially be novel (**Supplementary Table 8**).

On chromosome 1A, the discovery of defense-related candidate genes like TraesCS1A02G325400 and TraesCS1D02G018500on in the significant markers suggests that the observed QTL areas are functionally associated with plant defense mechanisms against fungal infections. Furthermore, genes such as *TraesCS1A02G183400*, found on the qYrS.02 QTL region on chromosome 1A, and *TraesCS1B02G480300*, found on the qYrS.08 QTL region on chromosome 1B, are involved in plants’ systemic acquired resistance to pathogen infections. According to Lawton et al. (1995), systemic acquired resistance is a broad-spectrum resistance acquired following the initial localized infection of plants by pathogens. Genes such as *TraesCS1B02G324300*, found in qYrS.07, and *TraesCS6B02G017900*, in the qYrS.49 region, on chromosome 1B and 6B, respectively, control the MAPK cascades that signal various defense reactions, such as the production of or signaling of plant stress, gene activation, and hypersensitive response cell death. Additionally, it is assumed that the majority of the genes similar to the discovered key markers are involved in the manufacture of salicylic acid, an important plant hormone well known for mediating host responses to pathogen infection (Lefevere et al., 2020; Mekonnen et al., 2021).

Due to the lack of shared genetic effects for yellow rust resistance, the majority of the QTLs discovered for vegetative, yield-related, and phenological traits did not overlap with those discovered for yellow rust resistance traits. Numerous associations between agronomic traits and yellow rust resistance traits were non-significant, had minimal to weak negative coefficients, or were both, showing that the traits were independent. However, this study showed that some putative QTLs determined for agronomic traits and putative QTLs identified by yellow rust resistance data overlapped. For example, the potential QTL identified on 1B for grain yield was co-mapped with qYrS.07, identified for yellow rust resistance at the maturity stage. This implies that these traits are controlled by similar loci. As anticipated, grain yield may be impacted by the disease’s vertical advancement rate. The putative QTL determined for plant height on chromosome 2A is co-mapped with qYrS.12, identified for the resistance of yellow rust at flowering. That is consistent with the finding of Mekonnen et al. (2021), who stated that the putative QTL controlling grain yield is co-mapped with the Septoria tritici blotch progress coefficient on chromosome 1A. Traits that facilitate escape, such as tallness, reduce disease resistance by limiting or delaying the vertical spread of the spores up the plant, probably leading them to be scored as resistant when they are inherently not resistant (Miedaner et al., 2013).

## 5 Conclusion

Stripe rust caused by the fungus *P. striiformis* is the major bottleneck to wheat production worldwide, including in Ethiopia. The use of resistant varieties is a durable, effective, and eco-friendly way to control crop diseases. Genome-wide association analysis is a powerful tool to dissect the genetic basis of disease resistance in crop plants. In this study, genomic regions underlying adult-plant resistance to yellow rust were explored using genome-wide scanned SNPs and multi-environment-derived phenotype data in a bread wheat association panel. The analysis revealed that yellow rust resistance is highly heritable e (h^2 ^= 60%), and hence could be improved by exploiting the resistance source through selection breeding. The study also found 148 SNPs that were significantly associated with strong yellow rust resistance, pointing to 54 QTLs. Some of these putative QTLs were consistently identified at all growth stages, and hence, they could be considered major genomic loci containing combinations of genes conferring resistance to *P. striiformis* across all growth stages of wheat. Functional dissection of the detected QTL regions in the wheat database identified several defense-related candidate genes involved in plant resistance against fungal infections, systemic acquired resistance, and MAPK pathways that are relevant in signaling numerous plant defense systems. The identified candidate genes close to the detected SNPs can be targeted to disclose the actual genes underlying the target trait in the association loci. Most of the detected putative QTLs shared similar chromosomal positions with previously reported genes and QTLs. However, their use in marker-assisted resistance breeding needs validation. Overall, the study confirmed that the association panel possessed considerable *P. striiformis* resistance alleles that could be deployed into the high-yielding but yellow rust-susceptible wheat varieties. The detected QTLs can be used in a wheat resistance breeding program to develop broad-spectrum and durable-resistant wheat varieties against the rapidly evolving wheat pathogen *P. striiformis.*

## Data availability statement

The datasets presented in this study can be found in online repositories. The names of the repository/repositories and accession number(s) can be found below: 10.5061/dryad.7sqv9s4s4.

## Author contributions

TM: Conceptualization, Formal analysis, Funding acquisition, Supervision, Validation, Writing – review & editing. GA: Conceptualization, Data curation, Formal analysis, Investigation, Methodology, Software, Writing – original draft, Visualization. TH: Conceptualization, Resources, Supervision, Validation, Writing – review & editing. MK: Conceptualization, Supervision, Validation, Writing – review & editing. KT: Conceptualization, Funding acquisition, Project administration, Resources, Supervision, Writing – review & editing. SG: Conceptualization, Resources, Writing – review & editing.
