# Supplementary material for: Genetic architecture of adult-plant resistance to stripe rust in bread wheat (Triticum aestivum L.) association panel
Source: Front Plant Sci. 2023 Dec 7;14:1256770. doi: 10.3389/fpls.2023.1256770 (PMC10733515; doi:10.3389/fpls.2023.1256770)
Supplement: Supplementary file 1 [file DataSheet_1.docx]

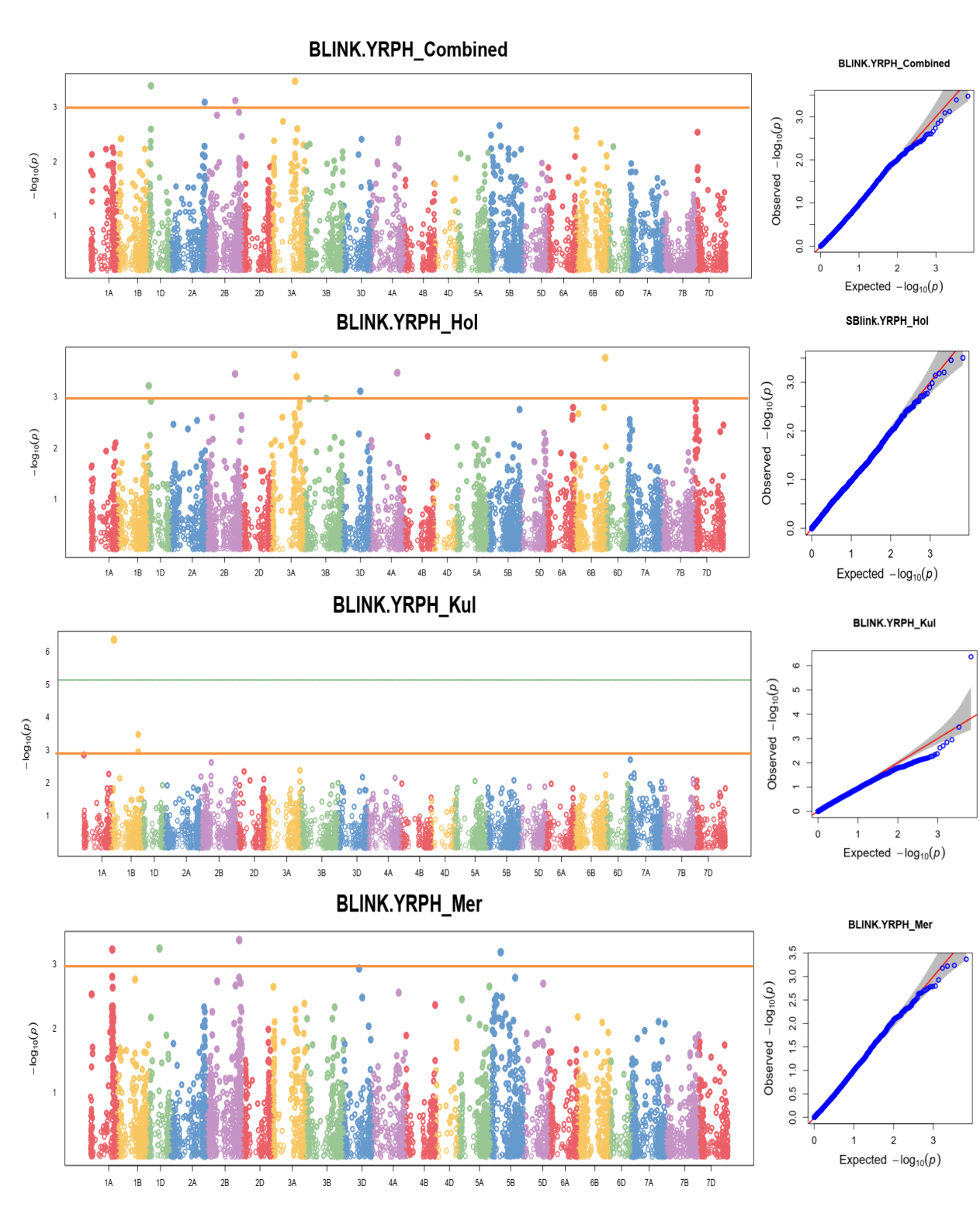


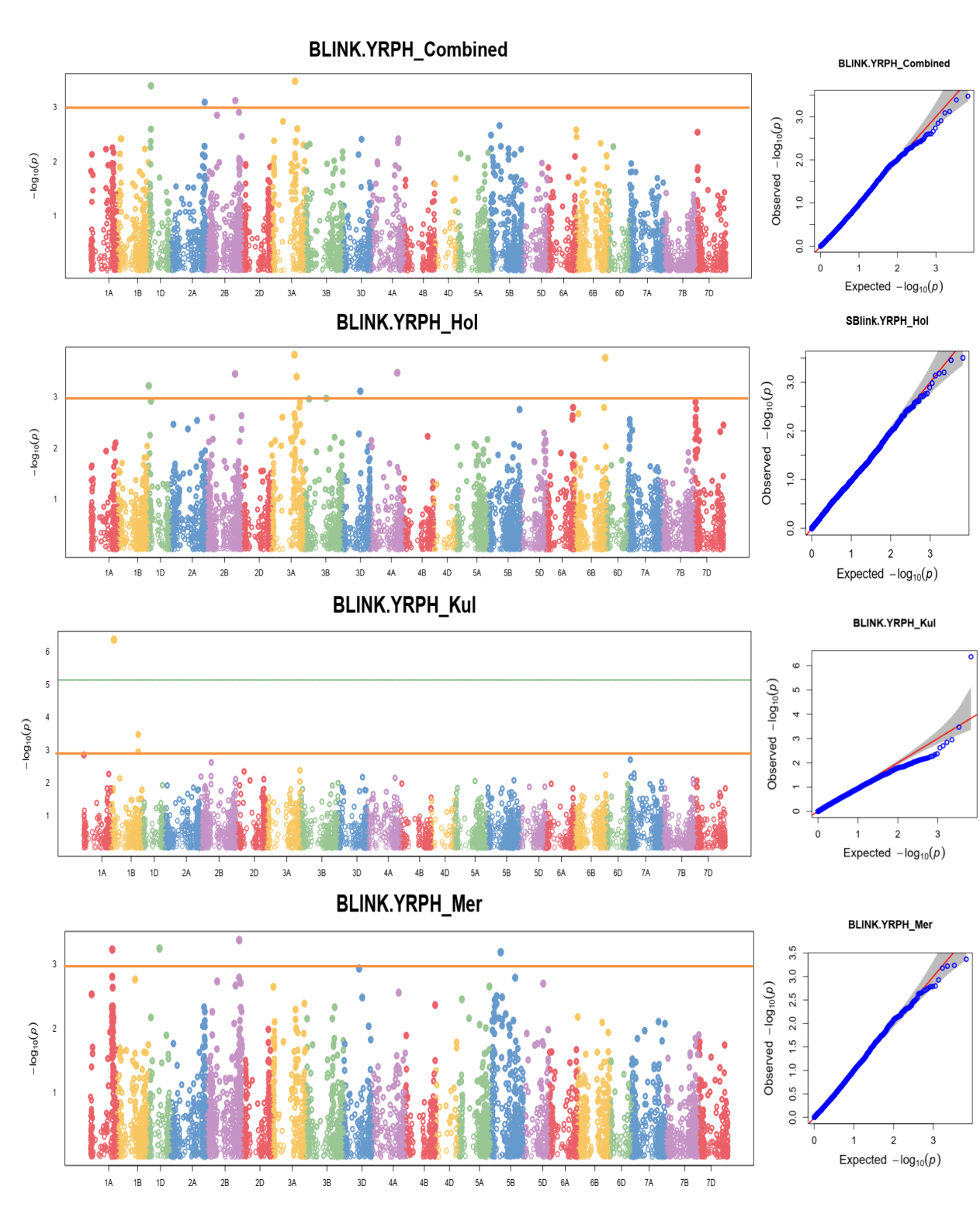


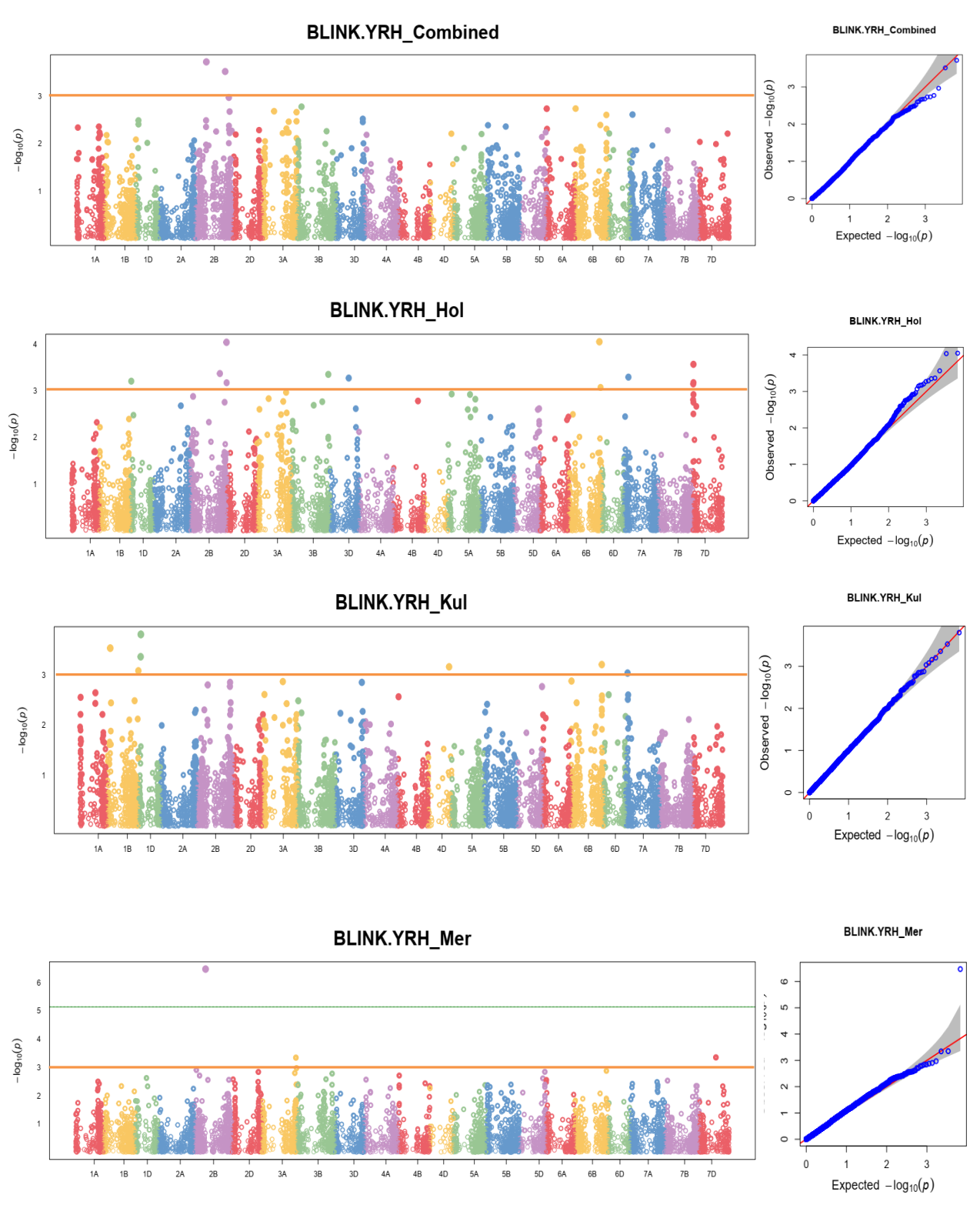


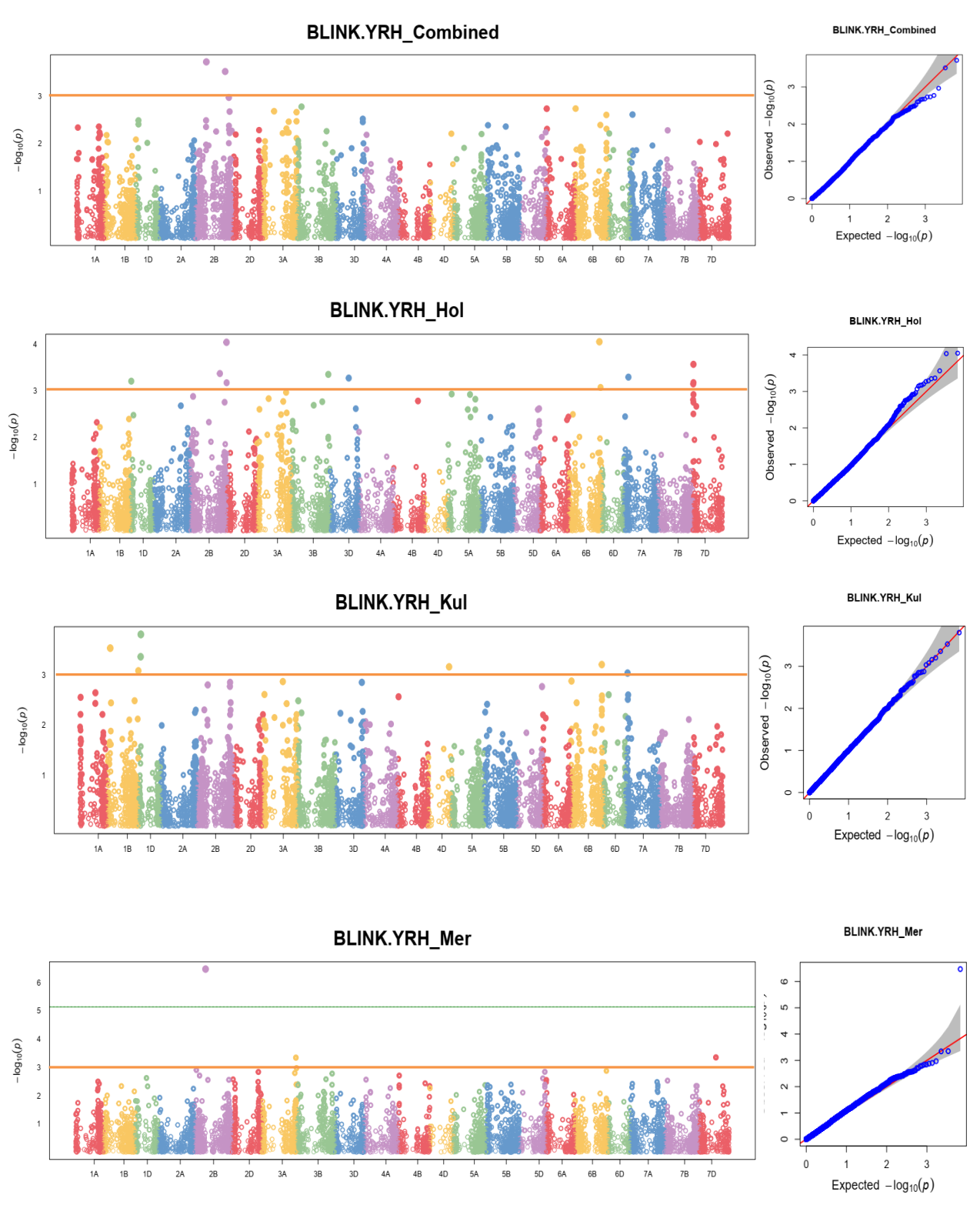


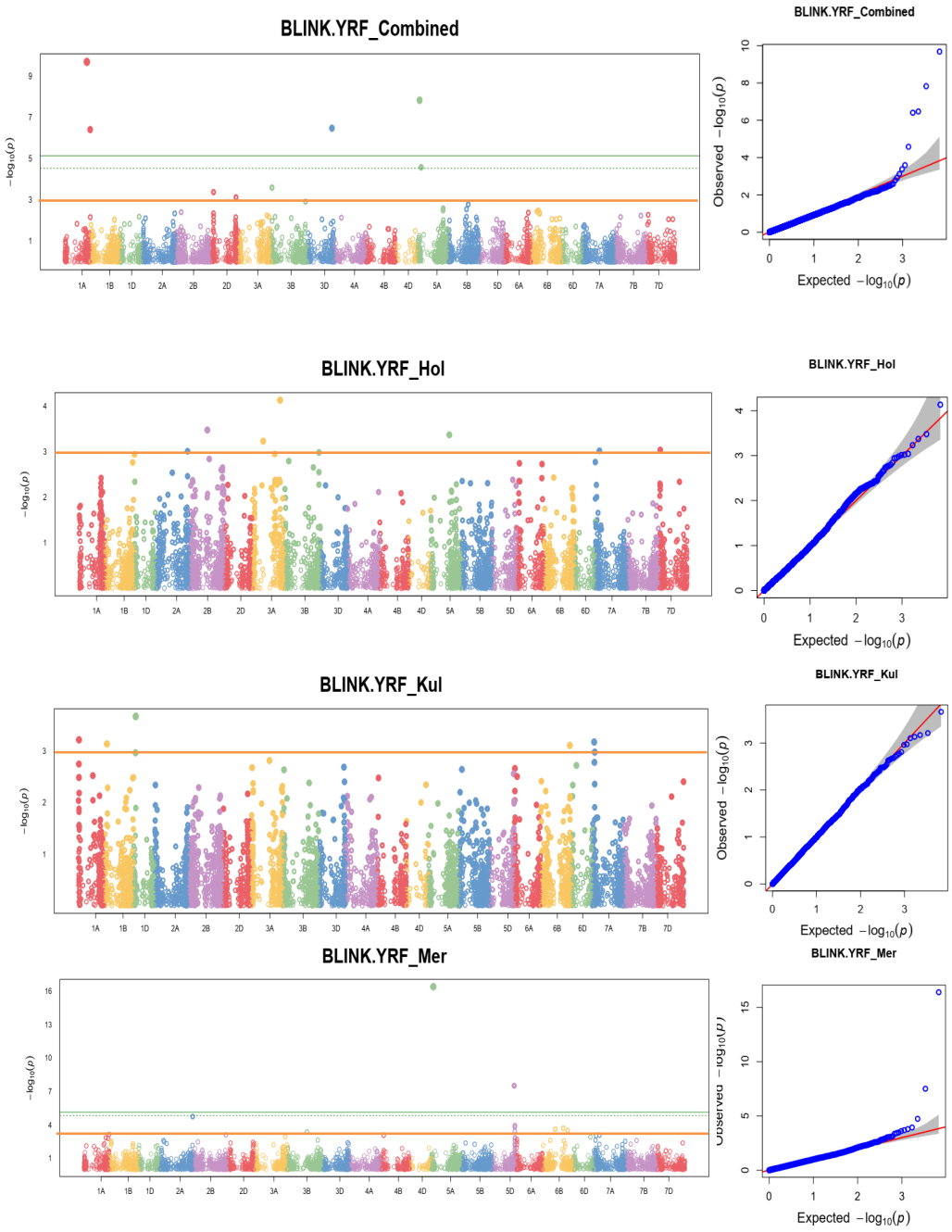

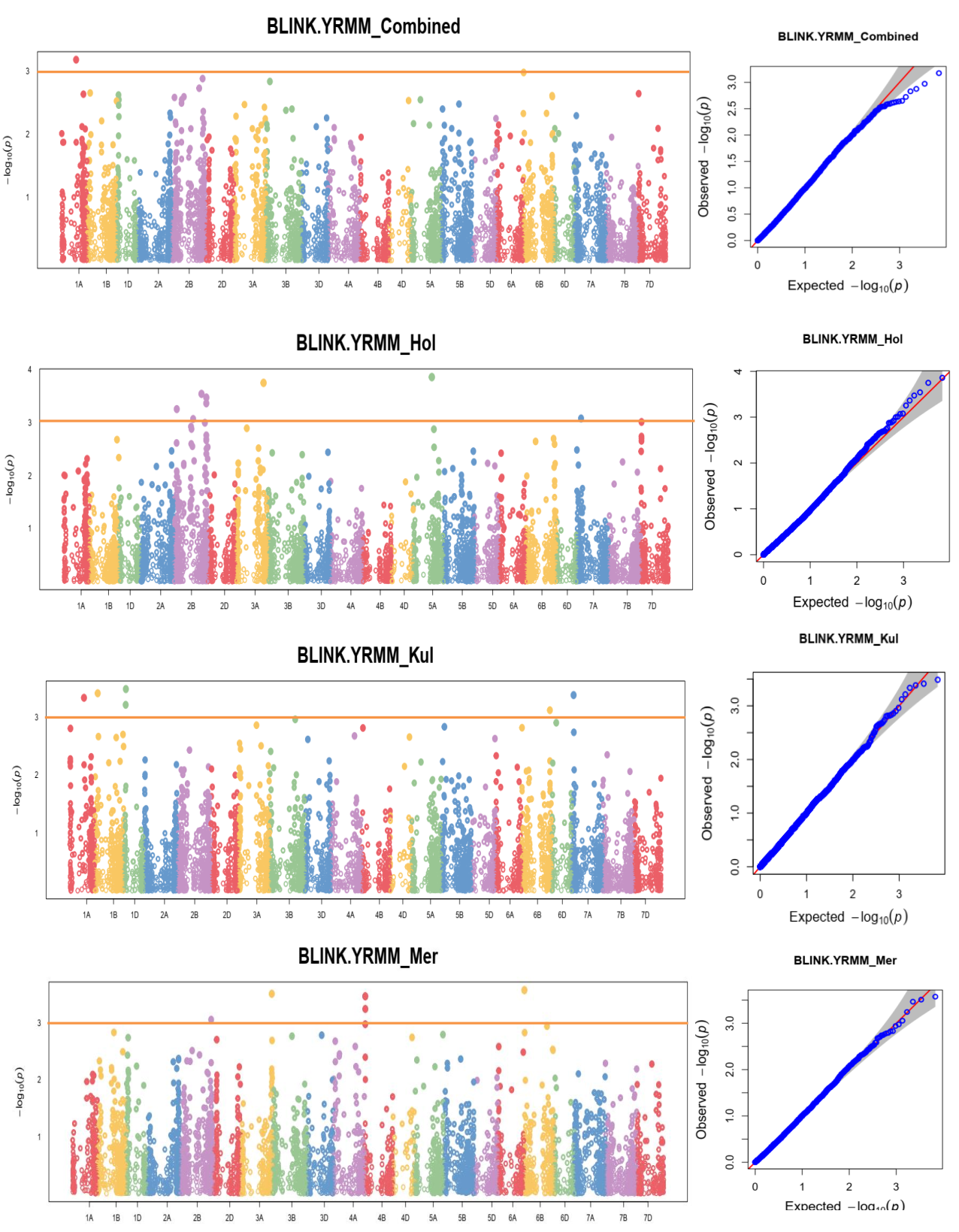


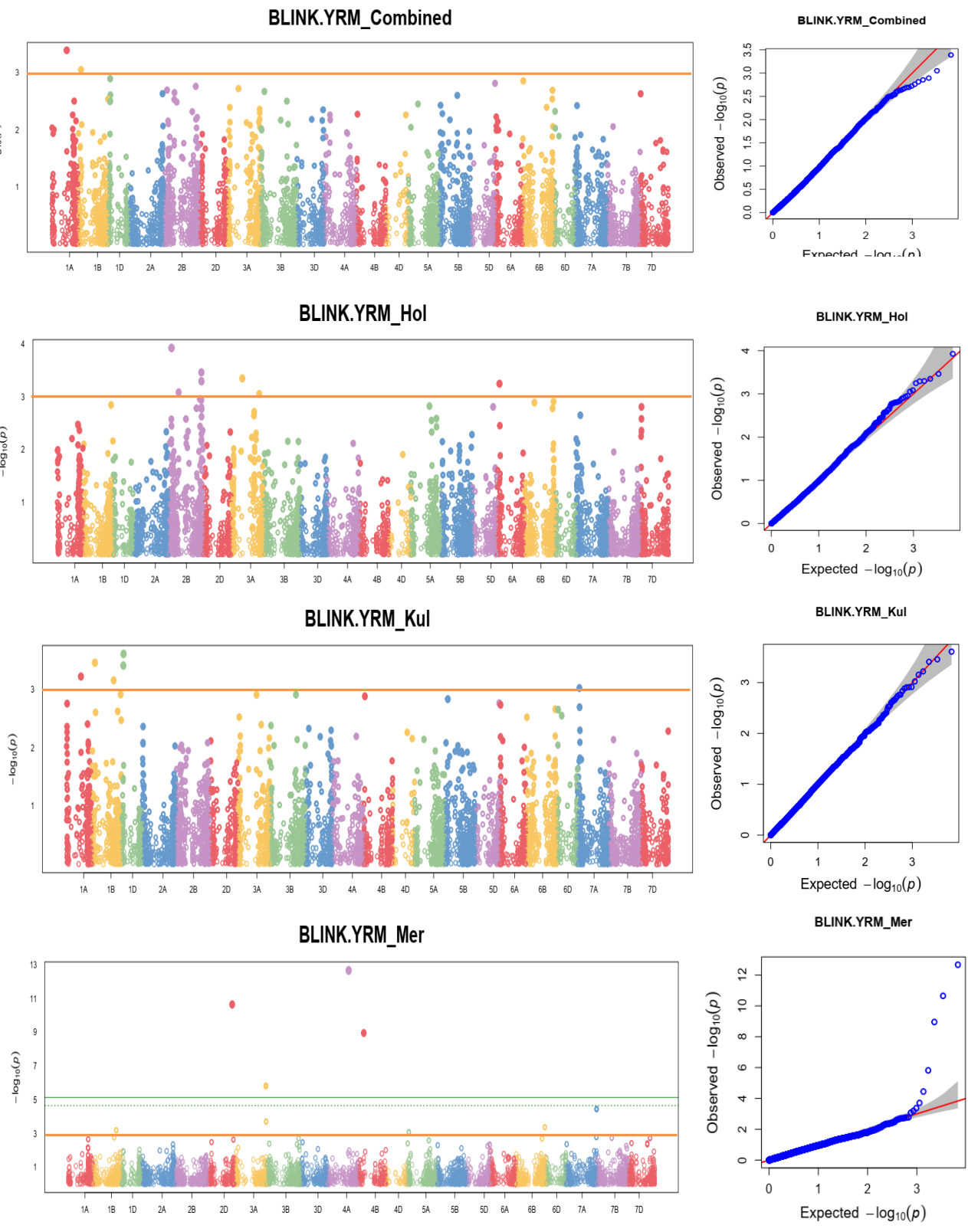


**Supplementary Figure 1**. Manhattan plots for yellow rust disease severity traits and GWAS scans resulting in significant associations. Each dot represents an SNP. On the x-axis is the genomic position of the SNPs on the corresponding chromosomes indicated in different colors. On the y-axis is the *-log10* of the *p-value* depicting the significance of the association test. The horizontal orange line is the nominal *p-*value 0.001 significance threshold used in the association analysis of YRPH = coefficient of infection at pre-heading, YRH = coefficient of infection at heading, YRF = coefficient of infection at flowering, YRMM = coefficient of infection at mid-maturity, and YRM = coefficient of infection at maturity. The quantile-quantile (Q-Q) plots at the right side of the Manhattan plots indicate how well the used BLINK model accounted for population structure and kinship for each of the disease traits. In each plot, the observed *–log (p-values)* from the fitted GWAS models (y-axis) are compared with their expected value (x-axis) under the null hypothesis of no association with the trait. Each blue dot represents a single nucleotide polymorphism; the red line is the model for no association.
