# Supplementary material for: Genetic architecture of adult-plant resistance to stripe rust in bread wheat (Triticum aestivum L.) association panel
Source: Front Plant Sci. 2023 Dec 7;14:1256770. doi: 10.3389/fpls.2023.1256770 (PMC10733515; doi:10.3389/fpls.2023.1256770)
Supplement: Supplementary file 2 [file DataSheet_2.doc]

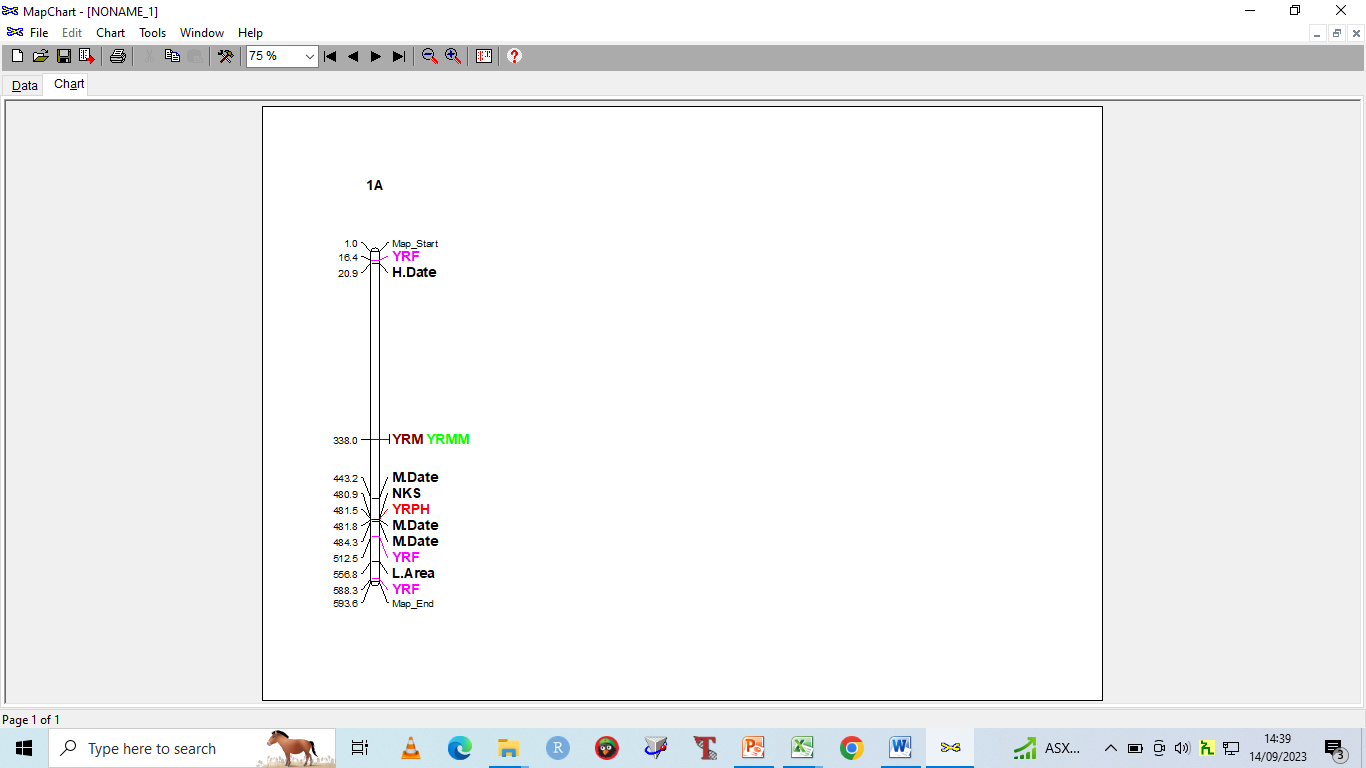

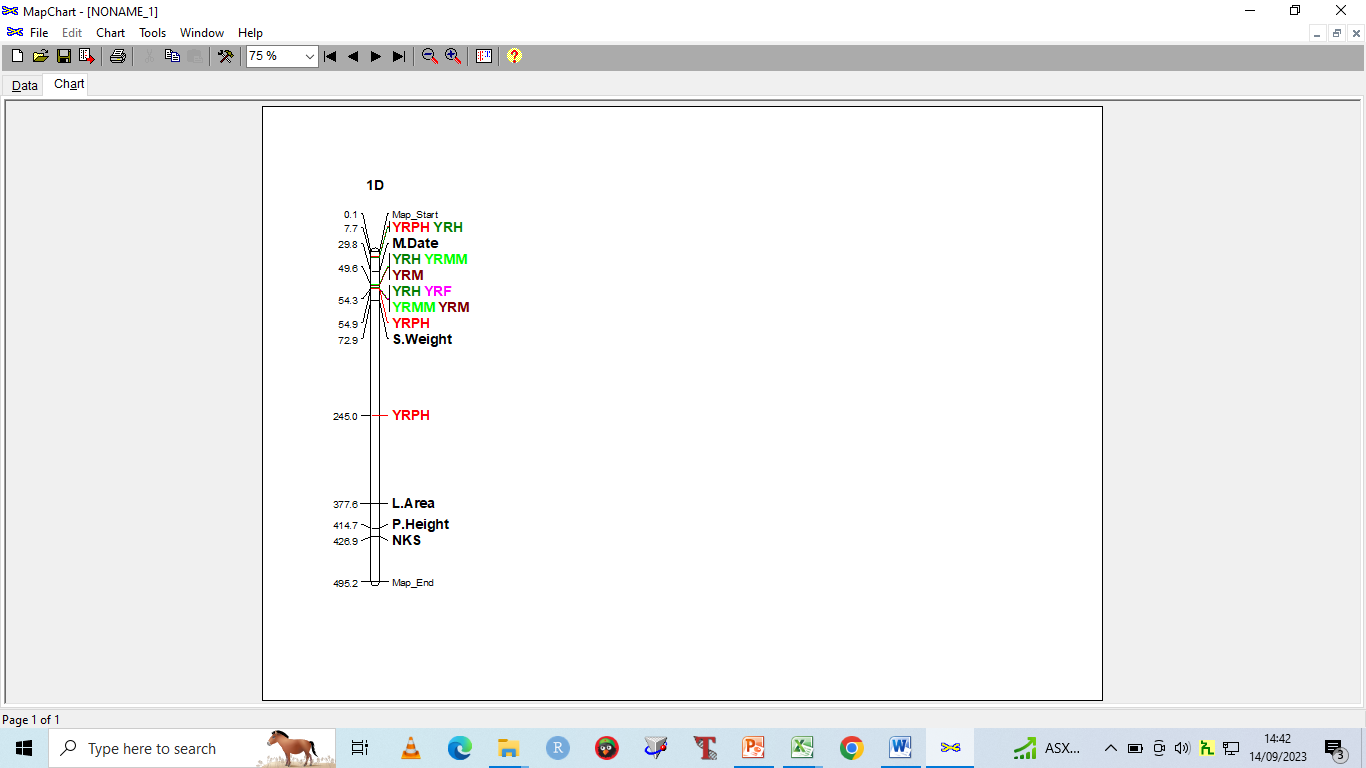


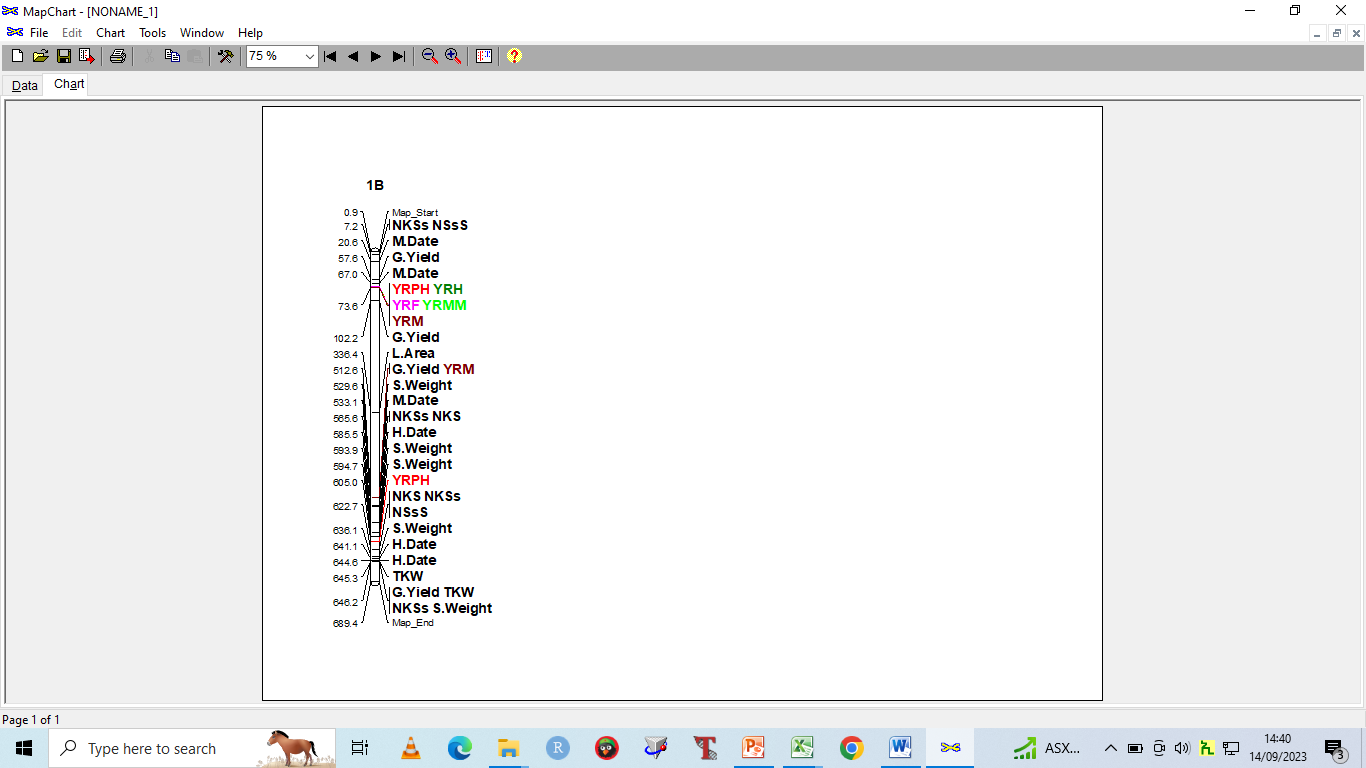


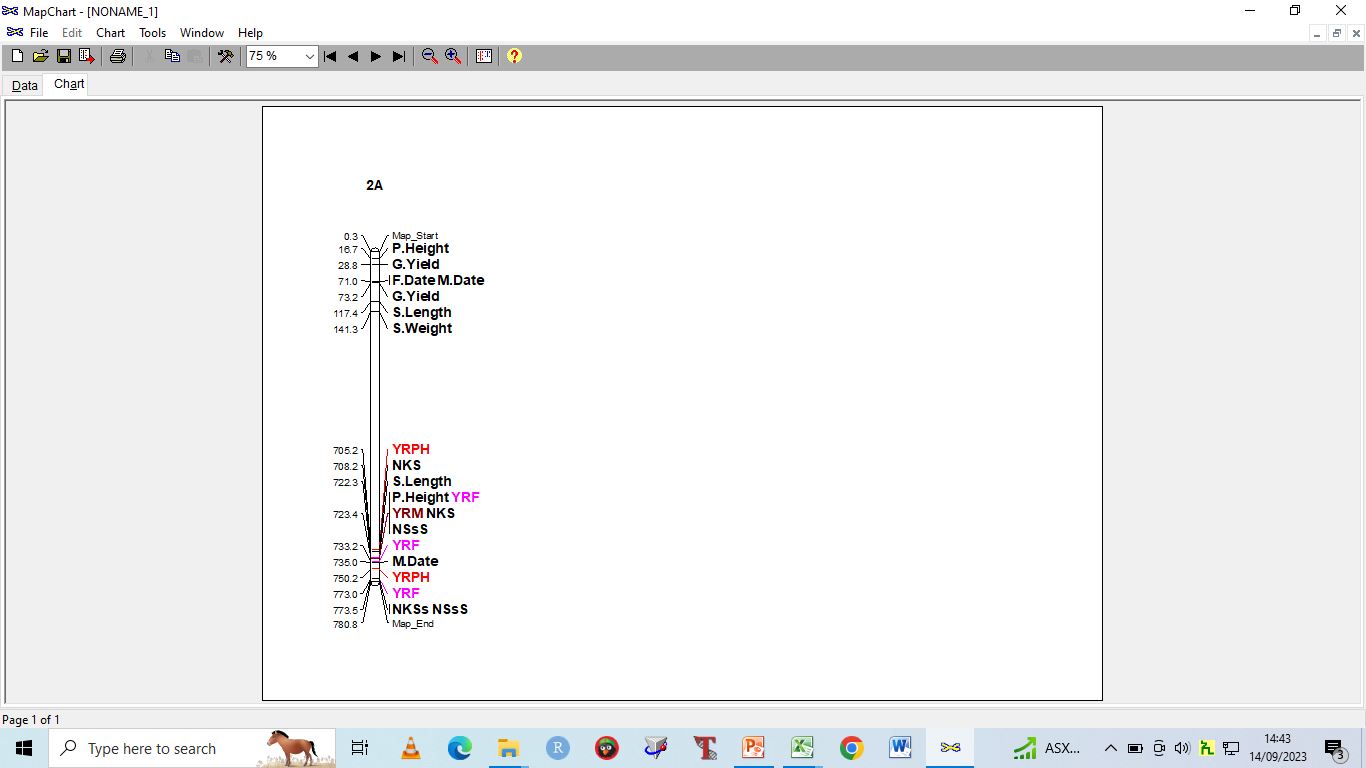

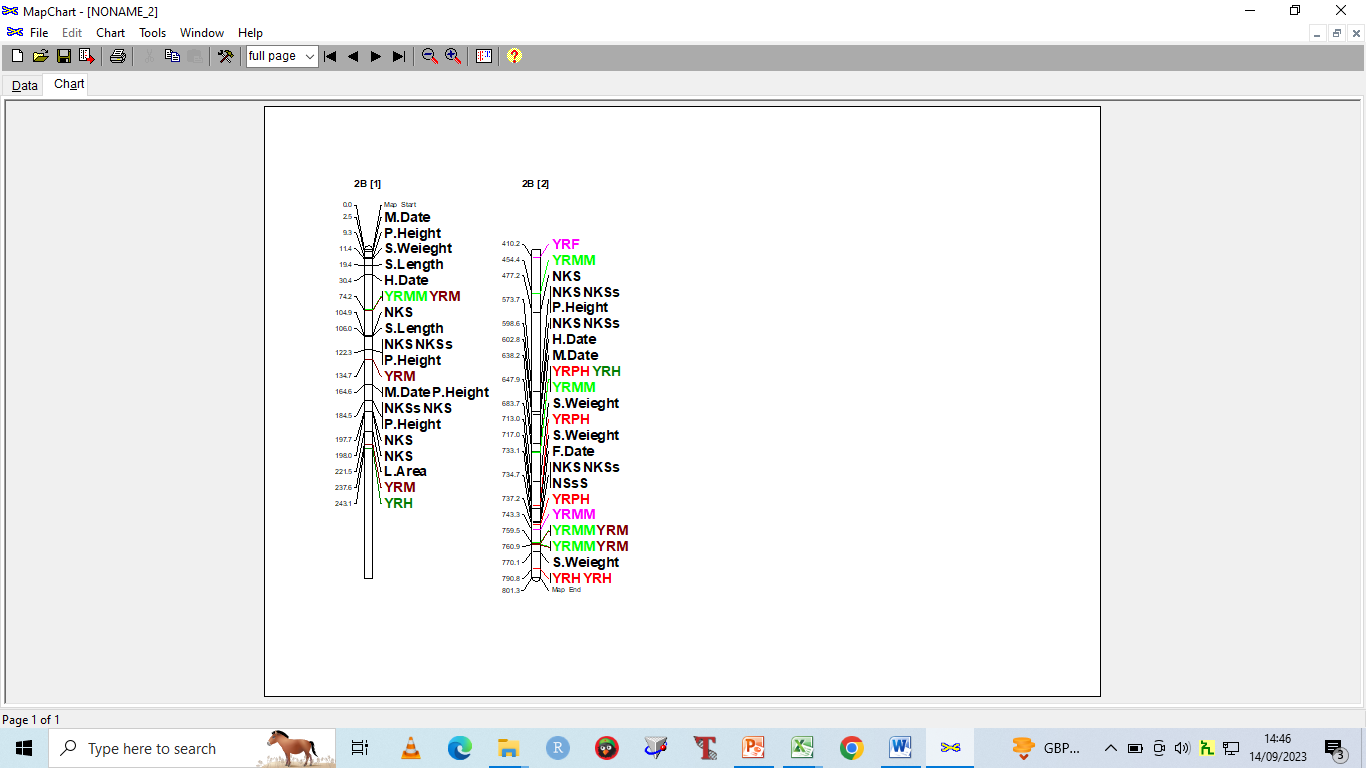


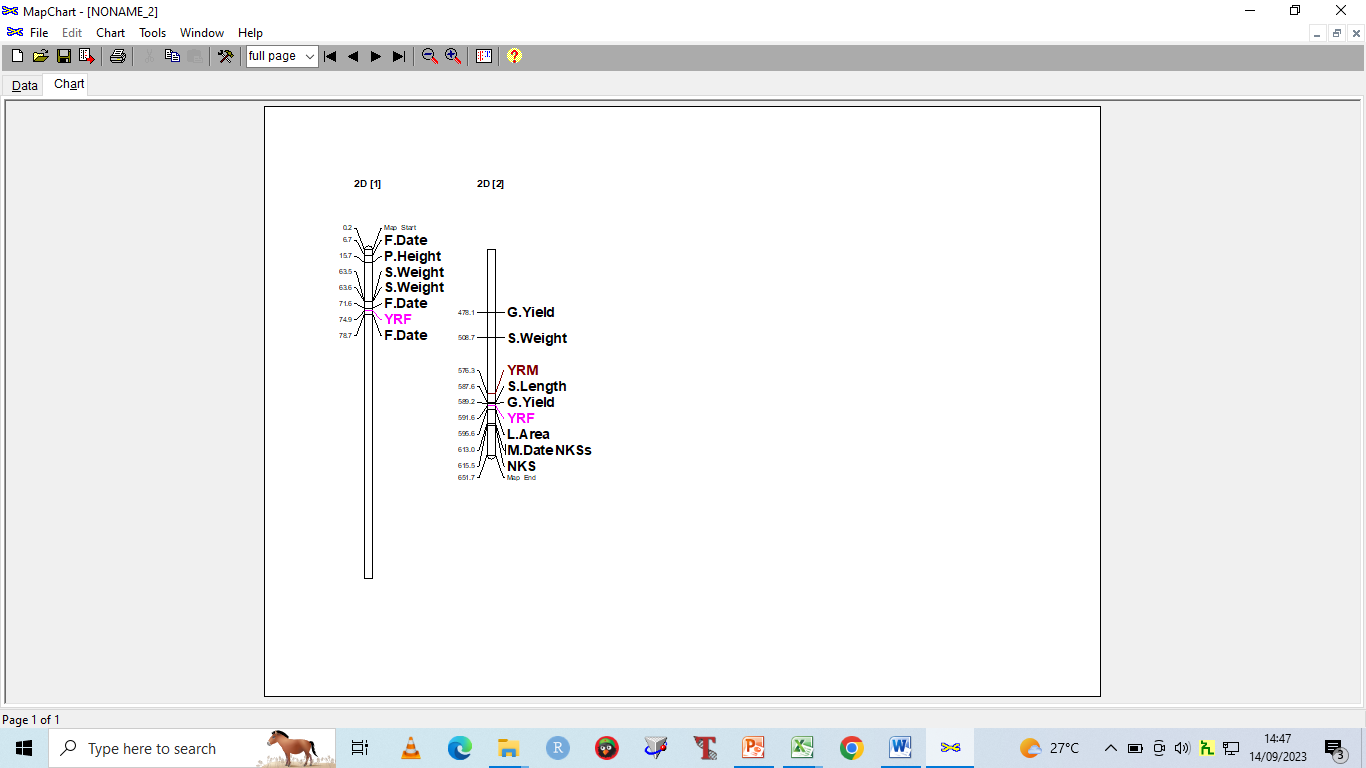

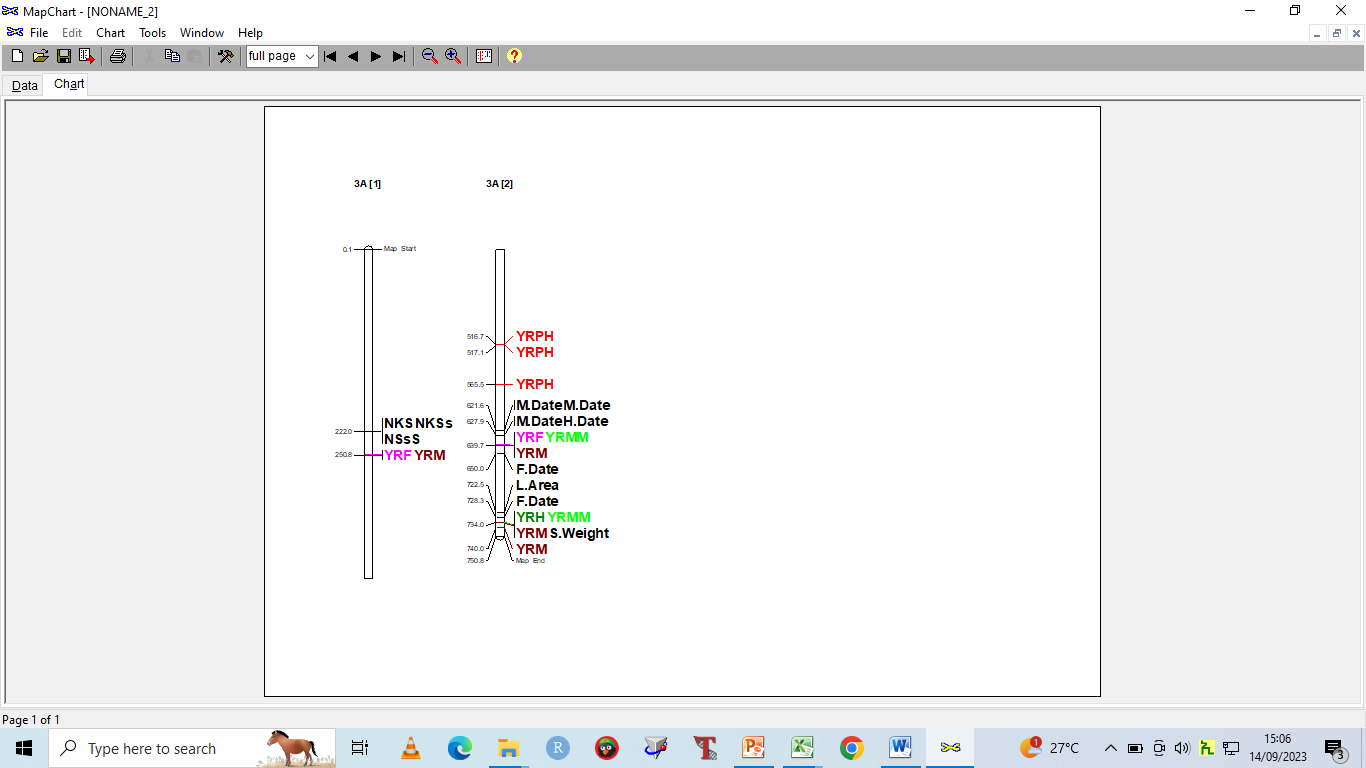


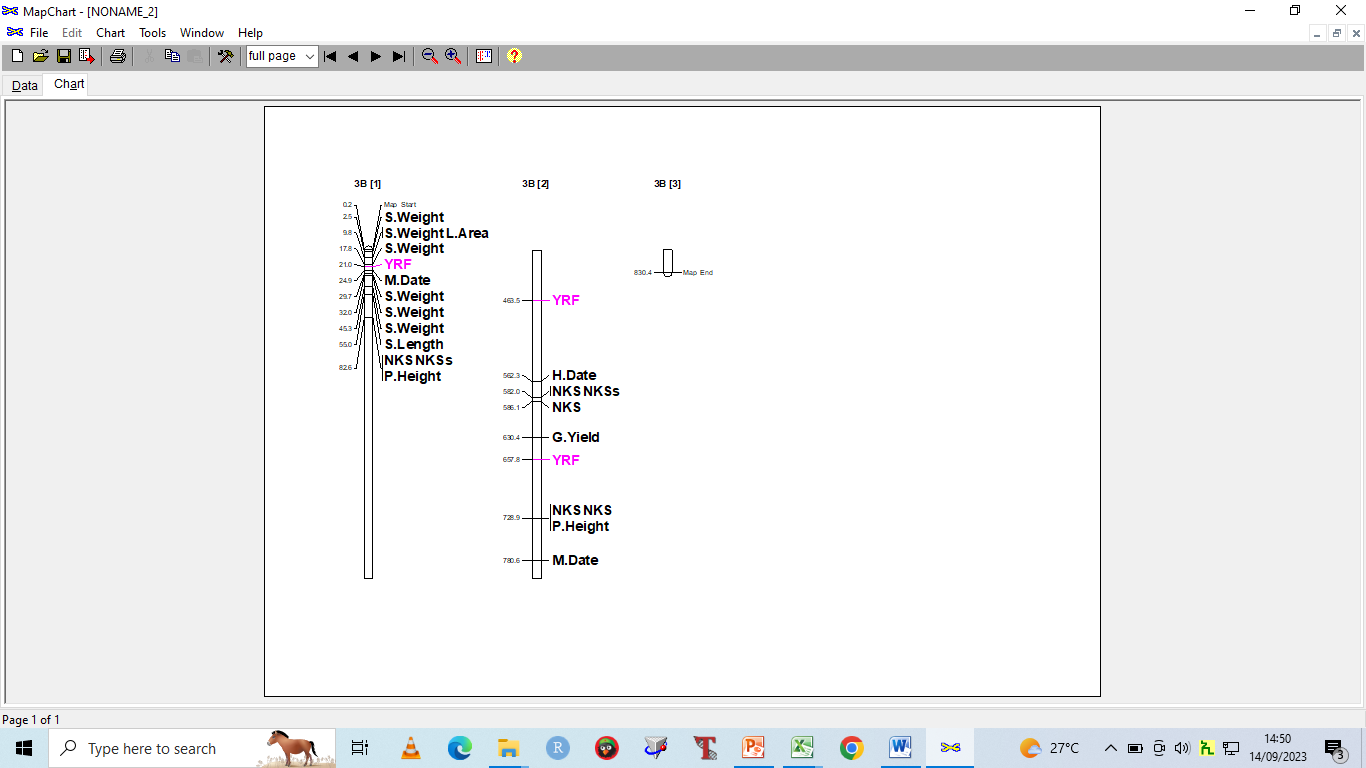

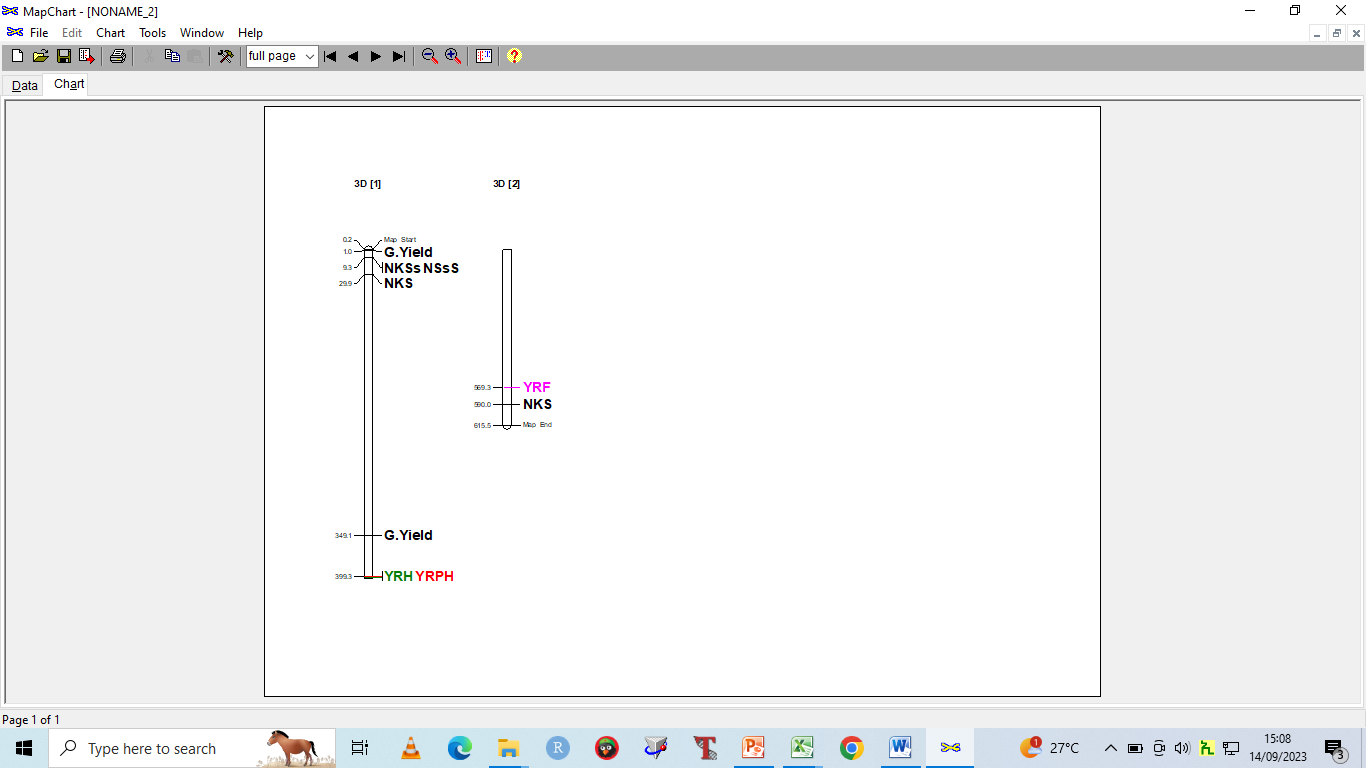


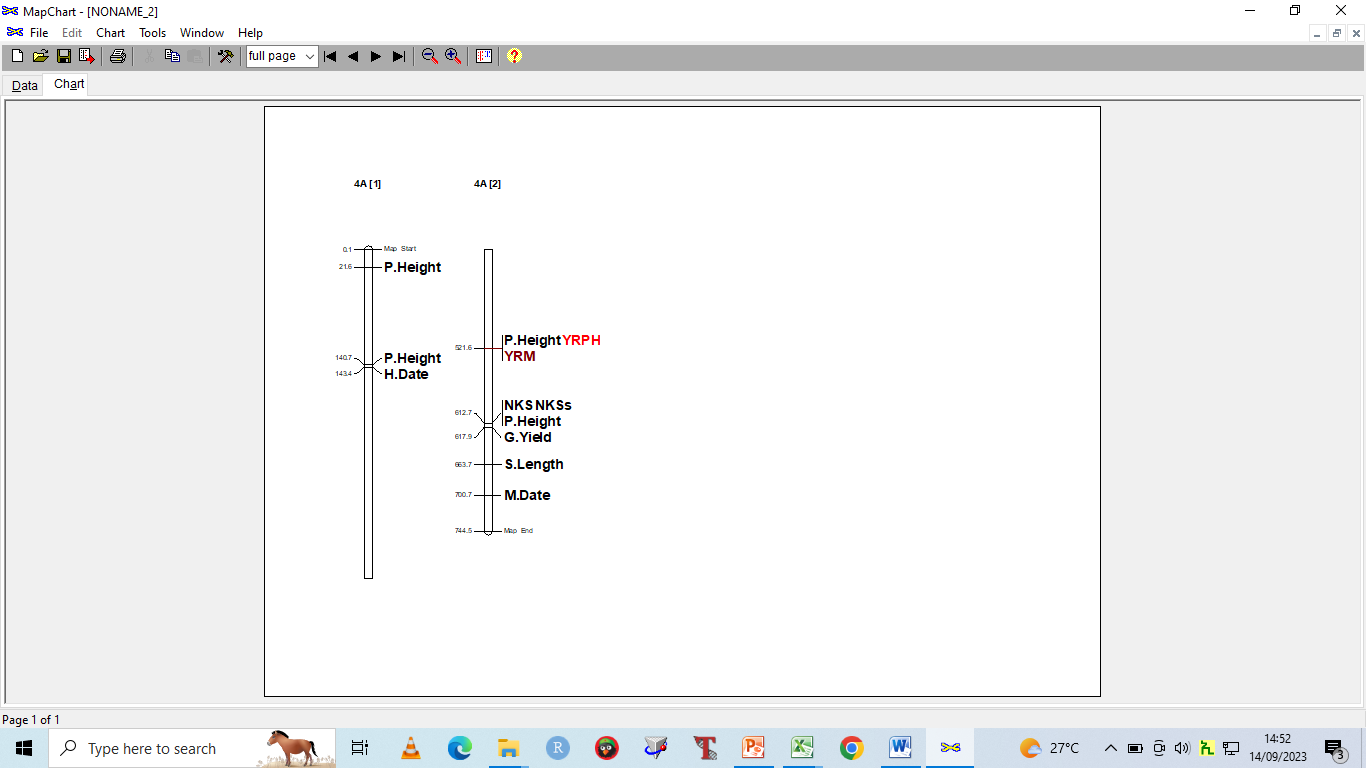

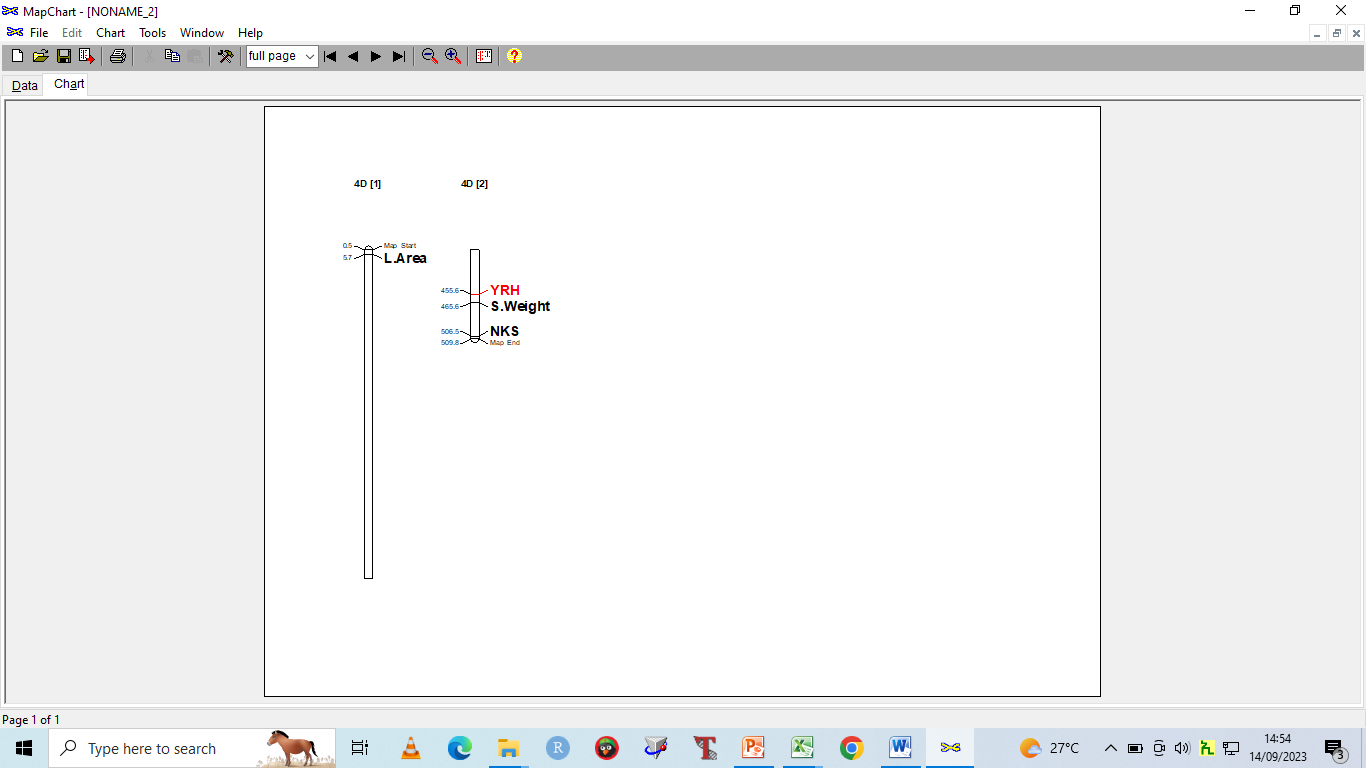

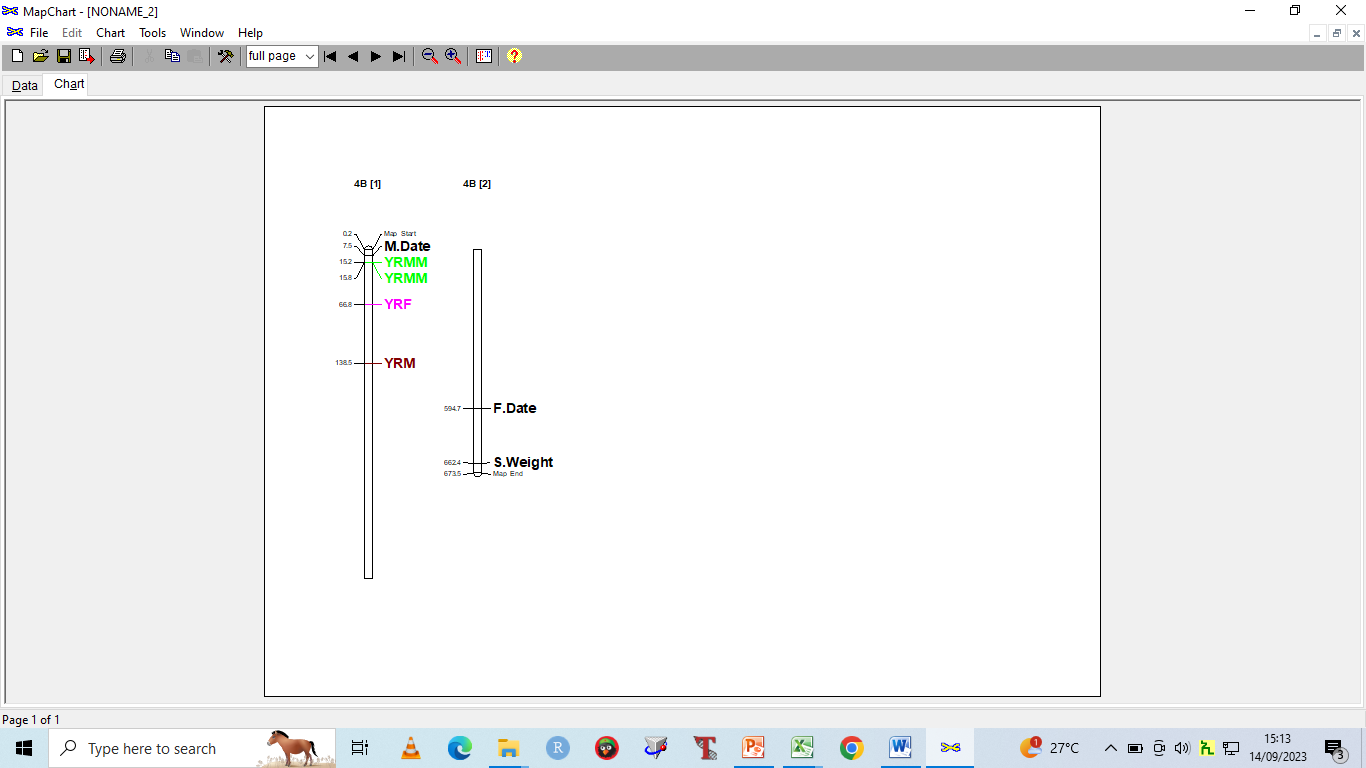


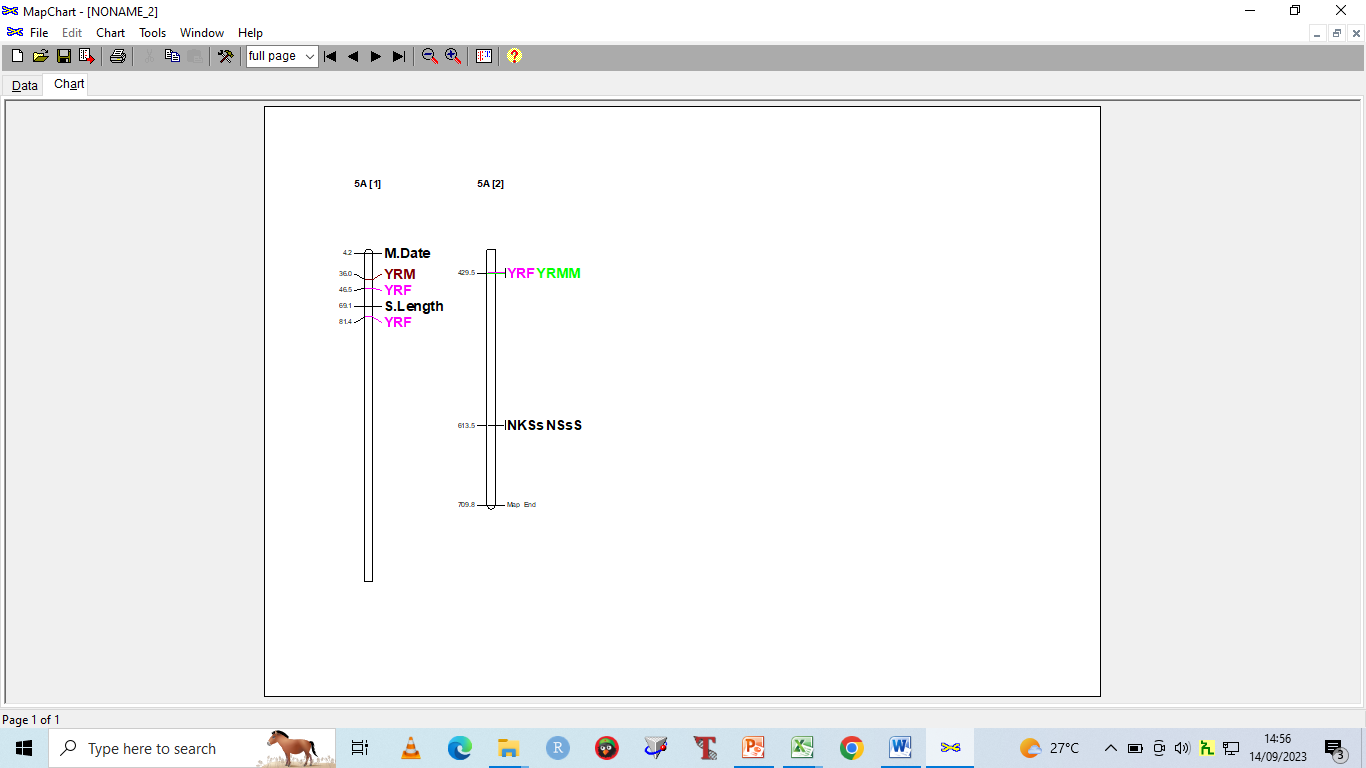

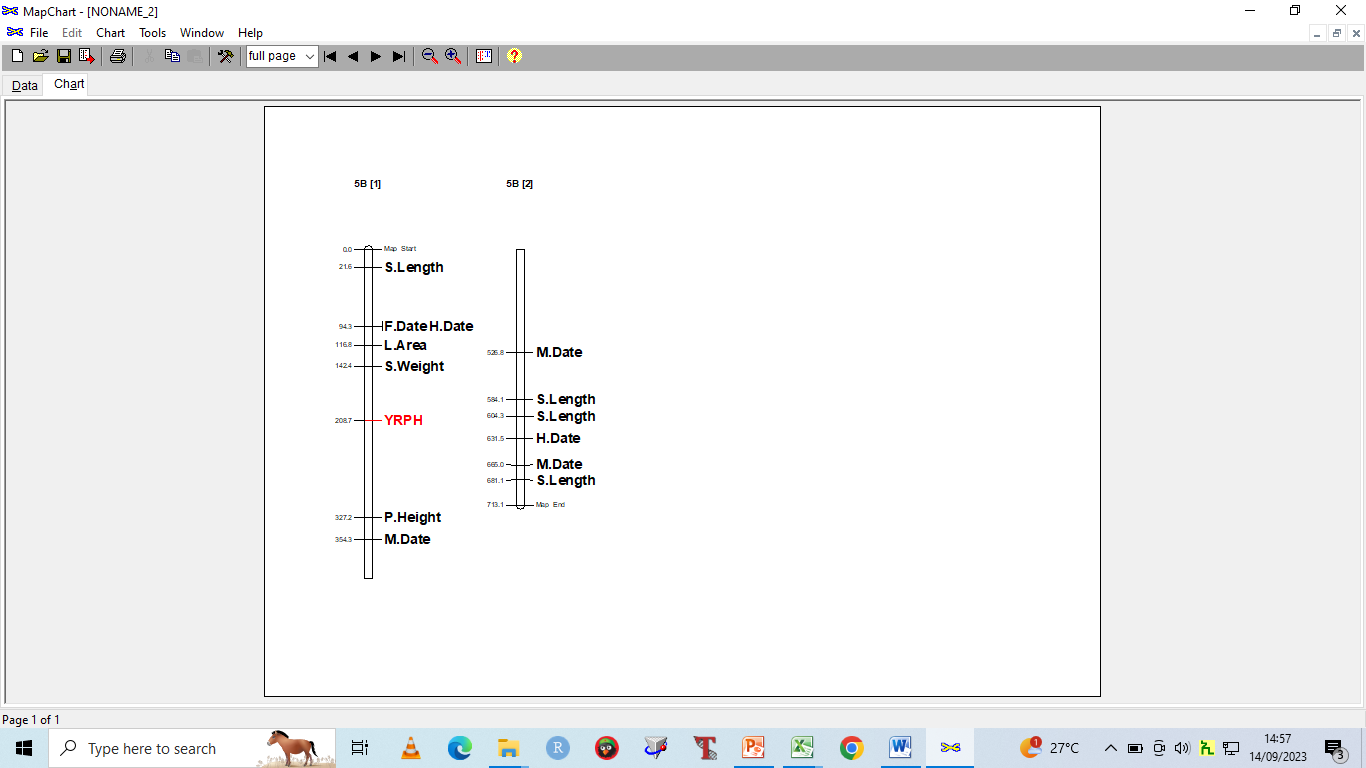


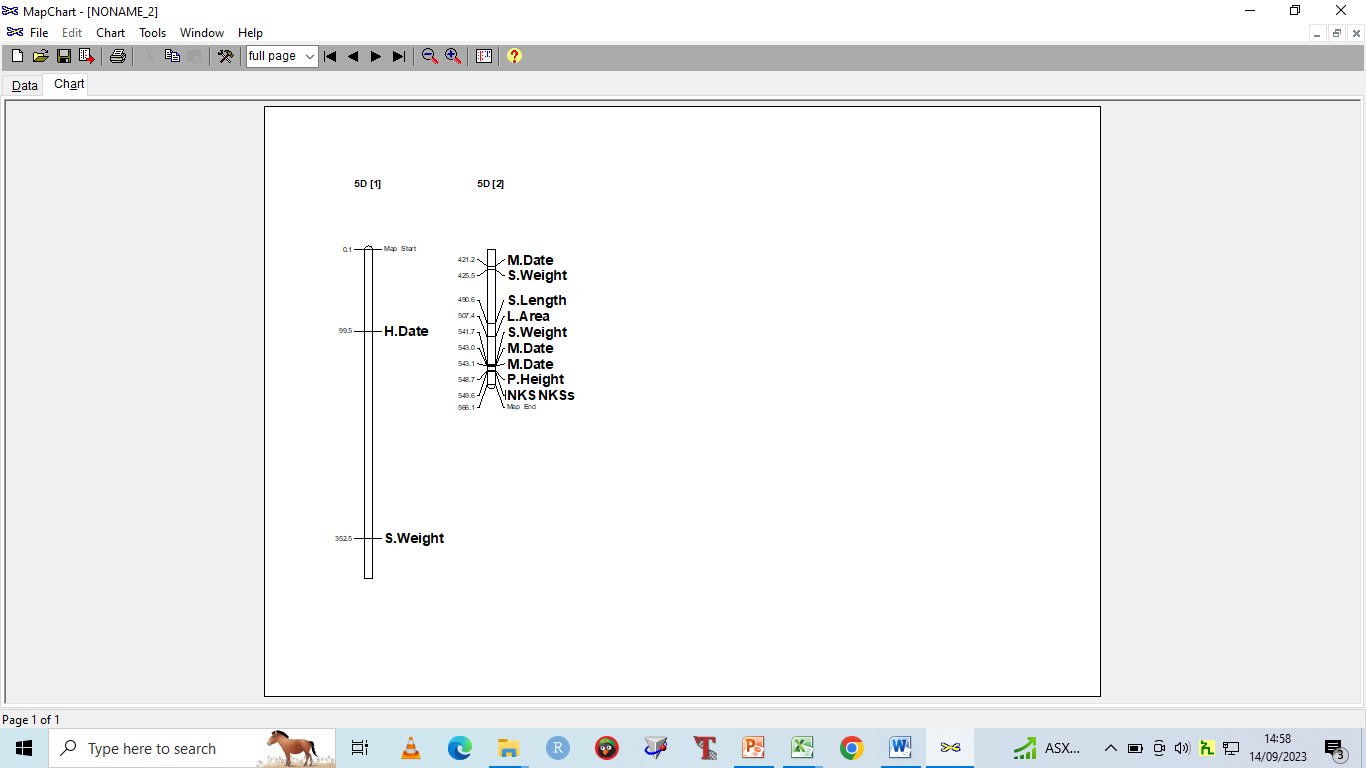

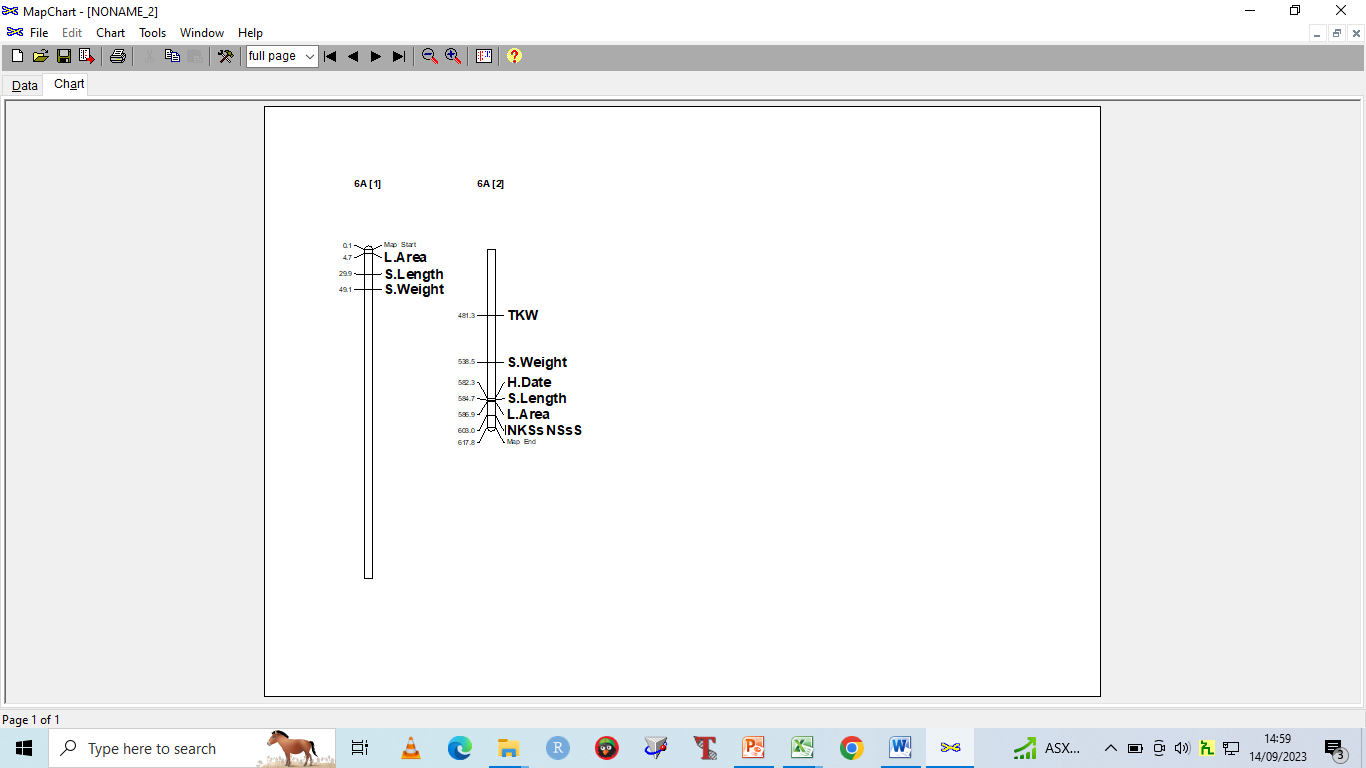


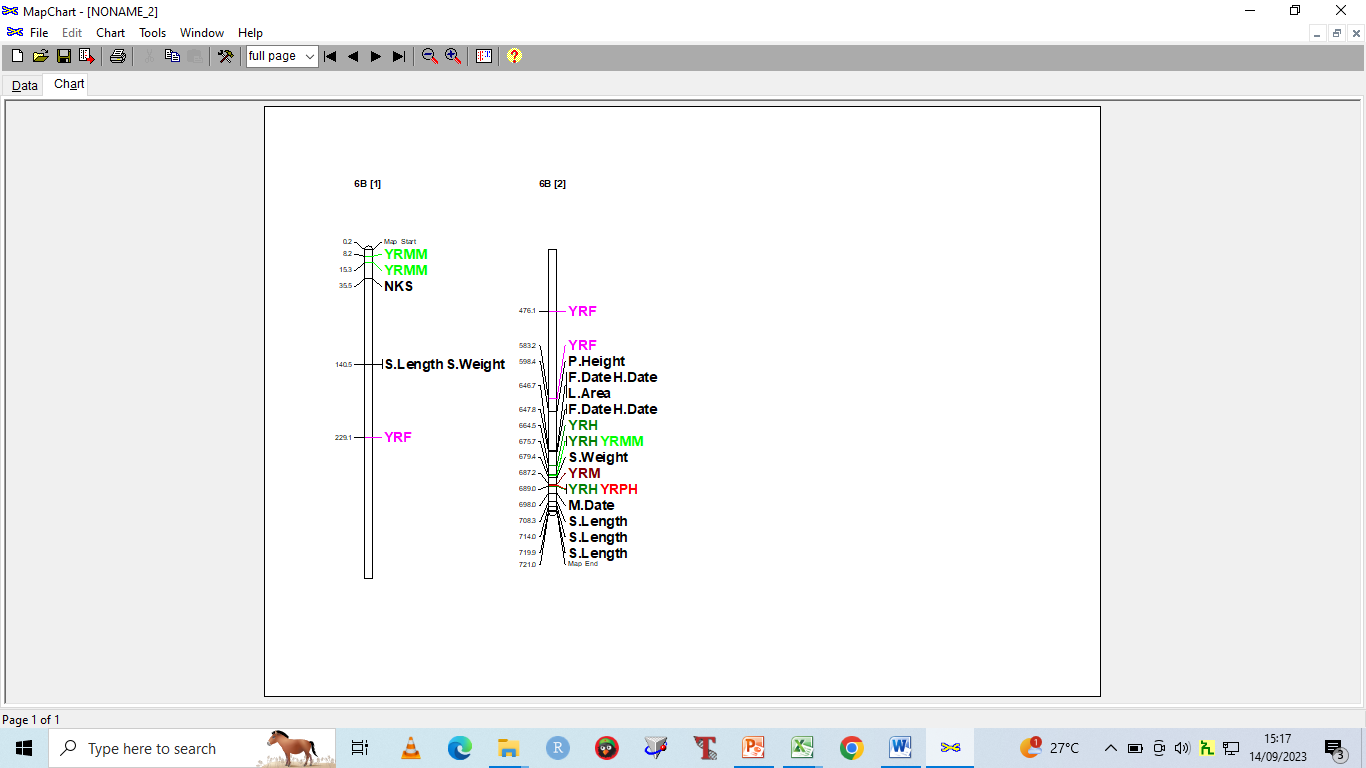

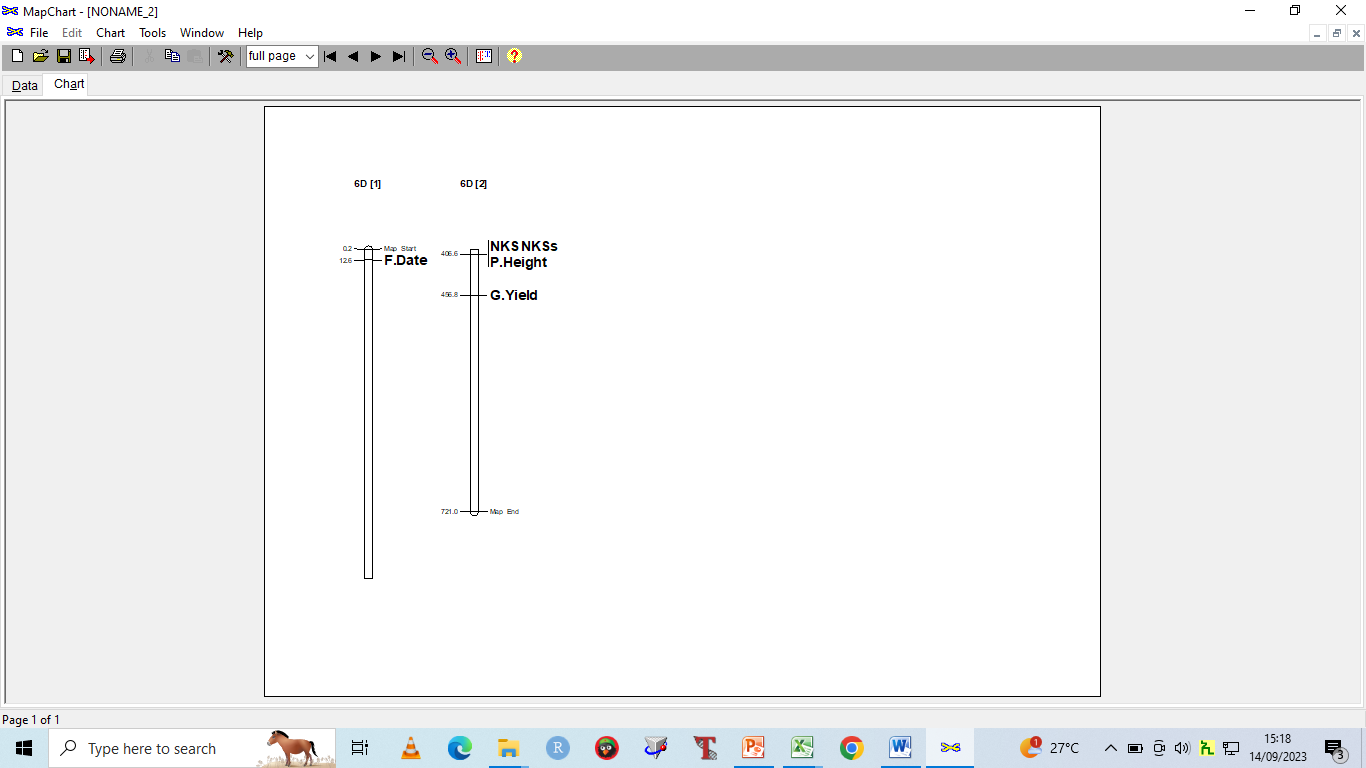

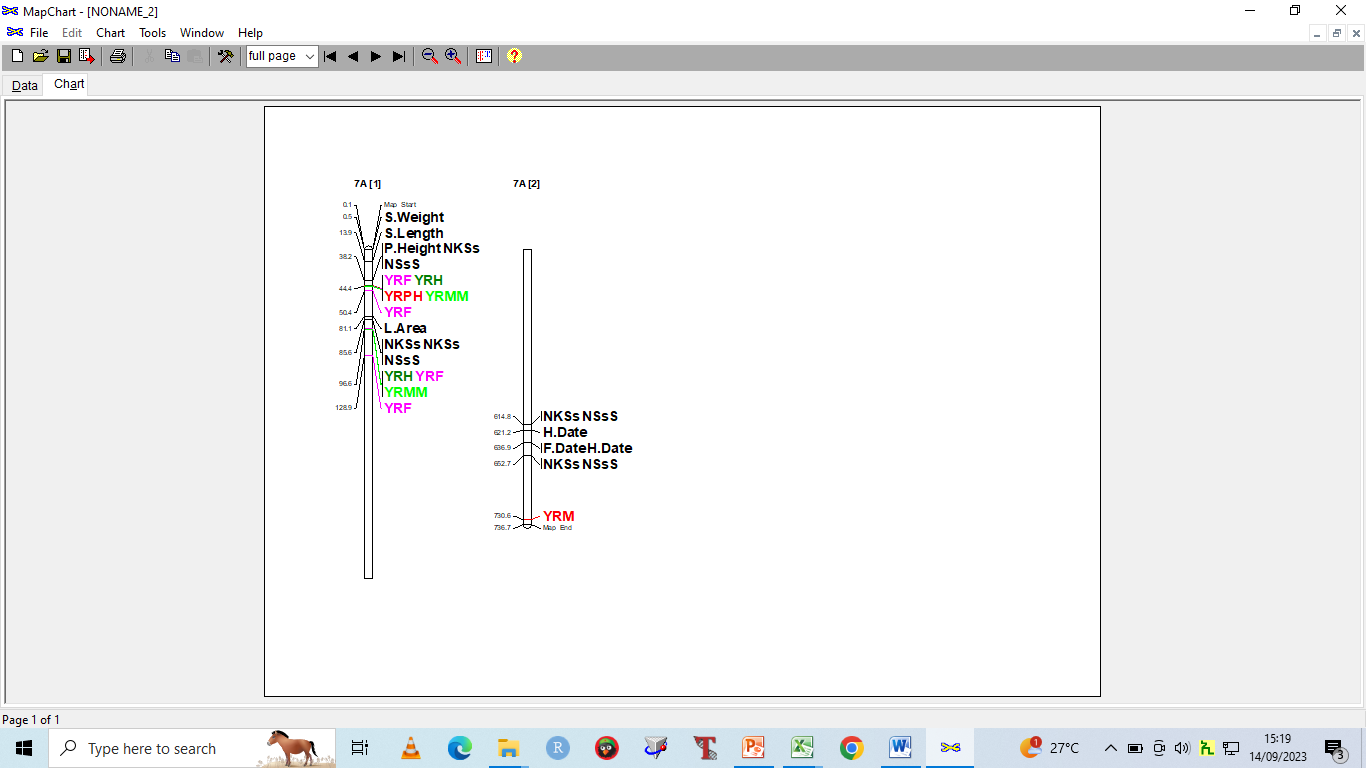


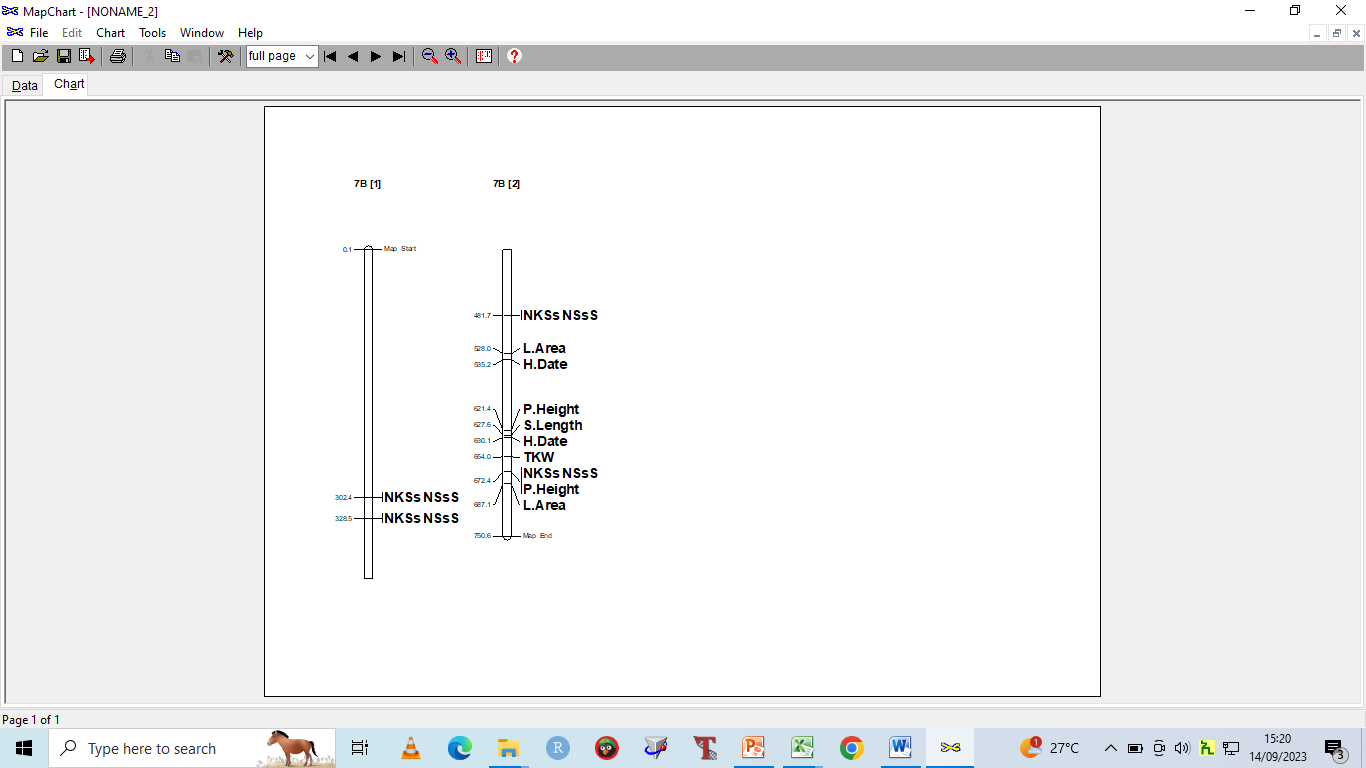

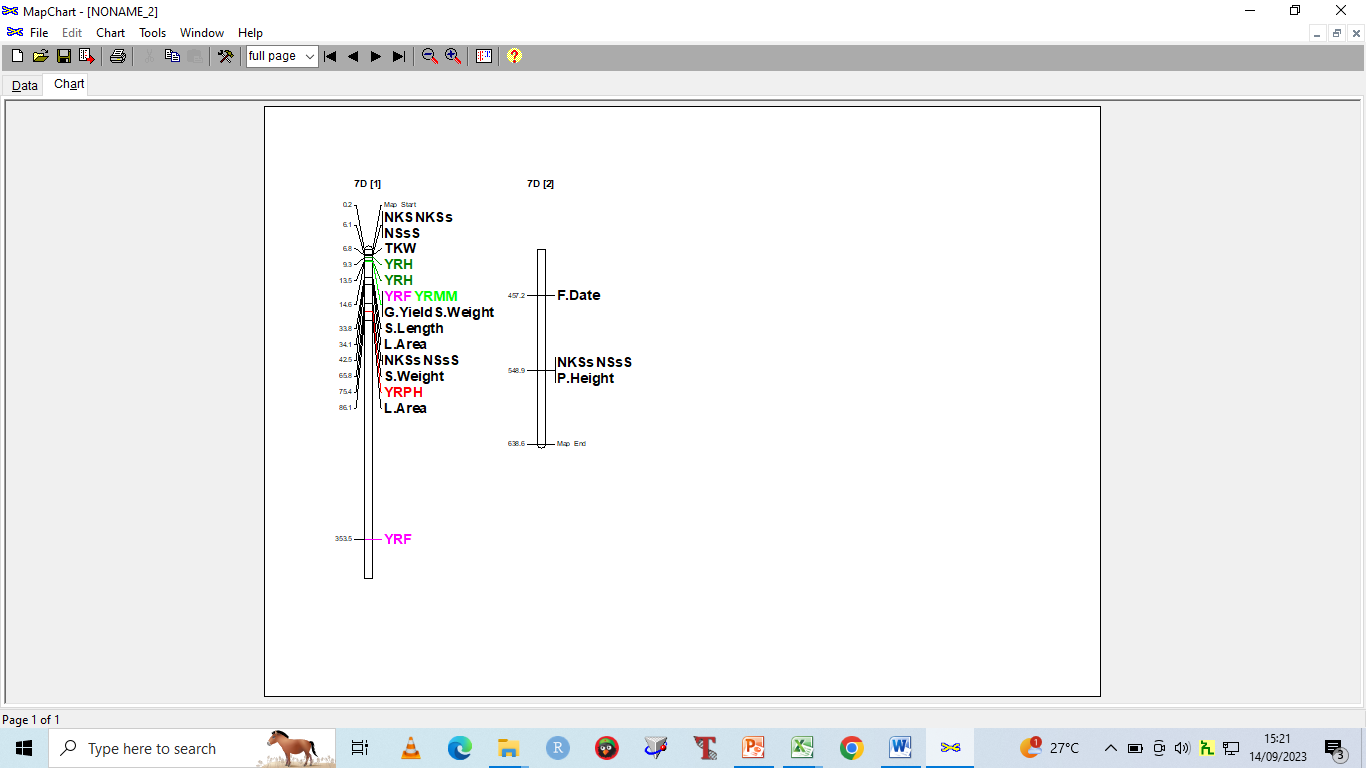


**Supplementary Figure 2**. Genomic positions of detected putative QTLs effective for yellow rust resistance. Significant DArTSeq SNPs are presented according to their physical positions on chromosomes in million base pairs. The putative QTLs identified in this study for the MTAs are indicated on the right sides of the bars. YRPH = coefficient of infection at pre-heading, YRH = coefficient of infection at heading, YRF = coefficient of infection at flowering, YRMM = coefficient of infection at mid-maturity, YRM = coefficient of infection at maturity, HD= days to heading, FD= days to flowering, DM= days to maturity, TKW= thousand kernel weight, LA= leaf area, PH= plant height, SL= spike length, NSs/S= number of spikelets per spike, NK/S= number of kernels per spike, NK/Ss= number of kernels per spikelets, SW= spike weight, and GYPP=grain yield per plot.
