# Supplementary material for: Genetic architecture of adult-plant resistance to stripe rust in bread wheat (Triticum aestivum L.) association panel
Source: Front Plant Sci. 2023 Dec 7;14:1256770. doi: 10.3389/fpls.2023.1256770 (PMC10733515; doi:10.3389/fpls.2023.1256770)
Supplement: Supplementary file 3 [file DataSheet_3.docx]

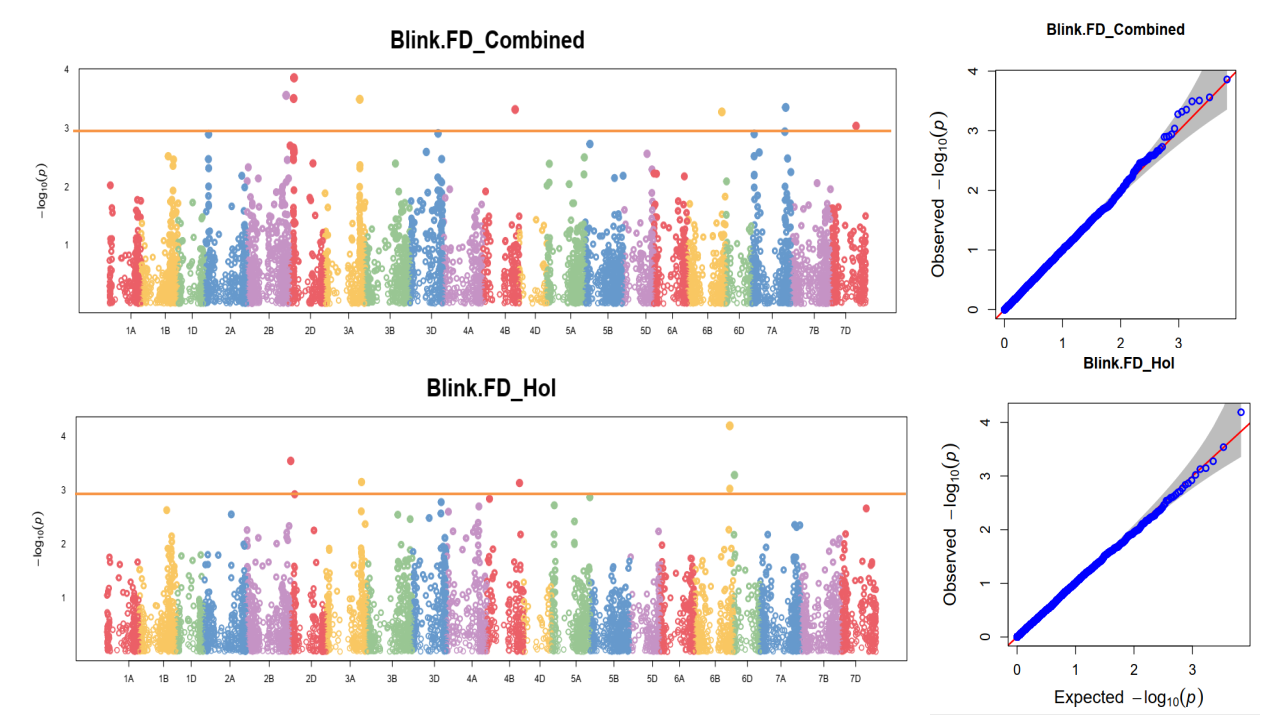


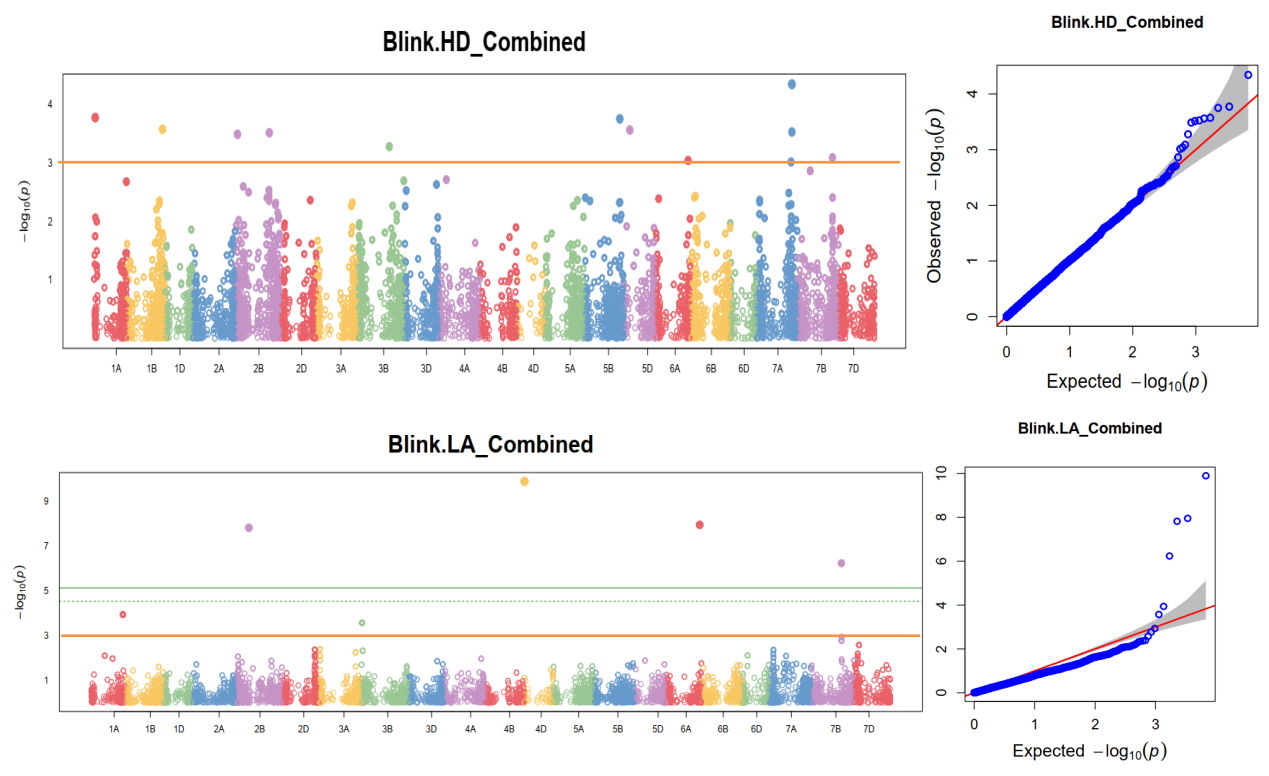


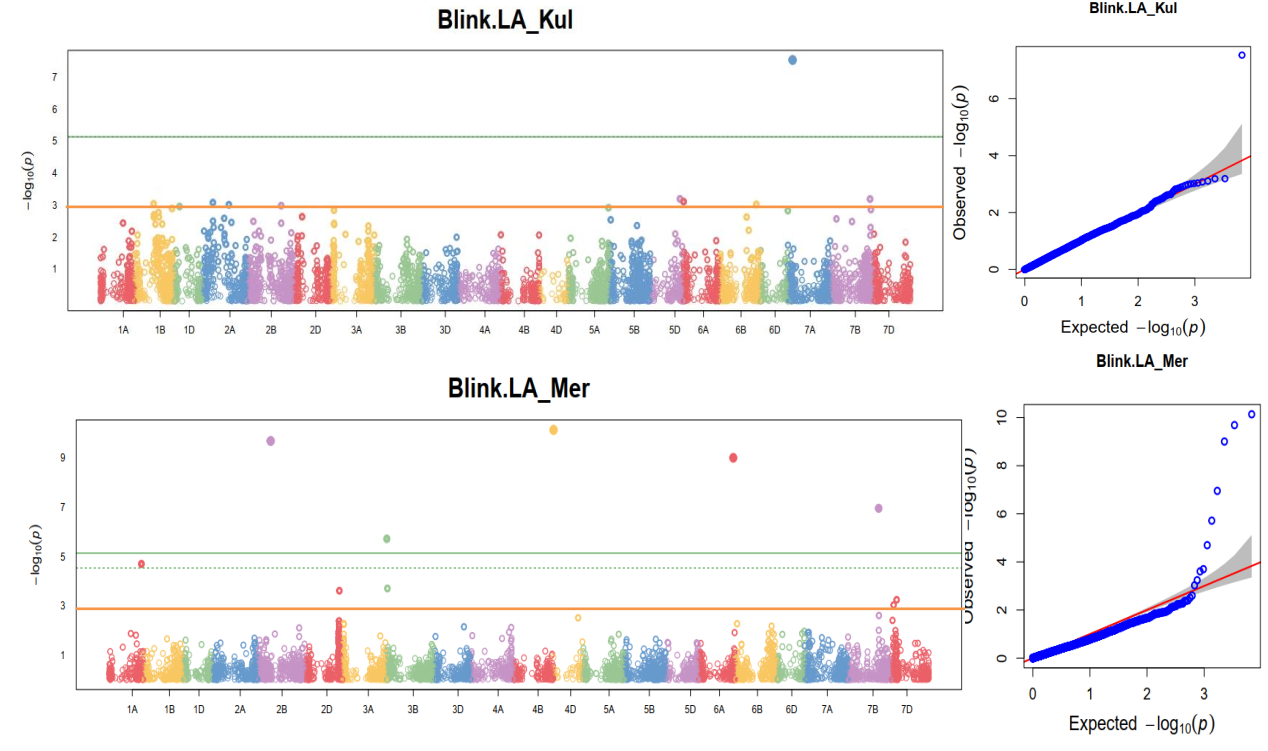


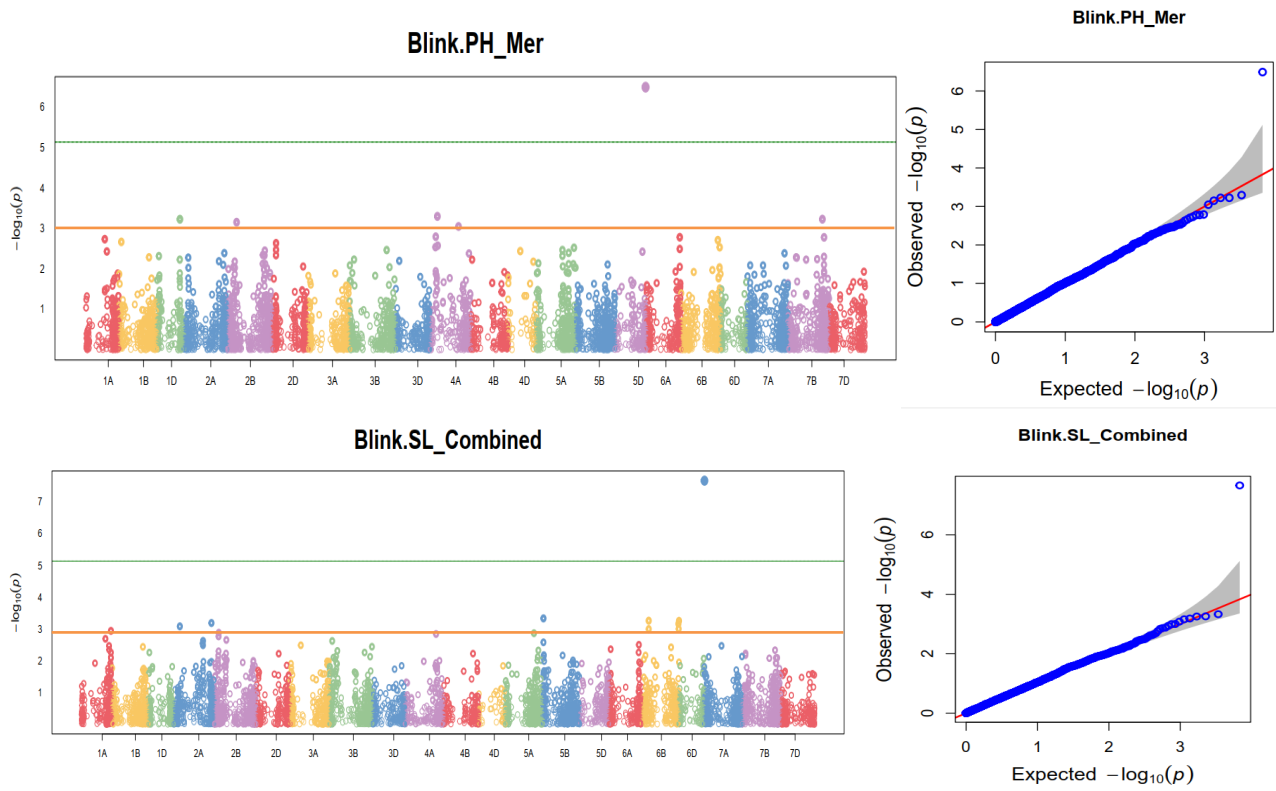


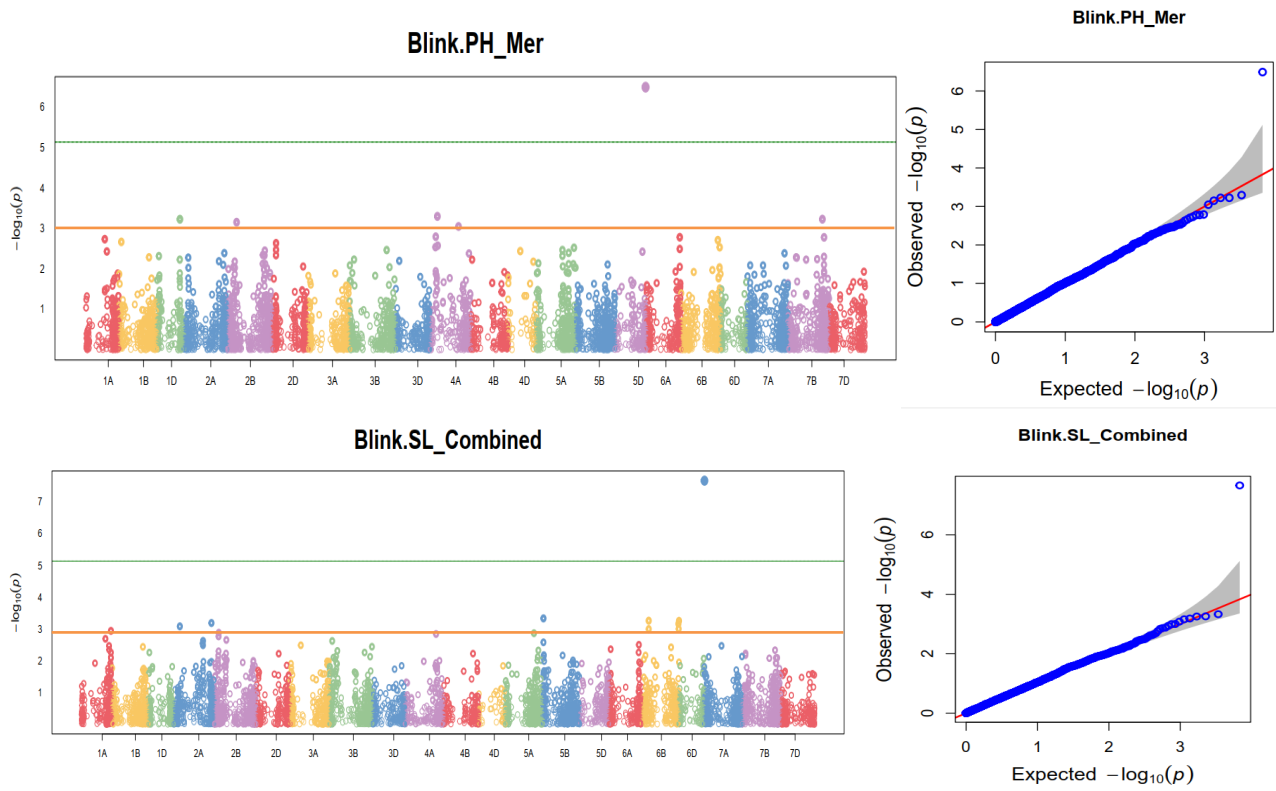


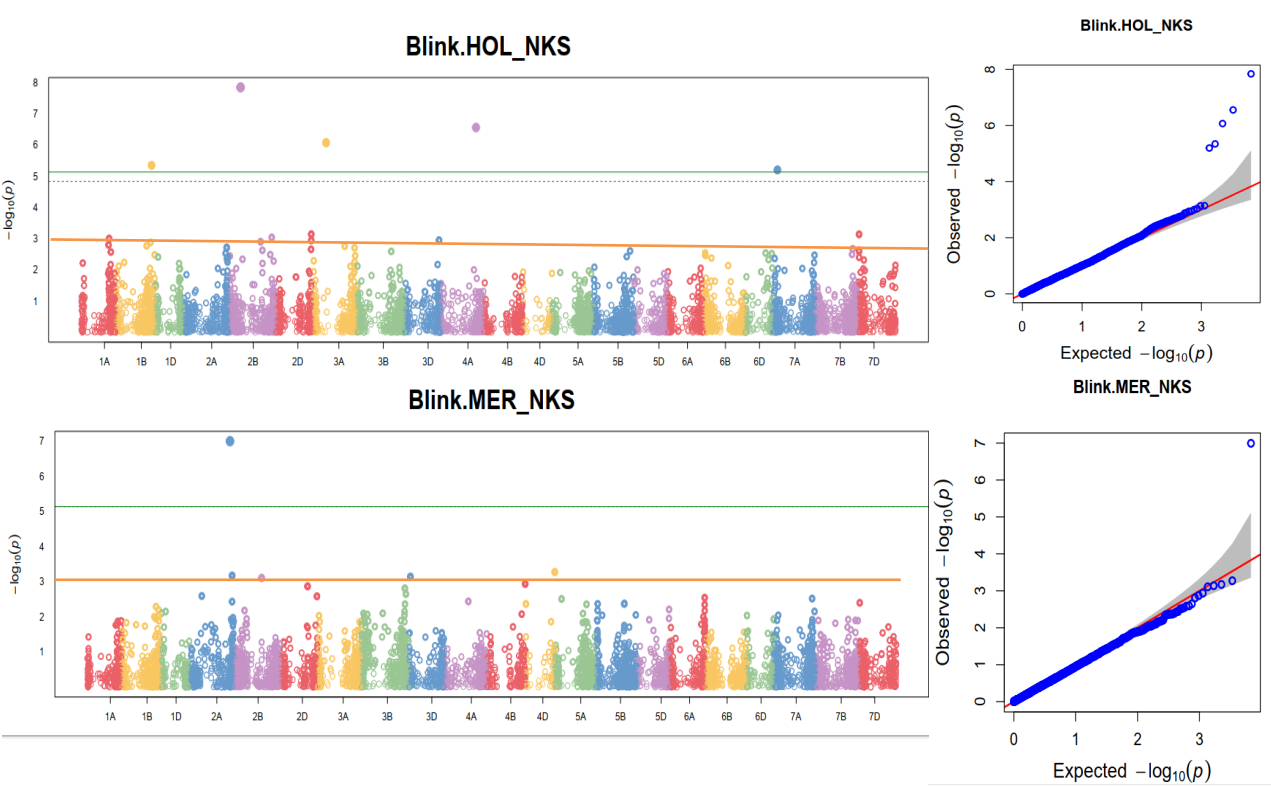


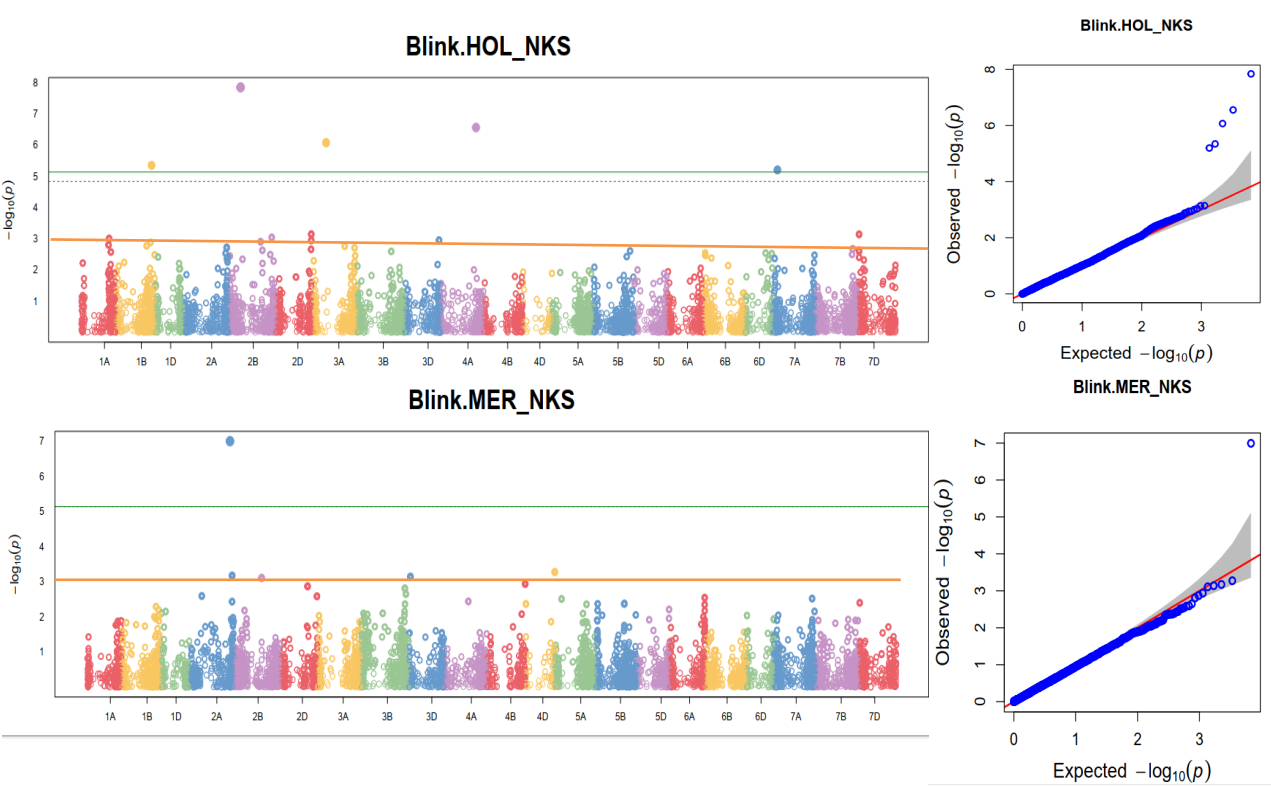


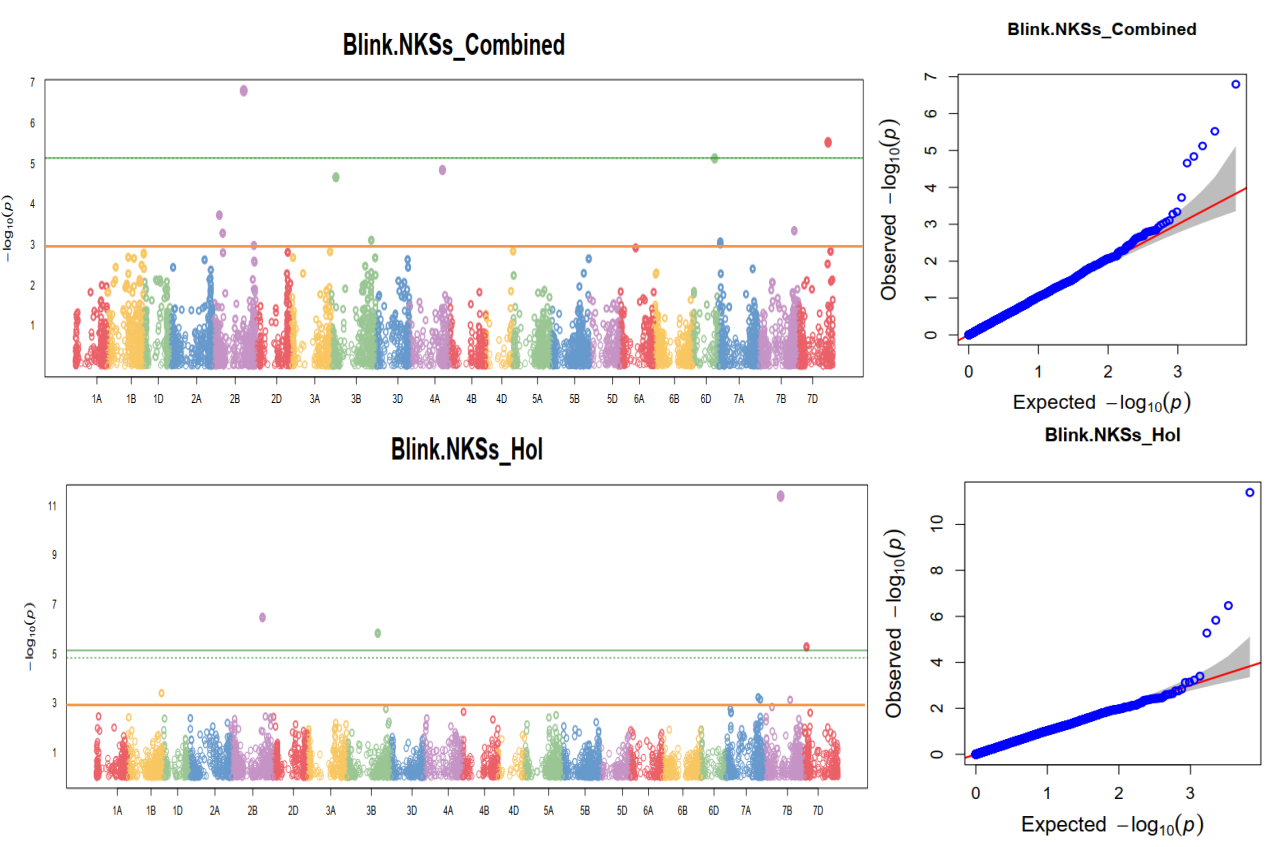


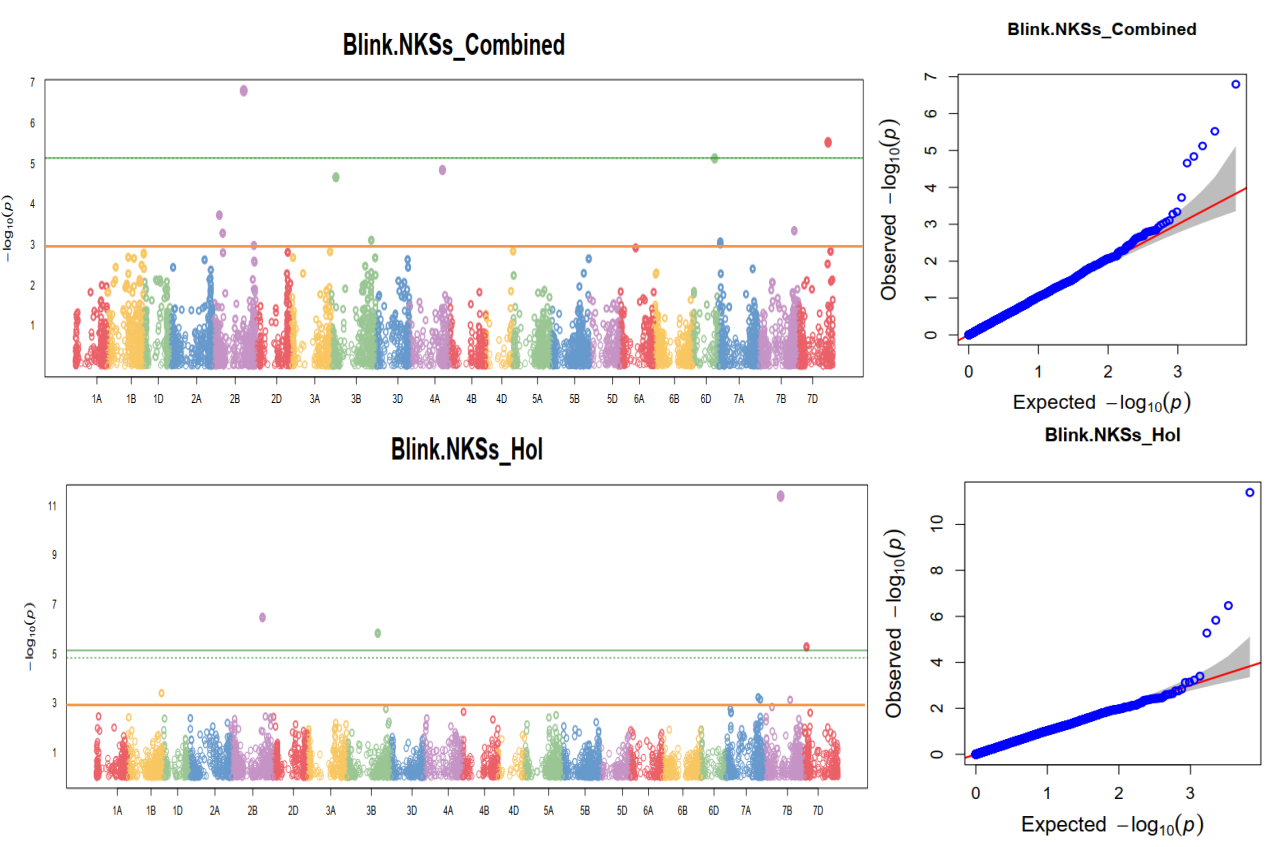


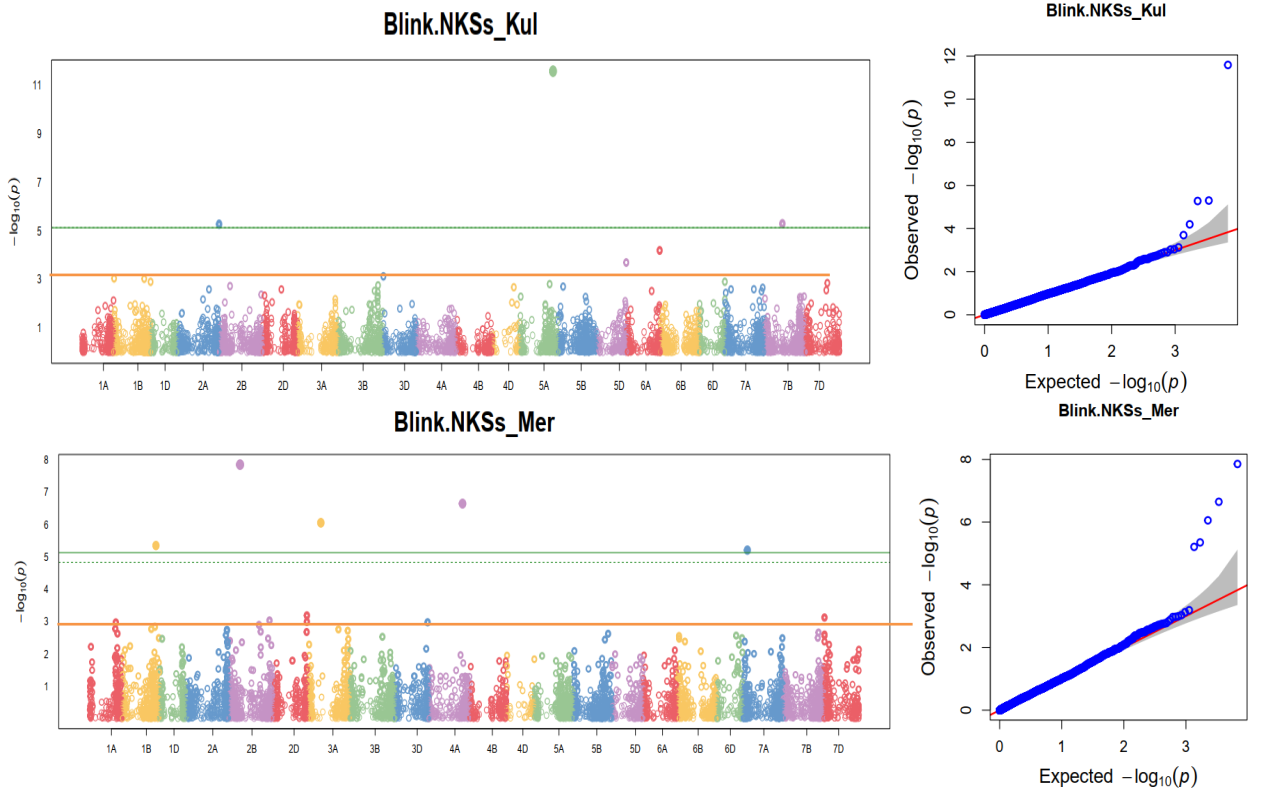


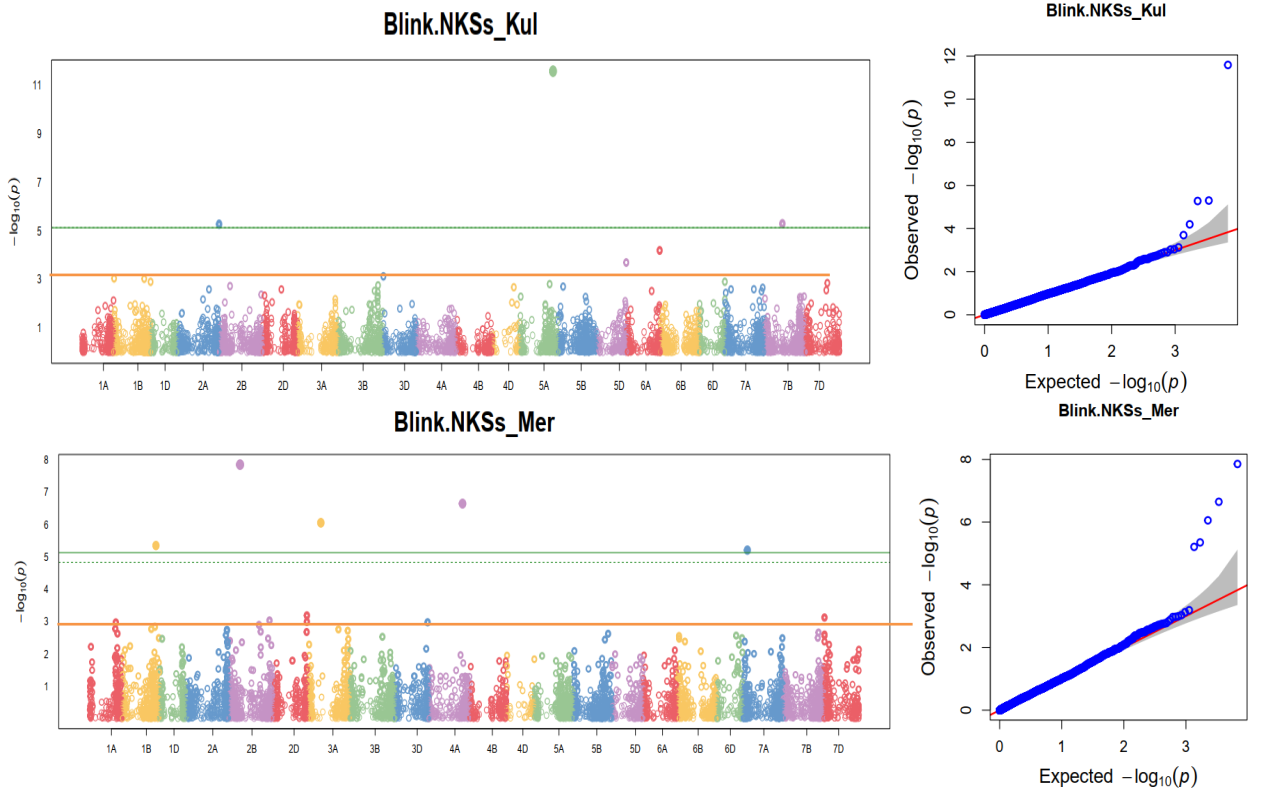


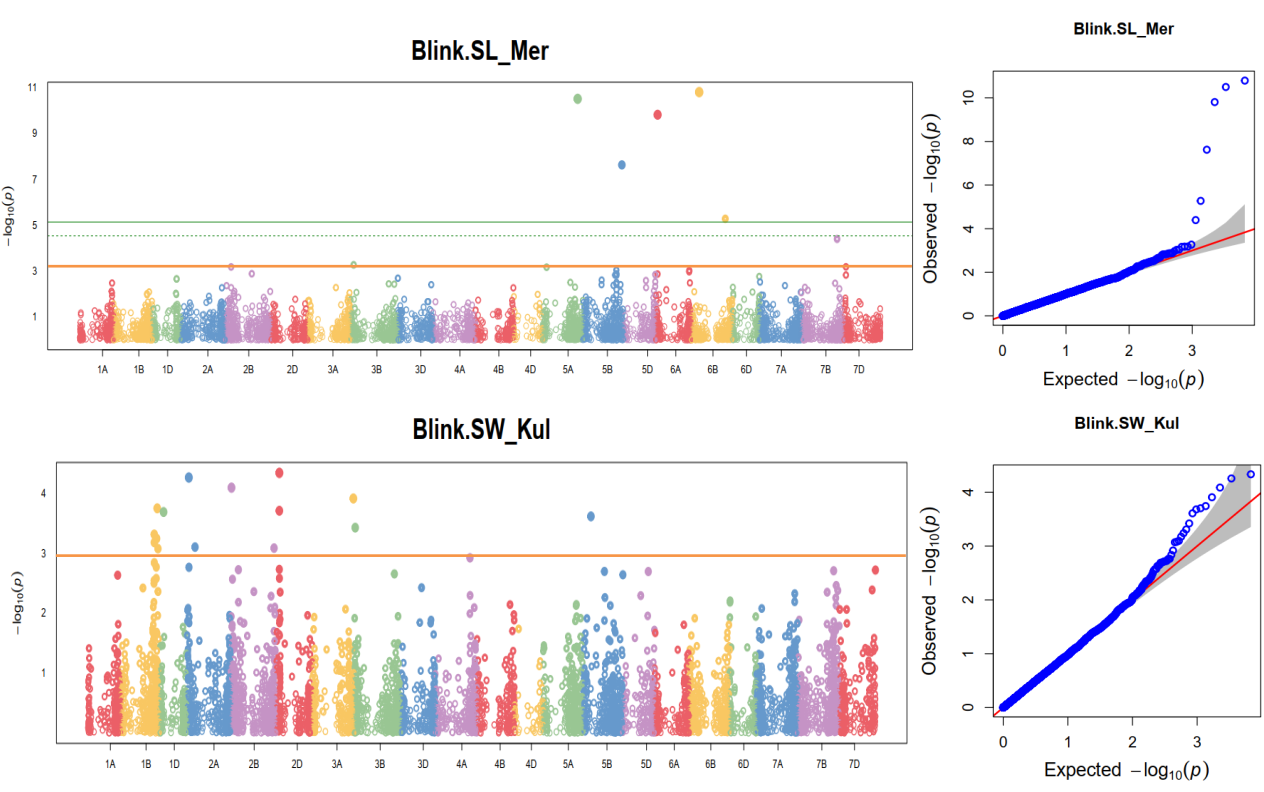


**Supplementary Figure 3.** Manhattan plots for agronomic and yield related traits each environment and combined data. HD= days to heading, FD= days to flowering, DM= days to maturity, TKW= thousand kernel weight, LA= leaf area, PH= plant height, SL= spike length, NSs/S= number of spikelets per spike, NK/S= number of kernels per spike, NK/Ss= number of kernels per spikelets, SW= spike weight and GYPP=grain yield per plot. The quantile-quantile plots at the right side of the Manhattan plots indicate how well the GWAS model accounted for population structure and kinship for each of the disease traits. In each plot, the observed *–log (p-values)* from the fitted GWAS models (y-axis) are compared with their expected value (x-axis) under the null hypothesis of no association with the trait. Each blue dot represents a single nucleotide polymorphism; the orange line is the model for no association.
